# Vertebrate and Invertebrate Animal and New In Vitro Models for Studying *Neisseria* Biology

**DOI:** 10.3390/pathogens12060782

**Published:** 2023-05-30

**Authors:** Michael M. Girgis, Myron Christodoulides

**Affiliations:** 1 *Neisseria* Research Group, Molecular Microbiology, School of Clinical and Experimental Sciences, Faculty of Medicine, University of Southampton, Southampton SO16 6YD, UK; m.m.k.girgis@soton.ac.uk; 2 Department of Microbiology and Immunology, Faculty of Pharmacy, Mansoura University, Mansoura 35516, Egypt

**Keywords:** *Neisseria meningitidis*, *Neisseria gonorrhoeae*, vertebrate, invertebrate, vaccine, immunology, animal model, infection

## Abstract

The history of *Neisseria* research has involved the use of a wide variety of vertebrate and invertebrate animal models, from insects to humans. In this review, we itemise these models and describe how they have made significant contributions to understanding the pathophysiology of *Neisseria* infections and to the development and testing of vaccines and antimicrobials. We also look ahead, briefly, to their potential replacement by complex in vitro cellular models.

## 1. Introduction

To date, about 40 *Neisseria* species have been reported (*Neisseria* isolates (pubmlst.org)) within the genus *Neisseria* (Class B-proteobacteria, Order *Neisseriales*, Family *Neisseriaceae*). These Gram-negative bacteria have been isolated from humans, other mammals and even from the environment [1]. They are considered as constituents of the normal, commensal microbiota on mammalian mucosal surfaces. Of these species, two are human-restricted: *Neisseria gonorrhoeae* (the gonococcus) is an obligate pathogen and *Neisseria meningitidis* (the meningococcus) can be regarded as a commensal organism and opportunistic pathogen that can cause overt disease [2]. Gonococci primarily colonize the mucosal epithelium of the male urethra and female endo/ectocervix, causing the sexually transmitted disease gonorrhoea [3,4]. Meningococci colonize the nasopharyngeal mucosa and can become potentially invasive and cause cerebrospinal meningitis and septicaemia [5,6]. However, atypical infections by both gonococci and meningococci have been reported, often at other anatomical sites, as well as infections with commensal *Neisseria* species behaving as opportunistic pathogens [7]. Worldwide, there are ~87 million cases of gonorrhoea reported annually [8], with the highest burden in the least developed and low-to-middle income countries on the Development Assistance Committee List of Official Development Assistance Recipients. By contrast, cases of meningococcal meningitis and septicaemia have fallen dramatically because of global uptake of meningococcal vaccines. The lack of an effective licensed gonococcal vaccine is a significant bottleneck in disease control and the ability of gonococci to develop resistance to every class of antibiotic introduced means that treatment relies on an ever-dwindling arsenal. 

In the current review, we examine the different vertebrate and invertebrate animal models that have been used in studies of *Neisseria* pathobiology and for the development of vaccines and new antimicrobials. By virtue of the considerable number of studies using normal mice, rats and rabbits for *Neisseria* research, these will be discussed in brief and information assembled in supplementary tables with accompanying bibliographies. Focus will be on those important models that try to mimic several different aspects of natural human infections. In addition, we look ahead and discuss alternatives to these models with a view to possible Replacement, Reduction and Refinement (the 3Rs).

## 2. Vertebrate Animal Models

The history of *Neisseria* research has involved the use of a wide variety of vertebrate animal models, and we begin with studies involving humans.

### 2.1. Humans

#### 2.1.1. *Neisseria gonorrhoeae* Studies

Controlled human infection with *N. gonorrhoeae* has a long-recorded history in clinical and laboratory medicine, predating even the discovery of the organism by Albert Neisser in 1879 [9]. For example, the Austrian ophthalmologist Joseph F. Piringer inoculated the eyes of blind people with ‘blennorrheal’ (gonorrhoeal) pus, some 38 years before the discovery of the gonococcus by Neisser, as an attempt to cure those patients whose eyes had been damaged by trachoma [10,11]. Benedek reviews the research on ocular inflammation associated with gonorrhoea and studies from the C18th to early C20th on the experimental induction of gonorrhoeal ophthalmia in humans, following the observations that animals were refractory to gonococcal infection [10]. As early as 1835, the British surgeon John Hunter described his repeated inoculations of human patients with ‘venereal matter’ to test the hypothesis that venereal disease had an infectious aetiology [12].

Neisser’s identification of the gonococcus and its culture in the laboratory by the Frenchman Frédéric Weiss in 1880 [13] and the German Leo Leistikow in 1882 [14,15] led to decades of experimental gonococcal infection studies in humans that became central to the development of clinical research ethics [10]. Neisser could not satisfy Koch’s postulates with his isolate, but this was achieved by the challenge experiments of the Hungarian physician Arpad Bokai, the German physician Max Bockhart and the Austro-German gynaecologists Ernst von Bumm and Ernst Wertheim. In 1880, Bokai inoculated six medical students urethrally with gonococcal culture fluid and reported that three of the patients developed acute gonorrhoea [16]. In 1883, Bockhart inoculated the urethra of a man and reported the development of classical gonococcal urethritis after three days [17]. In 1885, von Bumm grew axenic cultures of Neisser’s gonococcus and proved that it causes gonorrhoea (urethritis) by instilling the pathogen into humans [18,19]. This experimental urethritis was reproduced by Wertheim in 1891 [20]. In 1893, Steinschneider, working in Neisser’s laboratory, induced gonorrhoea in a colleague following urethral instillation of bacteria [21]. In the same year, the Austrian dermato-venerologist Ernst Finger and his colleagues examined whether previous gonorrhoea infection conferred immunity against re-infection. In their pioneering experiment, gonococci were instilled into the urethra of six men who had a history of gonorrhoea but were currently ‘healthy’. Each subject subsequently developed gonorrhoea, leading Finger to conclude that ‘the gonorrhoeal process is capable of re-infection and super-infection’ [22]. Other inoculation experiments by the American surgeon Edward Martin in 1982 [23], the American paediatrician Henry Heiman in 1895 [24], the Swedes Jundell and Ahman in 1897 [25] and the Italian clinician Guido Bordoni-Uffreduzzi in 1894 served to reinforce the now obvious conclusion that gonococci experimentally instilled into the urethra can induce gonorrhoea! By the turn of the C20th, these human inoculation experiments with gonococci were essentially abandoned as new pathophysiological findings were not being reported. Furthermore, the justifiable ethical criticism of many of these and earlier studies described above was a major factor in their discontinuation, given that some were especially unpalatable, e.g., the application of gonorrhoeal cultures to the eyes of sick children [10] and Heiman’s instillation of gonococci into the urethra of intellectually disabled children aged 4 and 16. Indeed, the emergence of ethics in human research owes a great deal to the way these human challenge experiments were performed. 

The *N. gonorrhoeae*-controlled human infection model (Ng CHIM) involves urethral inoculation of males that results in urethritis. It is the most relevant model of natural gonococcal infection that is available today, but only for men; women cannot be infected with gonococci because of the possibility of infection ascending into the upper reproductive tract and causing pelvic inflammatory disease (PID) and other complications. The Ng CHIM was developed during the 1980s by researchers at the Walter Reed Army Institute of Research and at the University of North Carolina, Chapel Hill, and is reviewed extensively by Waltmann et al. [26]. A step-by-step experimental protocol is available [27], and has been used for both vaccine and pathogenesis studies. The model is safe, and several hundred men have volunteered for experimental infection over the past decades and shown no severe adverse reactions.

The Ng CHIM was used prior to the only human gonorrhoea vaccine trials. Brinton et al. showed that a purified single antigen pilus vaccine protected human volunteers in the Ng CHIM from infection with the homologous strain (rate of protection ~50%), and induced serum and genital antibodies [28]. However, the vaccine showed no protection against a heterologous strain expressing antigenically variant pili in a subsequent human trial [29], and no protection in a large-scale field trial in which it was used to vaccinate high-risk US military personnel stationed in Korea [30]. The Ng CHIM was also used to test a gonococcal vaccine composed of isolated PorB/Outer Membrane Vesicle (OMV), but contaminated with lipooligosaccahride (LOS), Rmp and Opa protein. The findings from this study with the Ng CHIM were that immunity against PorB, LOS and Opa ‘transpired theoretically to predict protection from infection’, whereas Rmp was subversive for PorB immunogenicity in humans and enhanced the likelihood of infection [31,32]. In contrast to active immunization, there is one recent report of a passive protection study using the Ng CHIM to test the hypothesis that a human monoclonal antibody raised to β-(1-6)-linked poly-N-acetyl-d-glucosamine (PNAG), and bactericidal for gonococci, could prevent gonococcal infection [26]. Infusion of this monoclonal antibody did not reduce bacterial load in the urine of infected volunteers, with four out of seven infused volunteers, and one out of three control volunteers, i.e., without antibody infusion, developing urethritis. The experiment has its flaws and is perhaps too under-powered to draw any definitive conclusions, and certainly does not exclude a potential protective role for polyclonal antibodies and/or a cell-mediated immune response. 

The Ng CHIM model has been used in pathogenesis studies, and this is reviewed in detail elsewhere [33]. Briefly, the two most often used strains in the Ng CHIM have been *N. gonorrhoeae* FA1090 and MS11mcK, with discernible differences between the two, e.g., in serum sensitivity, presence of gonococcal genetic island, lactoferrin utilization, Mtr efflux pump expression and (hyper)piliation [33]. The earliest report of a link between gonococcal virulence and clonal phenotypic variation in infected human volunteers was from Kellogg and colleagues in 1963 [34]. The course of infection has been extensively characterised microbiologically and clinically with descriptions of transmission [35], inflammatory processes [36], gonococcal infection and reinfection [37], changing population dynamics and antigen variation, e.g., in pili and Opa expression. The model has demonstrated the effects of LOS [33,38,39,40], Opa [41,42], pilE pilin [43,44,45,46], IgA protease [47] and the iron-binding proteins lactoferrin and transferrin [48,49] during natural infection. 

At present, there is a pipeline of candidate gonococcal vaccine antigens that are being tested for antigenicity in rodents and rabbits for their ability to generate bactericidal antibodies, before moving to testing in the female mouse genital tract infection model. None of these antigens have yet been tested in the Ng CHIM, and the model does have some limitations in use; these include difficulty in recruiting sufficient volunteers to provide statistical significance during vaccine trials (especially if several antigens are promising), the probable need to challenge with several heterologous strains to determine broad vaccine coverage, and the lack of known correlates of protection and mechanisms of clearance. Nevertheless, success in the Ng CHIM must be a pre-requisite for larger vaccine field trials.

#### 2.1.2. *Neisseria lactamica* Studies

A CHIM has been developed using *N. lactamica* [50], and in its first published use in 2011, the model showed that intranasal inoculation of *N. lactamica* resulted in colonization and carriage of the organism. In addition, colonization induced mucosal and systemic humoral immunity to *N. lactamica* and cross-reacting opsonophagocytic antibodies to meningococci. However, bactericidal antibodies were not induced, and, not unexpectedly, meningococcal acquisition was not inhibited by the presence of humoral immunity to *N. lactamica* [51]. Subsequent work showed that nasal inoculation of *N. lactamica* statistically reduced meningococcal carriage from 24.2% at inoculation to 14.7% at 2 weeks after inoculation [52] and that natural immunity to meningococci after colonisation with *N. lactamica* might be due to cross-reactive adaptive responses [53]. In addition, *N. lactamica* bacteria undergo microevolutionary changes during nasopharyngeal colonization [54]. More recently, *N. lactamica* were engineered to express the meningococcal Bexsero vaccine antigen *Neisseria* Adhesin A (NadA). Intranasal inoculation generated NadA-specific IgG- and IgA-secreting plasma cells within 14 days of colonization, NadA-specific IgG memory B cells within 28 days of colonization, and, importantly, serum bactericidal antibody activity against NadA-expressing meningococci [55]. This study suggests that intranasal inoculation of genetically modified bacteria could be a useful vaccine platform to generate potentially protective immune responses to heterologous antigens from different bacterial pathogens.

#### 2.1.3. *Neisseria meningitidis* Vaccine Studies

There is no meningococcal controlled human infection model (Nm CHIM), due to concerns over the rare possibility of systemic disease following instillation of live meningococci into the nasopharynges. Nor is one needed for vaccine studies, given the global success of the ACYW capsular polysaccharide-conjugate vaccines and the serogroup B Bexsero OMV and Trumenba vaccines. The success of these vaccines owes a great deal to the innumerable phase I, II and III trials carried out over the years.

### 2.2. Non-Human Primates (NHPs)

NHPs are the animals most closely related to humans, and have seen some use for studying immune response to vaccines and modelling human disease(s) [56]. The most used NHPs have been chimpanzees, baboons and old-world monkeys such as the rhesus macaque, the first two sharing 95–96% similarity of their entire genome with humans, and the latter 93%. 

#### 2.2.1. Chimpanzee (*Pan troglodytes*)

The first historical attempts to infect NHPs to try to reproduce gonococcal urethritis involved instilling pus from patients with gonococcal urethritis or with saline suspensions of gonococcal cultures, and date back to the 1880s–1930s. They were unsuccessful [14,57,58]. In 1971, Lucas et al. reported the first established infection of chimpanzees with gonococci [59]. In this study, urethral exudate from human males with gonococcal urethritis was instilled into the urethras of three male chimpanzees, who subsequently developed gonococcal urethritis. Notably, gonococcal urethritis was transferable from chimpanzee to chimpanzee and one chimpanzee developed gonococcal conjunctivitis. All the infected animals developed serum complement-fixing antibodies to gonococci. A year later, Brown et al. showed that in vitro passaged gonococci could still infect chimpanzees and provided the first report of natural transmission of gonorrhoea from male to female chimpanzee [60]. These same authors reported that one male chimpanzee, who had been a chronic nasopharyngeal carrier of meningococci for a year, frequently engaged in self fellatio with the result that meningococci were isolated from his urethra [61]. 

The chimpanzee model mimics human gonococcal infection in signs, symptoms, and host response, and in the development of natural resistance to gonococcal challenge [62]. Kraus et al. inoculated the urethra, pharynges and cervix of chimpanzees with gonococci isolated from men with urethritis, and then re-challenged the animals one week after termination of the initial infection. The study demonstrated that (1) after natural infection, the cervix and pharynx resisted more gonococci than the urethra, (2) anatomical and environmental factors influenced resistance to gonococcal pharyngitis, (3) chimpanzees acquired post-infection immunity, (4) following gonococcal pharyngitis, the pharynx resisted more gonococci than were initially resisted and (5) more gonococci were successfully resisted in the re-challenged urethra. Their conclusion was that acquired immunity following gonococcal infection suggested that a vaccine approach may be of value in controlling the gonorrhoea pandemic. Indeed, the subsequent papers from Arko and colleagues from 1974 to 1977 tested the feasibility of crude gonococcal vaccines in the chimpanzee. Arko et al. immunized four male chimpanzees intramuscularly with a formalin-killed vaccine prepared from a gonococcal isolate virulent for chimpanzees. They showed that vaccination greatly increased urethral resistance to infection with the homologous isolate [63]. Resistance was dependent on the dose of urethral challenge, with higher bacterial doses leading to urethral infection, but this was significantly shorter in duration and accompanied by substantially lower numbers of gonococci recovered from urethrae, compared to unimmunized chimpanzees. Notably, immunized chimpanzees were resistant to re-infection with the homologous isolate, but not to a heterologous isolate. In an expanded serological trial of the formalin-killed vaccine, the serological response (indirect fluorescent antibody and serum bactericidal activity measurements) correlated with the resistance of *n* = 5 vaccinated individual chimpanzees when they were challenged in the pharynx and urethra with live gonococci, compared with *n* = 5 sham controls [64]. After this flurry of papers, research with chimpanzees effectively came to an end in the reported literature. Even though urethral infection in chimpanzees anatomically resembles human infection and is biologically more accurate, the utility of the model for immunological and vaccine research is limited due to their high cost and the ethical issues around their use, as well as the fact that they are outbred animals that tend to have more variable individual immune responses, which are compounded by the presence of interfering micro-organisms in the urethra. 

#### 2.2.2. Rhesus Macaque (*Macaca mulatta*)

Rhesus macaques (RMs) are also frequently used to model human disease, including infection with *Neisseria.* Indeed, macaques are naturally infected with a *Neisseria* spp., *N. macacae* [65], whose genome contains genes encoding for many proteins that are components of current meningococcal vaccines and are being studied as candidate gonococcal antigens [66]. Infant macaques are frequently used to test immune responses to *Neisseria* vaccine antigens when tested alone or when used as adjuvants with other vaccines. These are summarised in Table 1. The model has been used to extensively study the molecular mechanisms of colonization, transmission, persistence and horizontal gene transfer of *N. macacae* [66], and antimicrobial resistance in the pharynx [67]. It has also been used to study heterogeneity and affinity of monkey complement factor H binding to fHbp [68,69,70], to inform primate selection for assessing vaccine immunogenicity. Several other studies have used rhesus serum in vitro, and not the animals directly, to analyse the mechanisms of gonococcal serum resistance [71,72,73] and interactions of primate transferrins [74].

#### 2.2.3. Other Monkeys

Several other monkey species have been used for *Neisseria* research. In an early report, inoculation of the urethra, conjunctiva, pharynx and anal canal of crab-eating macaques (*Macaca fascicularis*, cynomolgus monkey) with urethral exudate from male patients with gonorrhoea or with laboratory gonococcal strains was unsuccessful in establishing infection [93]. Cynomolgus monkeys have been used for vaccine studies, e.g., of a recombinant bivalent meningococcal rfHbp vaccine [94], the Norwegian wild-type OMV vaccine and the Dutch hexavalent PorA OMV vaccine [95] and for the meningococcal P64k protein used as a fused adjuvant with a dengue virus protein [86,96]. African Green Monkeys (*Cercopithecus aethiops*) have been used in vaccine studies for the meningococcal serogroup C capsular polysaccharide conjugated to the P64k protein [97], and baboons (*Papio* spp.) have been used for testing a chemically modified *Escherichia coli* K1 N-propionylated polysialic acid coupled to purified recombinant rPorB [81]. In addition, a male baboon infection model of gonorrhoea is redundant, given the report that baboons inoculated intraurethrally with gonococci did not produce discharge or significant neutrophil infiltration and the animals failed to shed bacteria or develop a humoral immune response [98]. However, the epididymis of some baboons was inoculated by percutaneous injection and an anamnestic antibody response was produced following urethral inoculation. The common marmoset (family *Callitrichidae*, *Callithrix jacchus*) has been used to demonstrate the interactions of anti-gonococcal antibodies with proteins in the choroid plexus and brain, in a study of the role of maternal bacterial infections during prenatal brain development. This followed the observation that prenatal maternal infections with gonococci appeared to correlate with an increased lifetime probability for the offspring to develop psychosis [99]. 

### 2.3. Mouse

*N. musculi* sp. nov. is the only species of *Neisseria* that can naturally colonize the oral cavity of the wild house mouse, *Mus musculus* subsp. *domesticus* [100]. Infecting laboratory mice with this species has been proposed as a ‘natural approach’ for studying the interactions of *Neisseriae* with the vertebrate host, and is thus potentially useful for testing the efficacy of vaccines and antimicrobials [101]. The advantages include no requirement for antibiotics and hormones (which will become apparent when discussing the mouse intravaginal model of gonococcal infection below), and no transgenetic manipulation of the host or the use of invasive procedures. In a study from Ma et al., long-term colonization, of up to a year in some mouse species, was achieved with a single oral dose of *N. musculi*. However, there was variation in susceptibility to colonization between the inbred mouse strains [101]. Thus, mouse genetics and innate immunity responses influenced susceptibility to colonization, although all colonized mice remained healthy. Expression of type IV pilus by *N. musculi* was essential for colonization of mucosae, and a judicious choice of inbred mouse to use with this species should increase our understanding of the mechanisms involved in colonization and establishment of commensalism. Surprisingly, the broader scientific community has made limited use of this model.

#### 2.3.1. Mouse Models for Pathobiology Research

The mouse is an important animal for pathobiology and infection studies with gonococci and meningococci.

- Studies with *N. gonorrhoeae*.

Infection of subcutaneously implanted chambers with gonococci has been described and used to examine gonococcal survival and neutrophil infiltration into the chamber and surrounding tissue [102]. The model has been used to show how gonococci were protected from humoral and cellular factors in the chambers only if the bacteria were inside primary tissue culture cells derived from whole 14- to 16-day-old mouse embryos [103]. This in vivo study demonstrated how an intracellular stage enables the gonococcus to evade the innate immune response. The histopathological findings in tissues within and surrounding these chambers infected with gonococci were examined for up to 30 days in mice and guinea pigs [104]. The pathological findings are similar to those of disseminated gonococci infection (DGI) in humans, with polymorphonuclear neutrophil (PMNL) influx into the chamber and adjacent tissues, tissue necrosis and haemorrhage, accompanied by a steady decrease in the presence of gonococci [104]. Early studies on complement-mediated and complement-independent mechanisms of protection have also been reported with the mouse chamber model [105]. A model of intracerebral challenge has been described, and, although it does not mimic the course of natural infection, mice died between 1 and 6 days after challenge with bacteria disseminated to the liver, kidney and spleen [106]. A model of gonococcal pneumonia has been reported in which gonococci were introduced by intranasal inoculation into the lungs of mice, with the organs becoming acutely inflamed with infiltration of bronchioles and alveoli by PMNLs. The inflammatory response and infection, however, was self-limiting and resolved within 4 days [107]. Artificial air pouches in the connective tissue of mice were evaluated for studying gonococcal infection [108]: as expected, infection is characterised by PMNL infiltration, and a persistence of intracellular bacteria in connective-tissue fibroblasts was observed. Disseminated gonococcal bacteraemia with peritonitis and fatal septicaemia has also been modelled in the mouse; this required exogenous mucin and haemoglobin, which served to increase virulence of the bacteria administered intraperitoneally [109]. This disseminated gonococcal infection model was subsequently used with endotoxin-resistant and endotoxin-susceptible mouse strains to examine the role of endotoxin responsiveness in host defence [110]. In a further study of gonococcal bacteraemia, resistance to gonococcal infection was shown to be dependent on inflammatory cells and sex of the animal [111].

However, all these models have fallen into disuse, and it is clear from the literature that wild-type mice are not naturally colonized by gonococci. However, this limitation has been obviated to some extent with the generation of ‘humanized’ mouse models through the introduction of human transgenes. For example, neutrophils from transgenic mice carrying the human carcinoembryonic antigen-related cell adhesion molecule (CEACAM) 3 gene have been shown to respond to gonococci, and that engagement with CEACAM3 stimulates the pro-inflammatory response typical of gonorrhoea [112]. These transgenic mice expressing human CEACAM3 were crossed with mice deficient in key phagocytic (Rac2), inflammatory signalling (B cell lymphoma/leukaemia 10 [Bcl10]) and Mucosa-associated lymphoid tissue lymphoma translocation protein 1 (Malt1), to study processes downstream of CEACAM3 in the anti-gonococcal responses of neutrophils [113]. These models suggested that Bcl10 in particular was a key molecule in the inflammatory cascade downstream of CEACAM3 and TLR4. Gonococci instilled intravaginally have been shown to bind to human CEACAM5 receptors expressed on the mouse urogenital tract surface, which triggered expression of CD105 (the transforming growth factor receptor) and altered cell adhesion properties and aided colonization by reducing epithelial cell sloughing [114]. Mice carrying the complete CEA gene (CEAtg) were used in a study to demonstrate that, after vaginal infection, gonococci binding to CEACAM1 suppressed the exfoliation of epithelial cells, which depended on nitric oxide (NO) release from the bacteria [115]. Notably, blocking of NO triggered cGMP-PKG signalling cascade, and CD105 expression restored sloughing of cells colonized by gonococci, hence reducing bacterial levels in the genital tract. The most recent study involving a CEACAM transgene was the introduction of the CEACAM1 transgene into BAFF-transgenic mice (a TNF-family member) to study the role of meningococcal-induced IgA production in the pathogenesis of IgA nephropathy following nasal infection [116].

It is well-documented that gonococcal infection increases the risk of HIV shedding and transmission, and an animal model would be useful to explore the interplay between virus, bacterium and host. Recently, a humanized mouse model was developed that allowed systemic HIV infection and genital gonococcal infection by grafting immunodeficient NSG mice with human CD34+ hematopoietic stem cells [117]. The model is important for demonstrating that systemic HIV challenge results in viraemia and vaginal shedding of virus and that subsequent gonococcal challenge increases viral shedding in the genital tract but does not change plasma virus levels. These outcomes mimic clinical observations, and thus the model has the potential to assess future therapies aimed at reducing viral and bacterial infection.

Today, the female mouse model of *Neisseria gonorrhoeae* intravaginal infection has become the primary model for studying some aspects of gonococcal pathogenesis and for vaccine studies. Development of the model dates to the papers of Braude [118], Streeter and Corbeil [110] and Kita et al. [119] from the early 1980s. All reported that female mice could be colonized by gonococcal instilled intravaginally when the animals were in the proestrus stage of the oestrous cycle, but that colonization was short-lived, and the bacteria readily cleared during the metestrus phase. Not unexpected, given the lack of murine host factors and the human host restriction of the gonococcus. However, this brief colonization period was extended by Taylor-Robinson et al. [120] in 1990, who showed that treating mice with the hormone 17β-estradiol increased the duration of gonococcal infection and thus presented the model for studying the pathogen–host mucosal interactions. Subsequent work from Ann Jerse’s laboratory has refined the model [121,122], and step-by-step methodology is now available [123]. The model can use many different inbred and outbred strains of mice, and bacterial colonization and clearance, neutrophil infiltration and cytokine production can all be measured directly in vaginal samples [32]. Jerse notes that two types of infections can be established: (1) competitive infections, during which the differences in fitness-to-colonize between gonococcal strains and the nature of the factors that aid colonization and persistence in the lower reproductive tract can be studied, and (2) non-competitive infections, which enable the role(s) of individual virulence factors during infection and host immune responses to be examined, and this is generally done by comparing, for example, wild-type and knock-out and/or phenotypic variants [123]. 

The model continues to play a significant role in determining in vivo the pathogenesis of gonococcal interactions with the lower genital tract mucosa, how gonococci evade host innate immunity and suppress adaptive immune responses [124,125], how they adapt to the hormonally controlled host environment and what mechanisms drive antibiotic resistance [32,126,127]. It is also an important model for testing the efficacy of an ever-increasing number of new antimicrobials, antibiotics, immunotherapeutics, vaginal microbicides and candidate vaccines/antigens to eliminate gonococcal colonization of the genital tract (Table 2). Demonstrable success of any of these compounds in the model is generally accepted as a requirement prior to in-human studies. Recently, the model has been used to demonstrate that the presence of a commensal *Neisseria* spp., *N. elongata*, in the mouse genital tract accelerated clearance of gonococci in a DNA-uptake-dependent manner, postulating that DNA could be a potential anti-gonococcal microbicide [128]. 

In addition to the oft-used intravaginal model of infection, the first model of upper genital tract gonococcal infection was recently reported [129]. In this study, transcervical infection of female mice during the dioestrus phase led to rapid gonococcal penetration into the tissues with production of high levels of inflammatory cytokines and neutrophil infiltration. By contrast, in the oestrus phase, mice showed little sign of inflammation or pathology following transcervical infection [129].

**Table 2:** Examples of compounds tested in the female mouse model of *Neisseria gonorrhoeae* genital tract infection.

Compounds Tested in the Female Mouse Model of *Neisseria gonorrhoeae* Genital Tract Infection		Reference
Antimicrobial	JSF-2659 [8-(6-fluoro-8-(methylamino)-2-((2-methylpyrimidin-5-yl)oxy)-9H-pyrimido[4,5-b]indol-4-yl)-2-oxa-8-azaspiro[4.5]decan-3-yl))methanol]	[130]
	Moenomycin (phosphoglycolipid targeting peptidoglycan glycosyltransferase)	[131]
	PTC-847 and PTC-672 active on ribonucleotide reductase	[132]
	Auranofin (gold-containing)	[133]
	Acetazolamide (carbonic anhydrase inhibitor)	[134]
	TP0480066, a topoisomerase inhibitor	[135]
	SCH-79797	[136]
	REDX05931 (tricyclic topoisomerase inhibitor)	[137]
	Resorufin pentyl ether (analogue of resazurin)	[138]
Antibiotic	MBX-4132 (acylaminooxadiazoles)	[139]
	Cephalosporins	[140]
	Cefixime and ceftriaxone	[141,142]
	Fluoroquinolone	[143]
Vaginal microbicide	Semen-derived enhancer of viral infection (SEVI)	[144]
	*Lactobacillus crispatus* producing hydrogen peroxide	[145]
	Porphyrin based compound with gallium	[146]
	CarraGuard, Ushercell, [poly]sodium 4-styrene sulfonate (T-PSS), PRO 2000, ACIDFORM, cellulose acetate phthalate (CAP), and BufferGel	[147]
Immunotherapeutic	Complement factor H-based immunotherapeutic	[148,149,150]
	C4BP-IgM protein	[151]
	Aminomethyl spectinomycins (semisynthetic analogues of spectinomycin)	[152]
	Anti-transforming growth factor β (TGF-β) antibody	[153]
Vaccine candidate	Meningococcal 4CmenB	[154,155]
	TMCP2 (peptide vaccine mimicking 2C7 oligosaccharide epitope in gonococcal LOS)	[156,157]
	Detoxified meningococcal outer membrane vesicle (dOMV) deficient in PorA, PorB, and RmpM	[158]
	MetQ lipoprotein formulated with CpG	[159]
	Hybrid transferrin binding protein B (from *Haemophilus parasuis*) with neisserial TbpA loop 10	[160]
	Recombinant human IgG1 chimeric variant of MAb 2C7 containing an E430G Fc modification (2C7_E430G)	[161,162]
	MAb 2C7	[163]
	2C7 epitope in multiple antigen peptide formulation	[164]
	Intranasal gonococcal OMV plus microencapsulated interleukin-12 (IL-12)	[165]
	Gonococcal OMVs plus microencapsulated IL-12	[166]
	Gonococcal OMV (intranasally administered)	[167]
	Gonococcal PI-B synthetic peptide	[168]
	rrPorB-Virus Replicon Particles prime and boost (rrPorB), both administered via footpad	[169]
	Opa-loop specific antibodies (passive protection trial)	[170]

- Studies with *N. meningitidis*.

In 1933, Miller induced peritonitis and sepsis in adult mice by inoculating bacteria intraperitoneally in the presence of hog mucin, with death occurring between 6 and 24 h [171], which was replicated in 1976 by Calver et al. using solutions of ferrous sulphate, iron sorbitol citrate or iron dextran [172]. Acute kidney injury can also be observed in mice infected intraperitoneally [173]. There is also an intraperitoneal mouse infection model modified by the addition of galactosamine to induce myelosuppression, which was used to study endotoxin lethality [174]. The virulence of other meningococcal molecules has also been demonstrated in the intraperitoneal mouse infection model, including sodC (encoding periplasmic copper- and zinc-cofactored superoxide dismutase) [175], phoP [176], transferrin-binding proteins [177], NMB1966, encoding a putative ABC transporter [178] and anti-aggregation factor NafA [179]. The model found early use for testing antibiotics [180].

Experimental meningococcal infection in neonatal mice as a model for mucosal invasion was reported by Salit et al. in 1984, in which meningococci were instilled intranasally in mice, which resulted in colonization and bacteraemia in 39% of the animals, and death in 4% of the animals with purulent leptomeningitis and ventriculitis [181]. Differences in virulence between disease and carrier strains is demonstrated by both the intraperitoneal and intranasal challenge models [182,183]. In addition, severity of meningococcal infection in neonatal mice can be increased by treating mice with iron dextran or human transferrin [184]. The neonatal intranasal infection model has shown that LOS immunotype and capsule are major virulence factors [185]. Furthermore, LOS, capsule and pilin [186] and the Outer Membrane Protein (OMP) NhhA autotransporter adhesin [187] have been shown to be necessary for colonization in an adult intranasal model of infection and that LOS is a critical inducer of inflammation during respiratory infection in mice [188]. A model of sequential influenza A virus–serogroup C meningococcal intranasal infection in adult mice also has been developed that reproduces the pathogenesis of human meningococcaemia with fatal sepsis and demonstrates the essential role of the capsule in virulence [189], and also that the inflammatory response is Nod-1-dependent [190]. Interestingly, feeding mice used in this viral–bacterial infection model with *Lactobacillus paracasei* CNCM I-1518 appeared to boost host defences against infection [191]. There is also a mouse model of meningococcal meningitis based on intracisternal infection of adult mice, which has been used to demonstrate the virulence of the L-glutamate transporter GltT protein [192] and of siaA [193]. Inhibition of matrix metalloproteinases has been shown to attenuate brain damage in this experimental meningitis model [194].

There are a few reports using knock-out mice to study the host response to meningococcal infection. Using the meningococcal sepsis model, Sjolinder et al. reported that Toll-like Receptor- deficient (TLR9(-/-))mice had reduced survival and higher levels of bacteraemia than wild-type mice [195]. In addition, the contribution of galectin-3 (a glycan-binding protein that binds to meningococci) to meningococcal bacteraemia was studied in galectin-3-deficient (Gal-3(-/-)) mice [196]. Bacteraemia was significantly reduced in knock-out mice, indicating that galectin-3 provides an advantage to meningococci during sepsis. The protective role of the Macrophage Scavenger Receptor A (SR-A) in sepsis was shown by the increased levels of bacteraemia, IL-6 cytokine and mortality in SR-A(-/-) animals intraperitoneally infected with meningococci [197].

As with the gonococcus, meningococci do not naturally colonize wild-type mice and this has seen the significant use of ‘humanized’ transgenic mouse models. One of the most oft used transgenic mouse is the human transgenic CD46 homozygous mouse (CD46+/+), which is susceptible to lethal sepsis and meningitis [198]. Intranasal rather than intraperitoneal challenge is more efficient at inducing systemic disease and expression of CD46 appears to accelerate inflammatory cytokine responses and induce large amounts of M1-type macrophages with enhanced surface expression of MHC II [199,200]. Use of the CD46 transgenic mouse model with bioluminescent imaging techniques allowed the imaging of the progression of meningococcal disease following injection of the bacteria directly into the bloodstream [201] and identified a major role for PilC1 adhesin for interaction with mucosal surfaces. Intranasal challenge of the same model suggested that one portal of entry of meningococci into the CSF was by direct passage of the bacteria from the nasopharynx to the meninges through the olfactory nerve system [202]. The model has also defined the roles of the glutamate dehydrogenase (GdhA) and Opa1800 [203], polynucleotide phosphorylase (PNPase) [204], App and MspA autotransporter [205], and PilU [206] PilE and PilQ [207] proteins in virulence.

Another transgenic mouse model expresses the human transferrin receptor, and intraperitoneal infection of these animals has allowed the process of meningococcal invasion of the blood, blood vessels, kidneys, heart, brain and skin to be studied [208]. Microarray transcriptional profiling of blood and brain samples from infected transgenic mice showed expression of genes encoding acute phase proteins, chemokines, cytokines and IL-1 receptor-associated kinase-3 (IRAK-3) [209]. The model has demonstrated the virulence role of the haemoglobin receptor, HmbR [210], differences in the virulence of serogroup W and Y bacteria [211] and the effects of corticosteroids on sepsis [212]. The CEACAM1 transgenic mouse model has highlighted the host specificity of meningococcal interactions and the essential role of Opa proteins in establishing intranasal and mucosal colonization [213].

**Figure 1:**
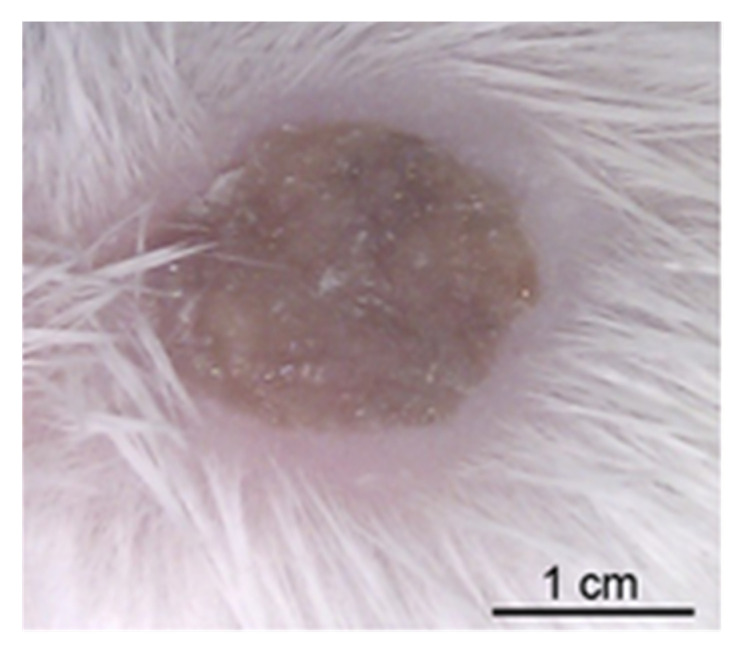
Human skin graft on mouse. The figure shows a patch of human skin xenografted onto the shaven torso of a SCID/Beige mouse. Reproduced from Melican, K.; Michea Veloso, P.; Martin, T.; Bruneval, P.; Duménil, G. Adhesion of Neisseria meningitidis to Dermal Vessels Leads to Local Vascular Damage and Purpura in a Humanized Mouse Model. *PLOS Pathog.* **2013**, *9*, e1003139. https://doi.org/10.1371/journal.ppat.1003139 [214].

And finally in this section, a discussion of the unique humanized mouse model in which human dermal microvessels were introduced into SCID/Beige mice by xenografting human skin (Figure 1) [214]. In this model, meningococci are injected intravenously, and the bacteria exclusively associate with the vessel endothelium in the skin graft. Infection was associated with a cytokine-dependent inflammatory response, the recruitment of neutrophils and a replication of purpura fulminans in the grafted skin, with haemostasis, thrombosis and vascular leakage. This is the first report of the classical purpura of meningococcal septicaemia mimicked in an animal model. Expression of pili was essential for meningococcal adhesion to dermal microvessels, and was the main determinant of vascular dysfunction. Subsequent studies with this skin graft model provide an increasingly deeper understanding of vascular colonization by meningococci and the inflammatory process that generates purpura fulminans [215,216]. Moreover, it demonstrated the key role of pilus retraction in promoting a sustained bacteraemia and lethality in the mouse [217], with meningococcal colonization of capillaries and arterioles creating an intravascular niche that limited the recruitment of neutrophils [218]. Moreover, the observation that both PilE and PilV promote adhesion to endothelial vessels in vivo [219] suggests that the pilus could be targeted as an anti-virulence strategy against systemic meningococcal disease [220].

#### 2.3.2. Mouse Models for Vaccine Research

Immunization of mice is useful for showing efficacy through the induction of serum bactericidal antibodies (SBA) that require complement to kill meningococci with the in vitro assay. The SBA assay serves as a correlate of protection for meningococcal vaccines. Vaccine antigens that have been examined include all the major capsular polysaccharides, OMVs and individual purified or recombinant surface antigens (Supplementary Table S1). Less frequently, the opsonophagocytic capacity of murine serum antibodies for meningococci has been reported. The mouse is often used to test vaccine efficacy by active protection against meningococcal infection and bacteraemia, for studying the immune-enhancing effects of adjuvants and for recording any vaccine–adjuvant toxicity (Supplementary Table S1).

Similarly, the mouse is used to examine the vaccine potential of gonococcal antigens, though there are considerably fewer reports of antigens capable of inducing serum bactericidal and/or opsonophagocytic responses (Supplementary Table S1). Active protection is generally tested using the female mouse model of gonococcal genital tract infection, described above, although a few other mouse models are noted. For example, a mouse model of gonococcal peritonitis induced by intraperitoneal injection of bacteria was described by Ito et al., who reported that it could be used to simulate human disease, in which an iron-rich environment produced by the addition of mucin and haemoglobin was necessary for the dissemination of infection [221]. Ito et al. demonstrated that vaccination with a heat-stable antigen protected mice against disseminated infection by stimulating neutrophil influx that cleared local infection. The heat-stable antigen was not related to LOS, and protection appeared to be independent of pili and bactericidal antibody. Moreover, vaccination could protect against heterologous infection with other gonococcal strains. Another mouse model used to study the immunizing properties of gonococcal LOS (R type) involved intraperitoneal immunization with antigen followed by intracerebral challenge with 10–20 LD50’s of gonococci [222]. Immunized mice were significantly protected against intracerebral challenge with different colony type 1 gonococcal isolates.

Mouse models have also been used to test the efficacy of antibacterial compounds. As an example, a model of meningococcal bacteraemia induced following intranasal infection with bacteria was used to demonstrate that administration of sodium tetraphenylborate could significantly reduce bacterial burden [223].

### 2.4. Guinea Pig

The guinea pig (*Cavia porcellus*) is one of the animals used in early *Neisseria* research in the 1970s that has been generally abandoned and we need not dwell on it in too much detail here. Advantages of the guinea pig, however, are that they are sensitive to infection, less variable in response, free of interfering micro-organisms, and more readily available to researchers than other animal models. It has been used in many vaccine studies to try to generate bactericidal antibodies to meningococcal and gonococcal antigens, and guinea pig serum has often been used as a source of exogenous complement for in vitro bactericidal assays. In addition to the general method of parenteral administration of vaccines, there is a guinea pig intranasal immunization model that has been used to examine the adjuvant properties of meningococcal OMP for *Brucella melitensis* LPS [224].

The guinea pig has been used to study gonococcal pathogenesis. In 1972, Arko reported the establishment of a subcutaneous chamber infection model [102]. In this model, plastic chambers/bottles of 2 mL volume are implanted surgically into the subcutis along the dorsolateral flank of the animal, and at least 2 weeks are allowed before gonococcal challenge to enable a connective-tissue-lined cavity to form for fluid accumulation and sampling [104]. The pathological findings in guinea pigs were identical to those described for the mouse above. By contrast, guinea pigs have had little use for studying meningococcal pathogenesis. In 1933, Branham and Lillie induced experimental meningitis in guinea pigs by intracisternal injection of boiled or filtered suspensions of meningococci [225]. An acute model of bacterial meningitis has been described in which animals are instilled intracranially with meningeal pathogens, including meningococci, and used to study the effects of antibiotics in clearing CSF infection(s) [226].

A summary of vaccine studies carried out in guinea pigs, and of infectivity experiments in the subcutaneous chamber model, is provided in Supplementary Table S2 with a supporting bibliography.

### 2.5. Rabbit

Much of the published literature on the use of rabbits in *Neisseria* research just cites the use of baby ‘rabbit’ complement as an exogenous complement source in validated bactericidal assays for animal and human sera raised to serogroup A, C, W and Y meningococcal vaccines. There are studies that use rabbits to generate bactericidal antibodies to meningococcal and gonococcal experimental antigens/vaccines, but these are fewer in number than studies that use mice (Supplementary Table S3). Rabbits have been used far less frequently to generate opsonophagocytic antibodies to vaccine antigens [227], and their utility for testing endotoxicity pyrogenicity has largely been superseded by the availability of in vitro assays. A *N. lactamica* OMV vaccine has been reported to induce opsonophagocytic, but not bactericidal, antibodies in rabbits [228].

The rabbit has been used occasionally for pathogenesis studies. In 1948, Miller described a model of intraocular infection in which gonococci were observed to invade and multiply in the lens and ciliary bodies in the eyes [229]. Experimental infection of subcutaneously implanted chambers with gonococci was established in the 1970s [102], and intravenous injection of gonococci could induce endocarditis in rabbits with transaortic valve catheters [230]. Intra-articular injection of virulent gonococci produced an arthritis characterized by an acute, purulent synovitis with PMNL infiltration [231]. An intradermal injection model has been used to demonstrate the ability of gonococcal LOS to induce skin lesions [232], and the intrauterine injection of gonococci in a model of PID has been shown to cause pelvic adhesions and inflammation [233,234]. There are two models that use rabbit corneas to study gonococcal colonization: in one model, functional but uncharacterised human membrane gonococcal receptors that were identified on the surface of cultured U937 lymphoma cells after infection with gonococci were transferred directly by electrofusion to rabbit corneas in vitro [235]; in the other model, the same receptors were transferred to rabbit corneas in vivo [236]. Notably, these histologically modified corneas could be infected in vivo with live gonococci [236].

In the case of meningococci, there is a well-established model of endotoxic meningococcal shock [237,238] and of meningitis, the latter induced following intracisternal injection of meningococcal LOS [239].

### 2.6. Rat

#### 2.6.1. Studies with *N. gonorrhoeae*

The rat has been little used in gonococcal research, again principally due to the human-restricted nature of the pathogen. Rats are resistant to experimental intraperitoneal infection with gonococci [240] and will not produce intra-abdominal abscesses alone, but only when combined with facultative or anaerobic bacteria [241]. However, rats with subcutaneously implanted chambers can be inoculated with gonococci, which leads to infiltration of gonococci into the tissue and blood vessels surrounding the infected chamber [102]. The presence of human complement C1q increases the virulence of gonococci in rat pups [242], and enables the development of disseminated infection and PID in Sprague–Dawley rats that were infected on day 20 of pregnancy by intraperitoneal inoculation with gonococci. Notably, gonococcal infection could be passed from the pregnant rat to the foetus, resulting in foetal death [243]. This C1q-dependent experimental model of gonococcal infection of newborn rats demonstrated that the serum-resistant genotype sac-4 conferred C1q-dependent virulence, which is uniquely characteristic of PID [244]. In addition, the disseminated arthropathic properties of gonococcal peptidoglycan fragments were demonstrated in adult rats [245], and LOS could induce a recurrence of arthritis in rat joints previously injured by peptidoglycan–polysaccharide fragments [246]. Moreover, injection of rats with purified, soluble, macromolecular, extensively O-acetylated peptidoglycan fragments decreased overnight food consumption in male Lewis rats [247].

Little gonococcal vaccine-related research has been carried out in rats. In the literature, we find one report of rats developing a mucosa-associated IgA response to gonococcal PI protein intra-intestinally, which was augmented by the inclusion of muramyl dipeptide adjuvant [248].

#### 2.6.2. Studies with *N. meningitidis*

The neonatal (infant) rat model of meningococcal infection is a well characterized model that has been used extensively for pathogenesis studies and for active and passive immunization studies. Nasal instillation of bacteria is used to establish infection, and the model has increased our understanding of the metabolic processes and virulence properties of meningococci during bacteraemia and meningitis. Thus, the infant rat model has shown that haemoglobin utilization [249], FNR-regulated genes required for anaerobic metabolism [250], luxS (involved in the biosynthesis of a quorum sensing molecule, autoinducer-2) [251], HexR expression (which accounts for some glucose-responsive regulation) [252] and the involvement of gdhA, encoding the NADP-specific l-glutamate dehydrogenase [253] and the RNA chaperone Hfq [254], are required for systemic meningococcal infection. Analysis of a library of 2850 insertional mutants of meningococci for their capacity to cause systemic infection in the infant rat model identified 73 genes that were essential for bacteraemia [255]. In addition, sRNAs have been reported to influence meningococcal bacteraemia [256]. With respect to surface-exposed virulence factors, the sialic acids of both the capsule and the sialylated LOS, the expression of PorB2 and the protein components of the Bexsero vaccine, including GNA2091, are necessary for meningococcal virulence in the infant rat [257,258,259,260]. The only other *Neisseria* spp. to have been used in rats is *N. sicca¸* for which there is a report of induced thyropathy following inoculation of the commensal organism into the apex of lingual V [261].

The neonatal (infant) rat has been used extensively for meningococcal vaccine research. In 1986, Salit first reported that intranasal challenge of 5-day-old neonatal rats with meningococci resulted in 16% of rats developing bacteraemia [262]. The infant rat can also be infected intraperitoneally to induce peritonitis, bacteraemia and meningitis [263]. The passive infant rat protection model is most used to test the ability of murine monoclonal antibodies and polyclonal mouse, rabbit and/or human sera raised to test vaccine antigens, to protect the animals from infection with live meningococci. Moreover, all these antibodies have demonstrated complement-mediated bactericidal activity in vitro, with only one exception, to our knowledge; namely, that of mouse antibodies to GNA2132 that conferred protection against bacteraemia in the absence of bactericidal activity [264]. The list of meningococcal vaccine antigens that induce antibodies passively protective in the infant rat are listed in Supplementary Table S4. As with mice, efforts have also been made to humanize rats, but they have seen limited use. Intraperitoneal infection of human factor H transgenic rats with meningococci leads to enhanced bacteraemia [265], and a mouse monoclonal to fHbp has been shown to augment passive protection against meningococcal bacteraemia in the same transgenic animals [266].

Rats have been used for testing immuno-therapeutics, e.g., a fusion protein comprising factor H domains 6 and 7 and human IgG1 Fc reduced bacteraemia in meningococcal-challenged rats [267], and TNF antibody has been shown to protect infant rats against lethal meningococcaemia [112]. More commonly, they are valuable for studying the pharmokinetics of anti-gonococcal antibiotics and for toxicity/reactogenicity studies of gonococcal and meningococcal antigens and vaccines [102,103,104,105].

### 2.7. Horse

To our knowledge, the horse has been used only to produce hyperimmune serum for meningococcal research. Specifically, hyperimmune serum from a single horse (number 46) that had been immunized with a killed serogroup B (strain B-11) meningococcal vaccine has been used as a reagent for primary isolation media, large-scale serogrouping and immunochemical studies on capsular polysaccharides [268].

### 2.8. Chicken Embryo Model

The use of chicken embryos as an animal model for gonorrhoea dates to the 1970s with the papers from Finklestein and colleagues [269,270,271]. The model has shown some use in demonstrating passive protection by immune sera against lethal infection and for investigating gonococcal virulence, e.g., the role of cardinal components such as LOS, pili and the opacity proteins, and the dependence on iron [270,272,273,274]. Different routes of inoculation can be used, including intravenous routes, and routes through the yolk sac and chorioallantoic membrane [275]. Diena et al., were able to show that immunity to gonococci could be transferred from vaccinated hens to embryos [276]; for example, embryonated eggs obtained from hens immunized intravenously with LOS, were protected against lethal gonococcal challenge [222].

The model has also been used in some early vaccine-related studies. For example, Robertson showed that rabbit antisera to whole OM and pilus could protect chick embryos challenged with a lethal dose of gonococci, whereas the effect with LOS itself was less pronounced and antisera raised against individual OMPs were non-protective [277]. The model has also proved useful for studying meningococcal vaccine antigens; Ashton et al. immunized hens with meningococcal serotype 2 OMP (SP-2) and showed that embryos from these animals were protected against challenge with up to 10,000 LD50 doses of the homologous strain, with significant protection also shown against challenge with heterologous strains [278].

Despite the flurry of activity around the hen embryo model for a decade or so, it is no longer used. More recently, however, a study reported the isolation of *Neisseria* spp. from eggs of the Greater white-fronted goose (*Anser albifrons*) on the Arctic Coastal Plain of Alaska, where mortality of developing embryos had been observed. These *Neisseria* spp. most closely resembled *N. animalis* and *N. canis* and isolates were used to infect developing chicken eggs, with mortality rates reaching 100% by day 7 post-infection, depending on the inoculation dose [279]. Thus, bacterial infections can result in embryo mortality in bird populations.

### 2.9. Pig (Sus domesticus)

There is an experimental porcine meningococcal sepsis model for studying the pathophysiology of endotoxic shock [280]. The model has been used to study the in vivo responses of a large animal to LOS and non-LOS structures after the infusion intravenously of LPS-replete and LPS-deficient meningococci [281]. Such studies confirmed the role of LOS in endotoxic shock and that non-LOS structures can induce cardiovascular and hematologic changes, but with higher doses required. The porcine model mimics cardinal aspects of human sepsis, including the production of inflammatory mediators and organ inflammation [282,283] and microvascular findings in tongue and skin [284]. It also offers an opportunity for testing anti-sepsis therapies; for example, the bradykinin receptor antagonist icatibant has been tested, but without success in preventing meningococcal-induced oedema, shock and inflammation in the pig [285].

There are *Neisseriaceae* and related species that can infect pigs, but the pig has not been used for in vivo gonococcal research, which contrasts with their use for studying *Chlamydia* pathogenesis [286]. An ex vivo porcine vaginal mucosa model has been reported but not used extensively [287].

### 2.10. Zebrafish (Danio rerio)

**Figure 2:**
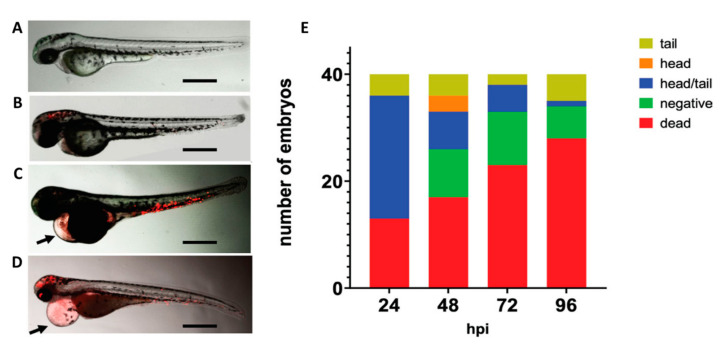
Fluorescence microscopy of zebrafish embryos infected with mCherry expressing *N. meningitidis* strain H44/76. Panels (**A**–**D**) show zebrafish embryos infected with red fluorescent meningococci, with (**A**) no meningococci visible, (**B**) meningococci in the head, (**C**) meningococci in the tail and (**D**) meningococci throughout the body. Arrow denotes pericardial oedema. (**E**) The distribution of meningococci in infected zebrafish embryos. © 2022 Schipper, Preusting, van Sorge, Pannekoek and van der Ende. Reproduced from Schipper, K.; Preusting, L.C.; van Sorge, N.M.; Pannekoek, Y.; van der Ende, A. Meningococcal virulence in zebrafish embryos depends on capsule polysaccharide structure. *Front. Cell. Infect. Microbiol.* **2022**, *12*, 1020201. https://doi.org/10.3389/fcimb.2022.1020201 [290].

The zebrafish (Phylum *Chordata*, Class *Actinopterygii*, Order *Cypriniformesis*) has become a widely used vertebrate model in biological research and for studying infection with many human and animal bacterial pathogens [288], including some reported use with meningococci. The zebrafish embryo innate immune system shares similarities with the mammalian system, including the expression of TLRs, and the presence of phagocytic neutrophils and macrophages [289]. A key attribute of the embryo is its transparency, which makes it an interesting model with which to study bacteria–host interactions in real time using fluorescent tools. Recently, the zebrafish embryo was developed as a meningococcal infection model to study pathogenesis and, in particular, the effect of CPS structure on virulence [290]. In this study, CPS meningococci were injected in the caudal vein of the zebrafish embryo and shown to replicate and cause a dose-dependent lethal systemic infection, with a characteristic pericardial oedema (Figure 2). By contrast, zebrafish embryos could clear infection with non-CPS meningococci and were not killed. The study also demonstrated that the capability of meningococci to kill zebrafish embryos and deplete neutrophils was correlated with the number of carbons per CPS repeat unit during its biosynthesis [290]. The model could see further use in studying bacteria–host interactions, with a focus on pathogen interactions with the innate immune system and with high-resolution imaging of bacterial interactions with cellular barriers, e.g., the blood–cerebrospinal fluid/brain barriers.

## 3. Invertebrate Animal Models

In contrast with the wide variety of vertebrate animal models used in *Neisseria* research, the use of invertebrates has been under-explored. This is not entirely unexpected, as invertebrate animals are unlikely to express the precise pattern recognition receptors (PRRs) that can recognise *Neisseria* pathogen associated molecular patterns (PAMPs) or receptors binding specific bacterial adhesins. Despite this, invertebrates do share some similarities with mammals in their innate immune systems.

### 3.1. Galleria Mellonella Larvae

**Figure 3:**
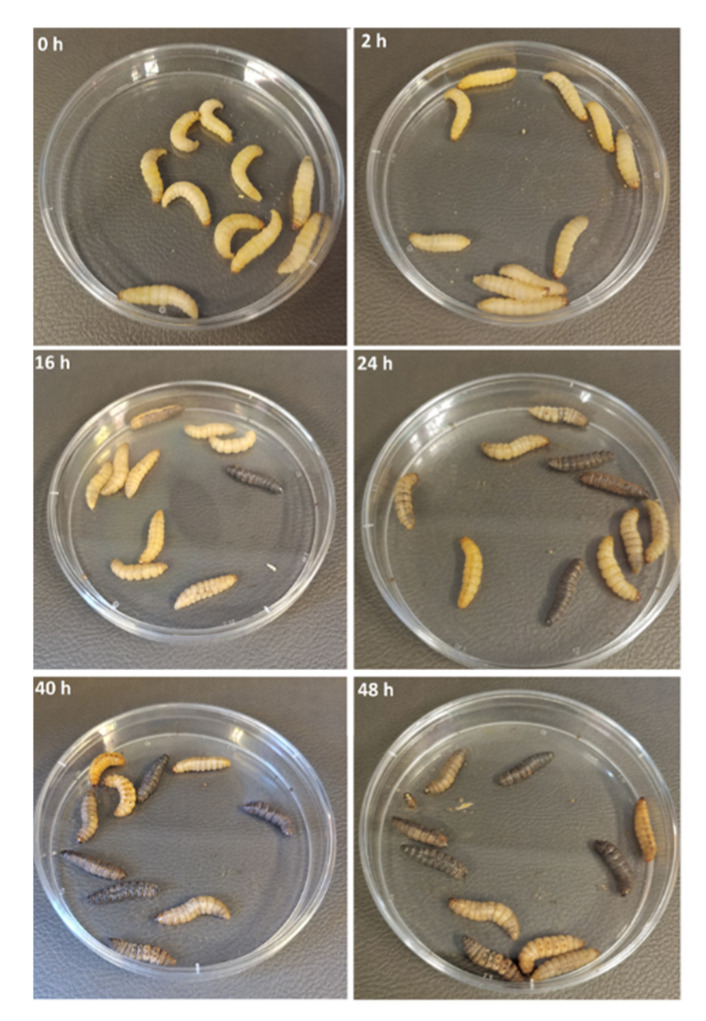
Images of larvae infected with gonococci. The images show the development of melanisation, i.e., the observed blackening of the larval body as a marker of larval death with time. © Dijokaite, Humbert, Borkowski, La Ragione, Christodoulides. Reproduced from Dijokaite, A.; Humbert, M.V.; Borkowski, E.; La Ragione, R.M.; Christodoulides, M. Establishing an invertebrate *Galleria mellonella* greater wax moth larval model of *Neisseria gonorrhoeae* infection. *Virulence* **2021**, 12, 1900–1920, https://doi.org/10.1080/21505594.2021.1950269 [291].

Recently, the utility of larvae of the greater wax moth *Galleria mellonella* (Subfamily *Galleriinae*, Family *Pyralidae*, Order *Lepidoptera*) has been examined as an in vivo model of *Neisseria* infection [291]. There is extensive literature describing the use of *G. mellonella* larvae to study infection by many bacteria, fungi and parasites, and its use as a model for testing new antibacterial, antifungal and antiparasite strategies and novel drugs and antibiotics [291]. Infection was carried out via injection of live *Neisseria* into the last left pro-leg of the larvae, although the oral route is available and has been used for periodontal pathogens [292], although not assessed with the *Neisseriae*. A threshold of ~10^6^–10^7^ gonococci/larva was needed to kill >50% of larvae, with increased toxicity correlating with reduced health index scores and pronounced histopathological changes such as increases in the total lesion grade, melanized nodules, hemocyte reaction and multifocal adipose body degeneration (Figure 3). Infection with meningococci and *N. lactamica* had similar outcomes, and all *Neisseria* spp. were significantly less toxic than *P. aeruginosa*. This is probably related to the lack of cytotoxic virulence factors. Indeed, larval death was independent of the expression of pilus or Opa protein or LOS sialylation, although it could be induced with physiologically excessive amounts of LOS-replete OM. Larval death required live bacteria and interestingly could be enhanced significantly if larval haemocytes were depleted. The larval innate immune system does share some similarities with the mammalian innate immune system, with haemocytes functioning in phagocytosis, nodulation and encapsulation [293]. Larvae can also undergo melanisation, express a prophenolaxidase-activating system (Pro-PO-AS) and can synthesise small antimicrobial peptides and lysozyme. The model did prove useful for testing the anti-gonococcal properties of antibiotics and novel antimicrobials; for example, ceftriaxone could protect larvae from systemic infection with different gonococcal isolates, but not azithromycin or the fatty acid monocaprin or ligand-coated silver nanoclusters.

However, the model has some clear limitations. Such larvae are not a natural target for infection with *Neisseria* spp., as shown by the lack of bacterial replication within the larvae over time. The most probable explanation is that the larvae do not express the specific receptors that recognise gonococcal ligands, e.g., the absence of pilus-binding receptors or CEACAM receptors. Interestingly, heparin sulphate proteoglycan (HSPG) molecules and mucopolysaccharides are produced by insects, and, speculatively, may play a role as receptors for *Neisseria* Opa proteins. The exact mechanism by which *Neisseria* kill larvae is not known, but may involve toxic components such as the release of peptidoglycan fragments or other potential toxins [294]. Thus, the model may not prove useful for studying *Neisseria* adhesion and invasion events in vivo but may be valuable for assessing putative toxins/virulence factors, without resorting to cell cultures and mammalian models, and for testing novel antimicrobials.

### 3.2. Can Neisseria Infect Other Insects?

The literature describes the identification of *Neisseria* in other insects, most often from metagenetic analyses of 16S rRNA genes in insect microbiota (Table 3), although only at the genus level, and the exact species is not provided. Intriguingly, there is one paper that describes the Australian bushfly as a possible vector of gonococcal conjunctivitis, although as the author notes in his hypothesis, the gonococcus is likely to be an accidental passenger [295]. Yet, a previous report of the association of high densities of flies with a community outbreak of gonococcal conjunctivitis in Ethiopia, where genital transmission could not explain the outbreak, suggested that flies and a lack of personal hygiene were important in person-to-person transmission [296]. The identification of *Neisseriaceae*-related bacteria as a dominant obligate symbiont in species of louse [297] can be postulated as a potential means of transmission of bacteria causing sexually transmitted diseases. For example, Pierzchalski et al. reported that *Phthirus pubis* (pubic louse) infestation was predictive of a concurrent *Chlamydia trachomatis* infection in a population of sexually active adolescents at a juvenile detention centre, and gonococcal infection was reported as higher in index cases (18%) than in controls (9%) [298]. In their discussion of the literature, the authors suggested that adolescents with pubic lice infestation should be screened for sexually transmitted diseases, including chlamydia and gonorrhoea, but did not discuss the louse as a possible transmission vector. In our opinion, the identification of *Neisseria* 16sRNA gene signatures, but not a definitive culture, does not definitely suggest that these insects can be developed as other invertebrate models of infection.

## 4. Can the 3Rs Be Applied to *Neisseria* Research?

‘Replacement, Reduction, Refinement’(3Rs) is a trio of principles first suggested in 1959 by WMS Russell and RL Burch [303]. The goal of the 3Rs is to find alternatives to animal models (replacement), to maximise the use of data obtained from fewer animals (reduction), and to adopt housing and husbandry protocols that minimise animal suffering that can affect homeostasis during experiments (refinement). These principles serve to improve the quality of preclinical research and of animal welfare when the use of animals is unavoidable, and they are given significant weight by agencies funding biomedical research [304]. The use of animal models in *Neisseria* research is being constantly questioned both ethically and biologically, given the human-restricted nature of the pathogens. Thus, all *Neisseria* animal models have their limitations, and it can be argued that the use of alternative, non-animal-based approaches could provide more flexibility, reproducibility and cost-efficiency savings. To apply the 3Rs, several models have been proposed and are discussed briefly below.

### In Vitro 3D Models and Organoids

Over the past fifty years, most research studying *Neisseria* colonization and invasion has been carried out using simple monolayer cultures of primary cells and immortalized cells and using explants of primary tissues. These are reviewed extensively by Heydarian et al. [305], and not all of these cell lines have come from relevant tissue; this raises questions about the validity of extrapolating from in vitro data to in vivo biology. The numbers of papers using cell culture models to study *Neisseria*–host cell interactions are too numerous to detail. However, recent efforts have been dedicated to transitioning from simple monolayer cell culture approaches to more complex cell cultures, including three-dimensional (3D) approaches and ex vivo tissue culture or organoids, which are proposed to provide a more realistic physiological environment to simulate the site of infection of the original hosts.

Three-dimensional cultivated cells are spatially organized cells that grow and interact with each other and with the surrounding extracellular scaffold in three dimensions, and are suggested to mimic the in vivo cell configuration. Organoids are tiny, self-organized 3D masses of tissue that are obtained from stem cells. Such cultures can be fashioned to replicate much of the complexity of an organ, or to express particular aspects of it, e.g., the production of only certain kinds of cells [306]. They are believed to respond better to physiological signals than conventional monolayer cultures, and can provide new insights into the pathophysiology of bacterial infection by addressing questions, for example, on the role of the microbiota, cell structures, extracellular matrix (ECM) and tissue exfoliation. Moreover, using natural-based scaffolds, such as decellularized ECM derived from different human tissues, produces 3D models with optimal mechanical and biochemical properties and immunological responses like those of the host. To produce acellular ECM scaffolds, the cellular components of organs are removed and certain ECM proteins retained, e.g., collagen, fibronectin and laminin. In the case of porcine small intestinal scaffold (SIS), a network with interconnected pores offers a great support for cell growth and differentiation [307,308,309]. In addition, more advanced techniques have been introduced to culture cells under conditions equivalent to an in vivo microenvironment, such as perfusion-based bioreactor techniques that have been used to study gonococcal infection under dynamic culture conditions, which also provides an opportunity to mimic specific immunological responses that are impossible with static culture [310]. New approaches also include the fabrication of 3D ‘artificial tissues and organs’ from biological and biocompatible materials made using computer-controlled techniques in a process called ‘3D bioprinting’. These artificial organs and tissues are engineered to closely mimic the native organs using three types of materials—(i) building materials or hydrogels such as collagen [311], hydrogel from sodium alginate [312], agarose [313] and novogels [314]; (ii) supportive materials such as carbohydrate glass (glucose, sucrose, dextrane) [315], pluronic F127, gelatin [316] and hydrophobic high-density perfluorotributylamine [317] and (iii) cells or tissues derived from humans or animals. Sophisticated software and printing systems such as nozzle-based, laser-based and bioprinter-based systems can be used to recreate the structure and behaviour of the organ and provide an environment for all of the cell types present within an organ. These approaches are being used to develop 3D printed experimental models of infectious diseases, inflammatory disorders or therapeutic replacements of human tissues. For example, they have been used extensively to build neurovascular units resembling the human brain and vascular system in combination with microfluidic chips to produce models that can be perfused. However, bioprinting faces major challenges to exactly match all material and mechanical requirements of the printed materials for different biological and physicochemical needs [315,318,319,320].

The normal sites of colonization of gonococcus and meningococcus are the urogenital tract and the respiratory tract, respectively, although both pathogens can colonize other mucosal body sites [7]. Different animal-derived 3D models and organ cultures such as rabbit oviducts [321], guinea pig urogenital tract [322] and a 3D artificial scaffold model of the bovine cornea [323] have been used for studying *Neisseria* pathogenesis and host immune responses. However, for human-restricted pathogens such as *N. gonorrhoeae* and *N. meningitidis*, the use of animal-derived cells might not be wholly relevant. Human-derived cell and organ cultures provide striking features of specificity and selectivity and give better information about gonococcal and meningococcal colonization, adhesion and invasion during infection [324]. Several organoids, ex-vivo and 3D cell culture human-derived models relevant to gonococcal and meningococcal infections have been established to include epithelial, endothelial, stromal and immune cells, and key models are summarised briefly in Supplementary Table S5. Recently, an in vitro hollow fibre infection model (https://hollowfiberinfectionmodel.com/ “URL accessed on 30 May, 2023”) has been used to simulate gonococcal infections and study the pharmacokinetic and pharmacodynamic properties of new antimicrobials, and obviate the need for animal models of toxicity testing [325].

## 5. Prospectus and Conclusions

The use of vertebrate and invertebrate animal models has been indispensable in helping us understand the mechanisms of *Neisseria* interactions with the host and for the development and testing of vaccines and antibiotics/antimicrobials. Many of these models mimic aspects of *Neisseria* infection in humans, and these are summarized in Table 4. In addition, the researcher is provided with an explanation of the potential uses of each model to aid their choice and whether they are in use today; the latter is important, as many of these models require training from experienced users and for some, e.g., the human challenge models, a high degree of collaboration is needed. All these models have their advantages and disadvantages, but several of them are still likely to be used over the next decade to address the significant gaps in *Neisseria* research (Table 4). Notably, the female mouse intravaginal infection model and the Ng CHIM will help to address the biggest gaps, which are the lack of an effective gonococcal vaccine and of new antimicrobials for treating local and systemic infections. Wild-type and transgenic mouse models will continue to be useful for studying the finer details of gonococcal and, to a lesser extent meningococcal pathogenicity, and new insights into *Neisseria*–host interactions could inform new intervention strategies, e.g., for limiting colonization, enhancing innate immune responses and reversing immunosuppressive responses.

Without considerable further research to correlate responses in vitro with in vivo, the complete replacement of animals with new in vitro models and organoids is a matter for debate. And finally, and the subject of a review in itself, the use of computational methods, modelling and the plethora of emerging artificial intelligence and deep/machine-learning algorithms offers a different opportunity for the 3Rs by enabling the study of host–pathogen interactions in silico at the molecular and genetic levels. For example, computer-aided methods can be used to (i) investigate the structure and function of pathogenic factors, (ii) determine antigen variation and diversity, (iii) map T- and B-cell epitopes for designing new subunit vaccines, (iv) discover new antimicrobials through artificial intelligence screening of compound libraries, (v) study a variety of omics and (vi) develop sequence-based diagnostics. All these approaches have broad applicability to controlling infectious diseases of all aetiologies.

## Figures and Tables

**Table 1:** Studies reporting the use of rhesus macaque monkeys in *Neisseria* vaccine-related research.

Rhesus Macaque Monkeys Used in *Neisseria* Vaccine-Related Research		Reference
Vaccine studies	Immunization with meningococcal OMV vaccine with overexpressed mutant factor H binding protein (infant RMs)	[75]
	Trivalent native OMVs derived from three genetically modified serogroup B meningococci (infant RMs)	[76]
	MenB-4C immunization (infant RMs)	[68,77,78]
	MenB capsular polysaccharide–protein conjugate vaccines (juvenile RMs)	[79,80]
	Chemically modified *Escherichia coli* K1 N-propionylated polysialic acid coupled to purified recombinant rPorB	[81]
Adjuvant studies	*Bacillus anthracis* polyglutamic acid capsule covalently conjugated to the *N. meningitidis* serotype B OM protein complex	[82,83]
	Recombinant modified human vascular endothelial growth factor (VEGF) combined with proteoliposomes of meningococcal OM	[84]
	*Plasmodium falciparum* circumsporozoite protein NANP_6_ peptide conjugated to *N. meningitidis* serotype B OM protein (in adult RM)	[85]
	Domain III of the dengue 1 virus fused to meningococcal P64k protein (in adult RM)	[86]
	*P. falciparum* recombinant Pfs25H transmission-blocking protein conjugated to *N. meningitidis* serotype B OM protein complex	[87]
	Influenza virus A M2 peptide-*N. meningitidis* serotype B OM protein complex conjugate	[88]
	Pneumococcal capsular polysaccharide (Ps)–*N. meningitidis* serotype B OM protein complex conjugate	[89,90]
	*Haemophilus influenzae* type B polysaccharide conjugated to meningococcal Class 2 porin (in infant RMs)	[91]
	*H. influenzae* type B polysaccharide–meningococcal OM protein complex conjugate	[92]

**Table 3:** Identification of *Neisseria* spp. in insects.

Insect	Species	Reference
*Musca vetustissima* Walker (Australian bushfly)	*N. gonorrhoeae*	[295]
*Apis mellifera capensis* and *Apis mellifera scutellata* (worker honeybees)	Sequences related to *Simonsiella/Neisseria* (amongst others)	[299]
*Musca domestica* (housefly)	*Neisseria* in pupae; denaturing gradient gel electrophoresis analysis identifies *N. gonorrhoeae* NCTC83785 (X07714) and *Neisseria* sp.93S1 (EU370420)	[300]
*Ruspolia differens* (edible grasshopper)	*Neisseria* (operational taxonomic unit)	[301]
*Platypus cylindrus* Fab. (Ambrosia Beetle)	*Neisseria* spp.	[302]
*Hoplopleura acanthopus*, *Polyplax* microbiomes (louse)	*Neisseriaceae*-related bacterium (dominant obligate symbiont)	[297]

**Table 4:** Vertebrate animal models mimic aspects of human infection and many still have significant potential for *Neisseria* research.

Pathogen	Animal	Aspect of Human Infection Mimicked by the Animal	Potential of Model	In Use Today
*Neisseria gonorrhoeae*	Ng CHIM	Natural urethral male challenge model; mimics clinical symptoms of pus urethritis	Essential for studying transmission, inflammation, re-infection, changing bacterial population dynamics, antigen variation, immunity and immunosuppression and vaccine trials.	Yes
	Chimpanzee	Mimics clinical symptoms of pus urethritis and pharyngitis	Utility for immunological and vaccine research is limited due to their high cost Ethical issues around their use Outbred animals with more variable individual immune responses	No
	Rhesus macaque	Colonization of nasal cavity and epithelium covering the cribiform plate, and persistence	Potential for studying commensal Neisseria colonization, transmission, long-term persistence, horizontal gene transfer asymptomatic carriage by pathogenic Neisseria preclinical testing of vaccine antigens persistence of antibiotic resistant determinants in a population	Limited
	Wild-type mouse	Used to mimic histopathology of DGI, neutrophil infiltration, pneumonia	Most models have fallen into disuse as wild-type mice are not naturally infected with gonococci, and aspects of histopathology are dependent on route of infection and iron supplementation.	No
	‘Humanized’ mouse	Aspects of the human inflammatory and cellular response and neutrophil infiltration	Transgenic mice overcome the resistance to natural colonization by gonococci and are useful for studying the cellular biology of infection.	Yes
	Mouse model of intravaginal infection	Hormone-influenced colonization of the lower genital tract mucosa	Important for studying gonococcal virulence factor interactions with host cell receptors induced inflammation and clearance evasion of innate immunity and suppression of adaptive immunity adaptation to host environment mechanisms of antibiotic resistance Key model for preclinical testing of vaccines, by active and passive immunization, and of therapeutics	Yes
	Mouse model of upper genital tract infection	Mimics tissue invasion, inflammatory cytokine production and neutrophil infiltration	Useful for studying aspects of disseminated infection, including endometritis, salpingitis (Fallopian tube) and peritonitis, and associated organ damage and potentially infertility.	Yes
	Guinea pig	Pathological findings as with mouse	Guinea pigs have the advantages of sensitivity to infection, less variable in response, free of interfering micro-organisms, ready availability.	No
	Rabbit	Conjunctivitis, endocarditis, arthritis, pelvic inflammatory disease	Useful for studying aspects of gonococcal pathogenesis and host response, but largely superseded by small rodent models, which can provide animal numbers for better reproducibility.	No
	Rat	Disseminated infection and pelvic inflammatory disease; arthritis	Useful for studying induced pelvic inflammatory disease processes.	No
	Chicken embryo	Lethality	Useful for studying gonococcal virulence factors, immunity, and for passive protection studies of anti-gonococcal sera.	No
*Neisseria meningitidis*	Wild-type adult mouse	Peritonitis, bacteraemia, sepsis, meningitis, kidney damage	Useful for studying aspects of systemic meningococcal disease, pathogen virulence factors and host innate immune responses. Vaccine studies—active and passive protection against infection and generation of bactericidal and/or opsonophagocytic antibodies.	Yes
	Knock-out mice	Peritonitis, bacteraemia, sepsis, meningitis, kidney damage	Useful for studying aspects of systemic meningococcal disease host innate immune responses. Vaccine studies—active and passive protection against infection and generation of bactericidal and/or opsonophagocytic antibodies.	Yes
	Neonatal mouse	Intranasal infection leading to colonization, bacteraemia, meningitis and ventriculitis	Useful for studying mucosal invasion and pathogen virulence factors and host innate immune responses.	Yes
	Adult mouse human skin xenograft	Replicates purpura fulminans	Useful for deeper understanding of vascular colonization by meningococci and the inflammatory process that generates purpura fulminans, and virulence factors involved.	Limited
	Guinea pig	Acute meningitis	As above, but more generally useful for vaccine studies.	No
	Rabbit	Meningococcal shock and meningitis	Useful for studying systemic meningococcal disease, but largely superseded by small rodent models.	No
	Infant rat	Bacteraemia, peritonitis, meningitis	Used extensively for pathogenesis studies and for active and passive immunization studies.	Yes
	Pig	Sepsis	Established model for studying the pathophysiology of endotoxic shock and anti-sepsis therapies.	Limited
	Zebrafish	Innate immune responses to infection	Not generally used, yet. Potential for studying bacteria–host interactions, pathogen interactions with the innate immune system, high-resolution imaging of bacterial interactions with cellular barriers, e.g., the blood–cerebrospinal fluid/brain barriers.	Limited
*Neisseria lactamica*	Nlac CHIM	*Neisseria* colonization of nasopharynges	Useful for studying *Neisseria* colonization and carriage, host mucosal immunity, vaccine delivery with genetically modified bacteria.	Yes

## Supplementary Materials

The following supporting information can be downloaded at: https://www.mdpi.com/article/10.3390/pathogens12060782/s1, Table S1: Use of the mouse for testing Neisseria vaccine efficacy, through the generation of bactericidal antibodies, opsonophagocytic antibodies and active protection against infection; Table S2: The guinea pig in Neisseria research; Table S3: Use of the rabbit for testing Neisseria vaccine efficacy, through the generation of bactericidal antibodies; Table S4: The neonatal (infant) rat model of passive protection used to test the ability of antibodies generated to meningococcal antigens to protect against infection with live meningococci; Table S5: Human-derived cell culture-based models for studying Neisseria pathogenesis.
