# Supplementary material for: Vertebrate and Invertebrate Animal and New In Vitro Models for Studying Neisseria Biology
Source: Pathogens. 2023 May 30;12(6):782. doi: 10.3390/pathogens12060782 (PMC10301118; doi:10.3390/pathogens12060782)
Supplement: Supplementary file 1 [file pathogens-12-00782-s001.zip › pathogens-2346025-supplementary.pdf]

Supplementary Tables

Supplementary Table S1. Use of the mouse for testing *Neisseria* vaccine efficacy, through the generation of bactericidal antibodies, opsonophagocytic antibodies and active protection against infection

**1. Generation of serum bactericidal antibodies against meningococci**	
**Antigen**	**References**
Outer membrane protein (OMP) or OMP plus group C CPS	[1]
Groups A, B, and C CPS-tetanus toxoid conjugates	[2]
Serogroup B CPS	[3]
CPS-OMP complexes serogroup C, serotype 2a; and tetanus toxoid conjugates	[4,5]
Group B serotype 2b protein vaccine	[6]
Passive immunization with group B serotype 2b-specific monoclonal antibody	[7]
Class 1/3 OMP vaccine against group B, type 15, subtype 16 meningococci	[8]
28 kDa OMP	[9]
H.8 (Lip)	[10,11]
Monoclonal antibodies against the 70kDa iron-regulated protein	[12]
Outer membrane complexes (OMCs) containing multiple Class I proteins	[13]
Class I OMP/PorA, recombinant or purified	[14-32]
PorA peptides	[33-39]
IgG2a mAbs for group B CPS	[40]
LPS-derived oligosaccharide-protein conjugates	[41,42]
Transferrin-binding proteins (Tbps)	[43-49]
98-kilodalton iron-regulated OMP (TbpA)	[50]
70 kDa iron-regulated protein FrpB, FetA	[51,52]
Class 5 protein/Opc/rOpc	[53-57]
Group B OMV + serogroup C CPS	[58]
NspA	[59-64]
N-Propionylated group B CPS	[65]
OMV vaccines made from short-chain lipopolysaccharide mutants of serogroup B	[66]
37-kDa ferric binding protein FbpA	[67]
N-propionyl (NPr) modification of the group B CPS-linked to rPorB	[68]
Group C CPS conjugated to tetanus toxoid	[69]
OMV intranasal vaccine	[70-72]
Rmp, Class 4 OMP	[73]
Anti-idiotype-based peptide mimic vaccine for serogroup C CPS	[74]
Serogroup C CPS and serogroup B OMV conjugate	[75]
LOS-deficient OMV	[76]
Molecular mimetics of CPS epitopes	[77,78]
Reverse vaccinology of vaccine candidates for development of Bexsero 'universal vaccine'	[79-81]
Anti-idiotype based peptide and DNA vaccines which mimic the serogroup C CPS	[82]
GNA33 lipoprotein	[83]
NadA	[84-88]
rPorB	[89]
LOS-tetanus toxoid conjugates	[90]
GNA1870 lipoprotein	[91-94]
Serogroup A CPS conjugates	[95]
Multiepitope DNA vaccine encoding a peptide mimic of serogroup C CPS	[96]
Genome-derived neisserial antigen 2132 (GNA2132)	[97]

Serogroup C peptide mimetics	[98]
Lipoprotein 2086/rLP2086	[99-102]
Monoclonal antibody 9-2-L3,7,9 specific LPS immunotype L3,7,9 and cyclic peptides	[103]
Serogroup C CPS-P64k protein conjugate	[104]
Serogroup A OMV	[105,106]
OpaB and OpaJ	[107]
Outer membrane phospholipase A (OMPLA)	[108]
lpxL mutant OMV	[109]
anti-idiotypic MAb, Naid60, which mimics the serogroup B CPS	[110]
LOS peptide mimetic	[111]
Glycoconjugates of CRM(197) to O-deacylated LPS derived from meningococcal immunotype L3 galE LPS.	[112]
Lactoferrin-binding proteins LbpA and LbpB	[113]
Conjugates of group A and W135 CPS to recombinant *Staphylococcus aureus* enterotoxin C1	[114]
Live *Neisseria lactamica* protects against meningococcal challenge	[115]
OMV over-expressing GNA1870	[116]
Peptide mimics of the group B CPS, including phage displayed	[117-119]
De-O-acetylated (dOA) polysaccharides	[120]
LOS lipid A and serogroup B OMV	[121]
Pool of DNA vaccine candidates (n=20) confer protective immunity against meningococcal infection and induce SBA against serogroup B	[122]
Minor OMPs	[123]
Serogroup W135 polysaccharide-tetanus toxoid conjugate vaccine	[124]
Serogroup B OMV plus TLR adjuvants	[125]
Lipoprotein NMB0928	[126]
Neisseria lactamica OMV	[127]
Factor H-binding protein (fHbp)	[128-139]
OMV with overexpressed factor H-binding protein; and with genetically attenuated endotoxin	[140-142]
De-N-acetylated sialic acid	[143,144]
NMB0088	[145]
Genetically modified serogroup B L3,7 and L2 LOS	[146]
LpxL1 and PagL mutant LOS with OpaJ	[147]
ZnuD	[148,149]
Serogroup B OMV with disabled synX, lpxL1, and lgtA genes	[150]
Serogroup B Ag473	[151]
fHbp and NHBA together	[152]
Opa proteins (n=14)	[153]
rMacrophage Infectivity Potentiator protein	[154,155]
Trivalent native OMV vaccine	[156]
Recombinant PilQ(406-770)	[157]
Adhesin complex protein (ACP)	[158]
Chaperonin60 (Chp60)	[159]
Truncated *Neisseria* hia homologue (NhhA)	[160]
Protein conjugates of carba analogues from serogroup A CPS	[161]
Serogroup X glycoconjugate vaccine	[162]
OMV for serogroup A+W; A+W+X; A+C; X; C	[163-169]
Generalized Modules For Membrane Antigens (GMMA)	[170]
Haemoglobin Receptors, HpuAB and HmbR	[171]

ABC transporter substrate-binding protein, NMB1612	[172]
OMV with constitutive FetA expression	[173,174]
Fully synthetic self-adjuvanting α-2,9-oligosialic acid based conjugate serogroup C vaccine	[175]
OMVs displaying designer glycotopes	[176]
Putative Cell Binding Factor (CBF, NMB0345/NEIS1825)	[177]
DNA vaccine pNMB0315 against serogroup B and rNMB0315	[178,179]
Synthetic oligosaccharide-protein conjugate vaccine for serogroup C	[180]
Stabilized glycomimetic conjugate vaccine for serogroup A	[181]
Adenoviral vectors expressing group B OMPs	[182]
**2. Generation of opsonophagocytic antibodies against meningococci**	
**Antigen**	**References**
Serogroup W-135	[183]
PorB linear epitope	[184]
PorA and PorA peptide	[32,36,38]
Serogroup A, C, Y, or W135 CPS	[185]
Serogroup A OMV	[106]
Serogroup A+W OMV vaccine; AWX-OMV vaccine	[163,165]
**3. Active protection against meningococcal infection**	
**Antigen**	**References**
Group A capsule polysaccharide (CPS) protects mice against homologous challenge	[186]
Mouse bacteraemia protection against five different serotype 2 vaccines.	[187]
OMV containing lipooligosaccharide protects against lethal group B infection and septic shock	[188]
TbpA	[189]
Bordetella pertussis fimbriae- serogroup C conjugate vaccine	[190]
phoP mutant of meningococcus	[191]
Attenuated meningococci YH102 and YH103	[192]
Passive immunization with sera from mice immunized with anti-idiotypic mimics of serogroup B CPS	[193,194]
OMVs and detoxified LOS protect mice against homologous and heterologous serogroup B meningococcal infection and septic shock	[195]
rLP2086 protein immunization reduces colonization in a murine nasal challenge model	[196]
Recombinant penicillin-binding protein 2 (PBP2) immunization conferred protection against meningococcaemia	[197]
Secreted proteins (MSPs) protect against live meningococcal challenge	[198]
Passive protection against meningococcal infection with antibodies to β-(1→6)-linked poly-N-acetyl-d-glucosamine (PNAG)	[199]
**4. Generation of serum bactericidal antibodies against gonococci**	
**Antigen**	**References**
Purified ribosomal fraction of gonococci	[200]
Pathogenic serogroup A strain of *N meningitidis* elicits bactericidal antibodies against gonococci	[201]
mAbs to denatured and native forms of gonococcal outer membrane proteins	[202]
mAbs to pili	[203]
Porin P1/PorB	[204-213]
PorB peptides	[214,215]
OMP III mAbs	[216]

mAb to gonococcal LOS	[217]
Monoclonal anti-idiotope antibody that mimics gonococcal LOS epitope 2C7	[218,219]
2C7 peptide	[220-223]
Recombinant human IgG1 chimeric variant of MAb 2C7 containing an E430G Fc modification (2C7_E430G)	[224]
Recombinant gonococcal transferrin binding proteins (Tbp) A and B conjugated to the cholera toxin B subunit	[225,226]
Opa protein loops	[227]
NspA	[228]
Ghfp, the gonococcal Orthologue of meningococcal fHbp	[229]
Meningococcal truncated MIP	[230]
DNA vaccine using bacterial ghosts	[231]
MtrE and surface-expressed Loop 2	[232]
Gonococcal ACP	[233]
Methionine Sulfoxide Reductase (MsrA/B)	[234]
Meningococcal native OMV vaccine with attenuated endotoxin and overexpressed fHbp	[235]
NGO0416, 0690, 0948, 1043, 1215, 1701	[236]
OMV	[237,238]
NHBA	[239]
Meningococcal 4CMenB vaccine	[240,241]
Phage proteins	[242]
MetQ	[243]
Rmp-deleted mutant strain	[244]
AniA OMP	[245]
**5. Generation of opsonophagocytic antibodies against gonococci**	
**Antigen**	**References**
DNA immunization of mice with a plasmid encoding PorB	[246]
NspA	[228]
PNAG	[199]
PorB	[206,210]
**6. Active protection against gonococcal infection**	**See main text**

Supplementary Table S2. The guinea pig in *Neisseria* research

1. **Vaccine studies**	
**Vaccine tested**	**Reference**
Formalin-fixed gonococcal cells	[247]
Gonococcal outer membrane complex and purified pili	[248]
Meningococcal serotype 2 vaccines	[249]
Gonococcal ribosomal preparations cooper	[250]
Meningococcal serotype 2 OMV, LPS-reduced with group B meningococcal polysaccharide	[251]
Artificial glycoprotein (covalent link of oligosaccharide haptens derived from *Streptococcus pneumoniae* type 6A and *Neisseria meningitidis* group C capsular polysaccharides to non-toxic mutant protein CRM197)	[252]
Epitope C-rich lipopolysaccharide from gonococci	[253]
Meningococcal class I porin	[16]
Recombinant class 3 porin of group B meningococci as carrier protein for a *Haemophilus influenzae* type b (Hib) polyribosylribotol phosphate (PRP) conjugate vaccine	[254]
Meningococcal OMV	[62]
ZnuD-expressing meningococcal OMV	[149]
2. **Infectivity experiments in subcutaneous chambers**	
**Observations with the model**	**Reference**
Infection with gonococcal colony types	[255]
Inability of gonococcal pili to confer protection	[256]
Morphological, biological and antigenic properties of gonococci adapted to growth in subcutaneous chambers	[257]
Gonococcal serotypes and their relationship with immunotypes	[258]
Isolation of gonococcal rough colony type	[259]
Gonococcal strain specificity and immunity to infection	[260]
Comparative physical and immunological aspects of gonococcal infection of the chimpanzee and guinea-pig subcutaneous chamber models	[261]
Phenotypic changes in gonococcal serum resistance	[262]
Strain related infectivity of gonococci and variability of immune resistance in different breeds of guinea pig	[263]
Contribution of antigens contributes to immunogenicity of gonococci	[264]
Environmental factors associated with phenotypically determined resistance of gonococci to normal human serum	[265]
Complex immunogenicity of gonococci	[266]
The role of outer membrane proteins in gonococcal survival	[267]
Characterization of immune response to gonococcal ribosomal preparation	[268]
Effect of anti-pilus antibodies on survival of gonococci	[269]
Chemical and biological characterization of a gonococcal growth inhibitor tested in the model	[270]
Demonstration of capsules on gonococci by electron microscopy of bacteria grown in vivo	[271]
Gonococcal variants selected by growth in vivo or in vitro have antigenically different LPS	[272]
Alterations of the LPS determine gonococcal virulence in the model	[273]
Attenuation and immunogenicity of a gonococcal strain MS11 harbouring a mutation in gene *aroA*	[274]

Supplementary Table S3. Use of the rabbit for testing *Neisseria* vaccine efficacy, through the generation of bactericidal antibodies

1. **Generation of serum bactericidal antibodies against meningococci**	
**Antigen**	**References**
Surface antigens from group A meningococci	[186]
Outer membrane protein or outer membrane protein plus group C polysaccharide	[1]
lipopolysaccharide R-type oligosaccharides conjugated to tetanus toxoid	[275]
H.8 (Lip)	[10]
LOS-derived oligosaccharide-protein conjugates	[42]
PorA synthetic peptides	[34,276]
70 kDa iron-regulated protein (FrpB)	[51]
Transferrin binding protein(s)	[44,46,48,49,189,277]
OMV (PorB response)	[278]
Anti-idiotypic mimics of group B CPS	[193]
Native OMV from lpxL mutant strains	[109]
LPS-based glycoconjugates	[112,279]
Penicillin-binding protein 2 (PBP2) (passive protection)	[197]
Recombinant factor H binding protein (fHbp)	[280-282]
Trivalent native OMV vaccine derived from three serogroup B strains	[283]
Nonavalent PorA native OMV serogroup B vaccine	[30]
Serogroup B PMV with constitutive FetA expression	[174]
OMP-deficient OMVs	[284]
2. **Generation of serum bactericidal antibodies to gonococci**	
**Antigen**	**References**
Protein I (PorB)	[210,285]
Conserved peptide epitope of PorB	[214]
LOS anti-idiotypic antibodies	[217]
Monoclonal anti-idiotope antibody that mimics the LOS epitope 2C7	[218]
2C7 OS epitope mimics - anti-idiotope mAb CA1 and peptide PEP-1	[286]
S. enterica Typhimurium expressing gonococcal filamentous phage Φ6	[287]
Purified filamentous Φfil phages	[288]

Supplementary Table S4. The neonatal (infant) rat model of passive protection used to test the ability of antibodies generated to meningococcal antigens to protect against infection with live meningococci.

**1. Mouse monoclonal antibodies to antigens**	**References**
Class 1 and class 3 OMPs	[289]
Class I (PorA) subtype specific protection	[290]
Rough LOS	[291]
PorB	[292]
Inner-core lipopolysaccharide epitope	[293]
**2. Polyclonal mouse sera to antigen**	**References**
Polyclonal antibodies to serogroup B CPS bind to the brains of infant rats in vitro but not in vivo	[294]
Class 5 proteins	[54]
NsPA	[60]
PorA	[14,16,295]
GNA33	[83]
NadA	[84]
Serogroup B microvesicles	[62]
Lipoprotein GNA1870	[91]
LP2086	[100]
LPS-based glycoconjugates vaccine	[112]
Serogroup B genomic expression libraries	[296]
Single chain variable fragment (scFv), which mimics a protective serogroup B epitope	[194]
Peptide mimics of the group B CPS	[117]
NMB0928	[126]
De-N-acetylated sialic acid	[144]
NMB0088	[145]
NMB0938	[297]
Phage-displayed peptide mimetics of serogroup B CPS	[118]
**3. Rabbit polyclonal sera to antigen**	**References**
LPS in a de-O-acylated J5 lipopolysaccharide (J5 DLPS) as a noncovalent complex with serogroup B OMP protected neutropenic rats against Gram-negative bacteraemia induce by lethal challenge with *P. aeruginosa*	[298]
**4. Human sera to antigen**	**References**
Cuban VA-MENGOCBC vaccine	[299]
Glycoconjugate vaccine for serogroup C	[300]
Serogroup B OMV vaccine	[301]
Inner core LOS epitopes	[302]

Supplementary Table S5. Human-derived cell culture-based models for studying *Neisseria* pathogenesis. Table is adapted from information from the review of Heydarian et al [303].

Species	Site of infection	Model	References
N. gonorrhoeae	Fallopian tube	Long-term ex-vivo Fallopian tube organ culture used to study the effect of gonococcal infection on ciliary activity, bacterial attachment and inflammatory damage. Potentially useful for gonococcal vaccine development.	[304-310]
		Fallopian tube organoids used for long-term in-vitro studies of *C. trachomatis* and could be used to study gonococcal pelvic inflammatory disease.	[311]
	Cornea	Explants of cornea in organ culture used to study gonococcal- epithelial interaction.	[312]
		Cornea organoid from pluripotent stem cells containing three distinct cell types with expression of key epithelial, stromal and endothelial cell markers, could be used to study gonococcal *ophthalmia neonatorum*	[313]
		Monocultures using epithelial cells and/or corneal stromal cells and endothelial cells on Transwell inserts.	[314-317]
		3D cornea model	[318-320]
	Endometrium, Ecto- and endocervix	Endometrium ex-vivo model used to study gonococcal pathogenesis associated with pelvic inflammatory disease.	[321]
		3D endometrial epithelial cell culture model used to study in vivo interactions between *N. gonorrhoeae* and vaginal commensals in a dynamic condition using rotating wall vessel bioreactor technology.	[322]
		Endocervical tissue explanted from patient used to study mechanism of gonococcal invasion through endocervix mucosa.	[323,324]
		Oganoid cultures from ecto- and endocervix promising to use for modelling gonococcal infection.	[325]
		Long-term, hormone-responsive organoid cultures of endometrium.	[326]
		3D polarized, hormone-responsive endometrial tissue models containing stromal cells and organoid-derived epithelial cells on an artificial porous collagen scaffold.	[327]
		Transwell 3D culture model containing polarized endocervical epithelial cells co-cultured with neutrophils useful to study neutrophil influx during gonorrhoea.	[328]
		SIS scaffold-based 3D triple co-culture tissue model included epithelial, fibroblasts, endothelial cells, and neutrophils using perfusion bioreactor system.	[303]
N. meningitidis		3D bronchial epithelial cells and fibroblast cells co-cultured with or without T lymphocytes engineered to study immunophathology of asthma and the bronchial responses against pathogens.	[329,330]

	Upper respiratory tract mucosa	3D bronchial model of fibroblasts suspended in a collagen matrix with differentiated epithelial cells and seeded with eosinophils suitable for studying remodelling events associated with inflammation and asthma.	[331]
		3D bronchial equivalents derived from biopsies of asthmatic and non-asthmatic donors grown on a mesenchymal layer.	[330]
		3D bronchial mucosal model, including a well-differentiated epithelium with functional cilia, mucus secretion, and sub-epithelial fibroblasts	[332]
		3D bronchial mucosal model containing epithelial, fibroblasts and stromal cells that express genes associated with immune responses, apoptosis, mitosis, cell survival, differentiation and cancer.	[333,334]
		3D triple co-culture bronchial mucosal model consists of epithelial cells and fibroblasts and implanted with dendritic cells	[335,336]
		SIS scafold based 3D tissue culture model consisting of the respiratory epithelium, a basement membrane and connective tissue with fibroblasts which is suitable for infection studies with *B. pertussis* and other obligate airway pathogens.	[337]
		Organotypic 3D bronchial and nasal tissue culture models used to study the effect of cigarette smoke on airway biology	[338]
	Blood brain barrier (BBB)	Designed and fabricated of 3D artificial neurovascular model represents 1 millionth the size of adult human brain that allow cell-to-cell communication between endothelial cells, astrocytes, and pericytes and independent perfusion.	[339]
		Construction of 3D artificial BBB on a microfluidic platform included rat derived epithelial cells and neutrophils	[340]
		3D model of human BBB using immortalized brain endothelial cells within a microfluidic chip	[341,342]
		3D model of interconnected neural clusters derived from pluripotent stem cells in a multiscale scaffold consisting of a honeycomb microframe and gelatin nanofibers.	[343]
		3D model of a microfluidic BBB derived from human-induced pluripotent stem cells co-cultured with rat primary astrocytes used for drug permeability screening.	[344]
		3D microvascular model of a microfluidic BBB derived from pluripotent stem cells co-cultured with astrocytes and pericytes that is better phenotypic copy in vivo brain epithelial cells and has the benefit of using human cells that possess BBB barrier properties, and can be used to study barrier destruction, innate immune stimulation, and host-pathogen interaction.	[345-347]
	Meningeal-CSF barrier	Brain endothelial cells derived from induced pluripotent stem cells co-cultured with meningeal cells	[348]
	Vasculature	Ex-vivo model of bacteremia using blood to study passive immunization of antigen-based vaccine candidate.	[349]

		Ex-vivo whole blood model of meningococcal infection to study effect of TLR antagonists	[350]
		Ex-vivo whole blood model of meningococcal infection used to study the effect of anticoagulants on complement activation	[351]
		3D model of perfusable microvascular networks derived from pluripotent stem cells in a microfluidic chip	[352,353]
		3D printed model of artificial vascular networks consists of rigid networks of carbohydrate glass used as a cyto-compatible sacrificial template that could be lined with endothelial cells or other kinds of cells and perfused with blood under high-pressure pulsatile flow.	[354]
		3D model of artificial blood vessels bio-printing using multiple materials, known as bioinks to replicate vasculature, multiple types of cells, and extracellular matrix.	[355]

References

1. Zollinger, W.D.; Mandrell, R.E.; Altieri, P.; Berman, S.; Lowenthal, J.; Artenstein, M.S. Safety and immunogenicity of a Neisseria meningitidis type 2 protein vaccine in animals and humans. *J.Infect.Dis.* **1978**, *137*, 728-739.
2. Jennings, H.J.; Lugowski, C. Immunochemistry of groups A, B and C meningococcal polysaccharide-tetanus toxoid conjugates. *J.Immunol.* **1981**, *127*, 1011-1018.
3. Frasch, C.E.; Peppler, M.S. Protection against group B Neisseria meningitidis disease: preparation of soluble protein and protein-polysaccharide immunogens. *Infect.Immun.* **1982**, *37*, 271-280.
4. Beuvery, E.C.; Miedema, F.; van Delft, R.; Haverkamp, K. Preparation and immunochemical characterisation of meningococcal group C polysaccharide-tetanus toxoid conjugates as a new generation of vaccines. *Infect.Immun.* **1983**, *40*, 39-45.
5. Beuvery, E.C.; Miedema, F.; van Delft, R.W.; Haverkamp, J.; Leussink, A.B.; te Pas, B.J.; Teppema, K.S.; Tiesjema, R.H. Preparation and physicochemical and immunological characterization of polysaccharide-outer membrane protein complexes of Neisseria meningitidis. *Infect Immun* **1983**, *40*, 369-380, doi:10.1128/iai.40.1.369-380.1983.
6. Wang, L.Y.; Frasch, C.E. Development of a Neisseria meningitidis group B serotype 2b protein vaccine and evaluation in a mouse model. *Infect.Immun.* **1984**, *46*, 408-414.
7. Brodeur, B.R.; Larose, Y.; Tsang, P.; Hamel, J.; Ashton, F.; Ryan, A. Protection against infection with neisseria meningitidis group B serotype 2a by passive immunisation with serotype-specific monoclonal antibody. *Infect.Immun.* **1985**, *50*, 510-516.
8. Poolman, J.T.; Beuvery, E.C.; Hopman, C.T.; Witvliet, M.H.; Timmermans, H.A.; Teerlink, T.; Zanen, H.C. Class 1/3 outer membrane protein vaccine against group B, type 15, subtype 16 meningococci. *.Dev.Biol.Stand.* **1986**, *63*, 147-152.
9. Tarkka, E.; Muotiala, A.; Karvonen, M.; Saukkonen-Laitinen, K.; Sarvas, M. Antibody production to a meningococcal outer membrane protein cloned into live *Salmonella typhimurium aroA* vaccine strain. *Microb Pathog* **1989**, *6*, 327-335.
10. Bhattacharjee, A.K.; Moran, E.E.; Zollinger, W.D. Antibodies to meningococcal h.8(lip) antigen fail to show bactericidal activity. *Canadian Journal Of Microbiology* **1990**, *36*, 117-122.
11. Tinsley, C.R.; Virji, M.; Heckels, J.E. Antibodies recognising a variety of different structural motifs on meningococcal Lip antigen fail to demonstrate bactericidal activity. *J.Gen.Microbiol.* **1992**, *138*, 2321-2328.
12. Pettersson, A.; Kuipers, B.; Pelzer, M.; Verhagen, E.; Tiesjema, R.H.; Tommassen, J.; Poolman, J.T. Monoclonal antibodies against the 70 kilodalton iron regulated protein of Neisseria meningitidis are bactericidal and strain specific. *Infect.Immun.* **1990**, *58*, 3036-3041.
13. van der Ley, P.; Poolman, J.T. Construction of a multivalent meningococcal vaccine strain based on the class 1 outer membrane protein. *Infect.Immun.* **1992**, *60*, 3156-3161.
14. Nurminen, M.; Butcher, S.; Idanpaan-Heikkila, I.; Wahlstrom, E.; Muttilainen, S.; Runeberg-Nyman, K.; Sarvas, M.; Makela, P.H. The class 1 outer membrane protein of *Neisseria meningitidis* produced in *Bacillus subtilis* can give rise to protective immunity. *Mol.Microbiol.* **1992**, *6*, 2499-2506.
15. van der Ley, P.; van der Biezen, J.; Hohenstein, P.; Peeters, C.; Poolman, J.T. Use of transformation to construct antigenic hybrids of the class 1 outer membrane protein of Neisseria meningitidis. *Infect.Immun.* **1993**, *61*, 4217-4224.
16. Idanpaan-Heikkila, I.; Muttilainen, S.; Wahlstrom, E.; Saarinen, L.; Leinonen, M.; Sarvas, M.; Makela, P.H. The antibody response to a prototype liposome vaccine containing *Neisseria meningitidis* outer membrane protein P1 produced in *Bacillus subtilis*. *Vaccine* **1995**, *13*, 1501-1508.
17. Idanpaan-Heikkila, I.; Wahlstrom, E.; Muttilainen, S.; Nurminen, M.; Kayhty, H.; Sarvas, M.; Makela, P.H. Immunization with meningococcal class 1 outer membrane protein produced in

Bacillus subtilis and reconstituted in the presence of Zwittergent or Triton X-100. *Vaccine* **1996**, *14*, 886-891.
18. Rouppe van der Voort, E.M.; Kuipers, B.; Brugghe, H.F.; Van Unen, L.M.A.; Timmermans, H.A.M.; Hoogerhout, P.; Poolman, J.T. Epitope specificity of murine and human bactericidal antibodies against PorA P1.7,16 induced with experimental meningococcal group B vaccines. *FEMS Immunol Med Micro* **1997**, *17*, 139-148.
19. Christodoulides, M.; Brooks, J.L.; Rattue, E.; Heckels, J.E. Immunisation with recombinant class 1 outer membrane protein from *Neisseria meningitidis*: influence of liposomes and adjuvants on antibody avidity, recognition of native protein and the induction of a bactericidal immune response against meningococci. *Microbiology* **1998**, *144*, 3027-3037.
20. Peeters, C.C.; Claassen, I.J.; Schuller, M.; Kersten, G.F.; van der Voort, E.M.; Poolman, J.T. Immunogenicity of various presentation forms of PorA outer membrane protein of *Neisseria meningitidis* in mice. *Vaccine* **1999**, *17*, 2702-2712, doi:10.1016/s0264-410x(99)00011-0.
21. Jansen, C.; Kuipers, B.; van der Biezen, J.; de Cock, H.; van der Ley, P.; Tommassen, J. Immunogenicity of in vitro folded outer membrane protein PorA of *Neisseria meningitidis*. *FEMS Immunol Med Micro* **2000**, *27*, 227-233.
22. Niebla, O.; Alvarez, A.; Martin, A.; Rodriguez, A.; Delgado, M.; Falcon, V.; Guillen, G. Immunogenicity of recombinant class 1 protein from *Neisseria meningitidis* refolded into phospholipid vesicles and detergent. *Vaccine* **2001**, *19*, 3568-3574.
23. Arigita, C.; Kersten, G.F.; Hazendonk, T.; Hennink, W.E.; Crommelin, D.J.; Jiskoot, W. Restored functional immunogenicity of purified meningococcal PorA by incorporation into liposomes. *Vaccine* **2003**, *21*, 950-960.
24. Arigita, C.; Bevaart, L.; Everse, L.A.; Koning, G.A.; Hennink, W.E.; Crommelin, D.J.; van de Winkel, J.G.; van Vugt, M.J.; Kersten, G.F.; Jiskoot, W. Liposomal meningococcal B vaccination: role of dendritic cell targeting in the development of a protective immune response. *Infect Immun* **2003**, *71*, 5210-5218.
25. Luijkx, T.A.; van Dijken, H.; Hamstra, H.J.; Kuipers, B.; van der Ley, P.; van Alphen, L.; van den Dobbelsteen, G. Relative immunogenicity of PorA subtypes in a multivalent Neisseria meningitidis vaccine is not dependent on presentation form. *Infection and Immunity* **2003**, *71*, 6367-6371.
26. Humphries, H.E.; Williams, J.N.; Christodoulides, M.; Heckels, J.E. Recombinant meningococcal PorA protein, expressed using a vector system with potential for human vaccination, induces a bactericidal immune response. *Vaccine* **2004**, *22*, 1564-1569, doi:10.1016/j.vaccine.2003.09.042.
27. Humphries, H.; Williams, J.; Blackstone, R.; Jolley, K.A.; Yuen, H.; Christodoulides, M.H., JE. Multivalent liposome-based vaccines containing different serosubtypes of PorA protein induce cross-protective bactericidal immune responses against *Neisseria meningitidis*. *Vaccine* **2006**, *24*, 35-44.
28. Toropainen, M.; Saarinen, L.; Vidarsson, G.; Käyhty, H. Protection by meningococcal outer membrane protein PorA-specific antibodies and a serogroup B capsular polysaccharide-specific antibody in complement-sufficient and C6-deficient infant rats. *Infect Immun* **2006**, *74*, 2803-2808, doi:10.1128/iai.74.5.2803-2808.2006.
29. Pérez, M.E.; Barberá, R.; Domínguez, F.; Otero, O.; Gutiérrez, M.; Falero-Diaz, G.; Sotolongo, F.; Sierra, G. Development and characterization of a murine monoclonal antibody specific for the P1.15 PorA proteins from vaccine strain B:4,7:P1.19,15 of Neisseria meningitidis. *Hybridoma (Larchmt)* **2006**, *25*, 243-247, doi:10.1089/hyb.2006.25.243.
30. Kaaijk, P.; van Straaten, I.; van de Waterbeemd, B.; Boot, E.P.; Levels, L.M.; van Dijken, H.H.; van den Dobbelsteen, G.P. Preclinical safety and immunogenicity evaluation of a nonavalent PorA native outer membrane vesicle vaccine against serogroup B meningococcal disease. *Vaccine* **2013**, *31*, 1065-1071, doi:10.1016/j.vaccine.2012.12.031.

31. Vasquez, A.E.; Manzo, R.A.; Soto, D.A.; Barrientos, M.J.; Maldonado, A.E.; Mosqueira, M.; Avila, A.; Touma, J.; Bruce, E.; Harris, P.R., et al. Oral administration of recombinant Neisseria meningitidis PorA genetically fused to H. pylori HpaA antigen increases antibody levels in mouse serum, suggesting that PorA behaves as a putative adjuvant. *Hum Vaccin Immunother* **2015**, *11*, 776-788, doi:10.1080/21645515.2015.1011011.
32. Afrough, P.; Bouzari, S.; Mousavi, S.F.; Asadi Karam, M.R.; Vaziri, F.; Fateh, A.; Behrouzi, A.; Malekan, M.; Siadat, S.D. Evaluation of immunological responses to recombinant Porin A protein (rPoA) from native strains of Neisseria meningitidis serogroups A and B using OMV as an adjuvant in BALB/c mice. *Microb Pathog* **2017**, *112*, 209-214, doi:10.1016/j.micpath.2017.09.038.
33. Christodoulides, M.; Heckels, J.E. Immunisation with multiple antigen peptides containing B- and T- cell epitopes: production of bactericidal antibodies towards *Neisseria meningitidis*. *Microbiology* **1994**, *140*, 2951-2960.
34. Christodoulides, M.; McGuinness, B.T.; Heckels, J.E. Immunization with synthetic peptides containing epitopes of the class 1 outer-membrane protein of *Neisseria meningitidis*: production of bactericidal antibodies on immunization with a cyclic peptide. *J Gen Microbiol* **1993**, *139*, 1729-1738, doi:10.1099/00221287-139-8-1729.
35. Hoogerhout, P.; Donders, E.M.L.M.; Van den Brink, J.A.M.V.G.; Kuipers, B.; Brugghe, H.F.; Van Unen, L.M.A.; Timmermans, H.A.M.; Ten Hove, G.J.; De Jong, A.P.J.M.; Peeters, C.C.A.M., et al. Conjugates of synthetic cyclic peptides elicit bactericidal antibodies against a conformational epitope on a class 1 outer membrane protein of Neisseria meningitidis. *Infect.Immun.* **1995**, *63*, 3473-3478.
36. Wang, J.; Jarvis, G.A.; Achtman, M.; Rosenqvist, E.; Michaelsen, T.E.; Aase, A.; Griffiss, J.M. Functional activities and immunoglobulin variable regions of human and murine monoclonal antibodies specific for the P1.7 PorA protein loop of Neisseria meningitidis. *Infect Immun* **2000**, *68*, 1871-1878.
37. Menéndez, T.; De Haz, I.; Delgado, M.; Garay, H.; Martín, A.; Vispo, N.S. Immunisation with phage-displayed variable region 2 from meningococcal PorA outer membrane protein induces bactericidal antibodies against Neisseria meningitidis. *Immunol Lett* **2001**, *78*, 143-148, doi:10.1016/s0165-2478(01)00245-0.
38. Michaelsen, T.E.; Kolberg, J.; Aase, A.; Herstad, T.K.; Høiby, E.A. The four mouse IgG isotypes differ extensively in bactericidal and opsonophagocytic activity when reacting with the P1.16 epitope on the outer membrane PorA protein of Neisseria meningitidis. *Scand J Immunol* **2004**, *59*, 34-39, doi:10.1111/j.0300-9475.2004.01362.x.
39. Zhu, D.; Williams, J.N.; Rice, J.; Stevenson, F.K.; Heckels, J.E.; Christodoulides, M. A DNA fusion vaccine induces bactericidal antibodies to a peptide epitope from the PorA porin of *Neisseria meningitidis*. *Infect Immun* **2008**, *76*, 334-338, doi:10.1128/iai.00943-07.
40. Hurpin, C.M.; Carosella, E.D.; Cazenave, P.A. Bactericidal activity of 2 igg2a murine monoclonal-antibodies with distinct fine specificities for group-b neisseria- meningitidis capsular polysaccharide. *Hybridoma* **1992**, *11*, 677-687.
41. Verheul, A.F.M.; Van Gaans, J.A.M.; Wiertz, E.J.H.; Snippe, H.; Verhoef, J.; Poolman, J.T. Meningococcal lipopolysaccharide (LPS)-derived oligosaccharide- protein conjugates evoke outer membrane protein but not LPS specific bactericidal antibodies in mice: influence of adjuvants. *Infect.Immun.* **1993**, *61*, 187-196.
42. Gu, X.X.; Tsai, C.M. Preparation, characterization, and immunogenicity of meningococcal lipooligosaccharide derived oligosaccharide-protein conjugates. *Infect.Immun.* **1993**, *61*, 1873-1880.
43. Danve, B.; Lissolo, L.; Mignon, M.; Dumas, P.; Colombani, S.; Schryvers, A.B.; Quentin-Millet, M.J. Transferrin-binding proteins isolated from Neisseria meningitidis elicit protective and bactericidal antibodies in laboratory animals. *Vaccine* **1993**, *11*, 1214-1220.

44. Lissolo, L.; Maitrewilmotte, G.; Dumas, P.; Mignon, M.; Danve, B.; Quentin-Millet, M.J. Evaluation of transferrin binding protein 2 within the transferrin binding protein complex as a potential antigen for future meningococcal vaccines. *Infection and Immunity* **1995**, *63*, 884-890.
45. Rokbi, B.; Mignon, M.; Maitre-Wilmotte, G.; Lissolo, L.; Danve, B.; Caugant, D.A.; Quentin-Millet, M.J. Evaluation of recombinant transferrin-binding protein B variants from *Neisseria meningitidis* for their ability to induce cross-reactive and bactericidal antibodies against a genetically diverse collection of serogroup B strains. *Infect Immun* **1997**, *65*, 55-63.
46. Ala'Aldeen, D.A.; Borriello, S.P. The meningococcal transferrin-binding proteins 1 and 2 are both surface exposed and generate bactericidal antibodies capable of killing homologous and heterologous strains. *Vaccine* **1996**, *14*, 49-53, doi:10.1016/0264-410x(95)00136-o.
47. Gómez, J.A.; Hernández, E.; Criado, M.T.; Ferreirós, C.M. Effect of adjuvants in the isotypes and bactericidal activity of antibodies against the transferrin-binding proteins of Neisseria meningitidis. *Vaccine* **1998**, *16*, 1633-1639, doi:10.1016/s0264-410x(98)00062-0.
48. Rokbi, B.; Renauld-Mongenie, G.; Mignon, M.; Danve, B.; Poncet, D.; Chabanel, C.; Caugant, D.A.; Quentin-Millet, M.J. Allelic diversity of the two transferrin binding protein B gene isotypes among a collection of Neisseria meningitidis strains representative of serogroup B disease: implication for the composition of a recombinant TbpB-based vaccine. *Infect Immun* **2000**, *68*, 4938-4947, doi:10.1128/iai.68.9.4938-4947.2000.
49. Fegan, J.E.; Calmettes, C.; Islam, E.A.; Ahn, S.K.; Chaudhuri, S.; Yu, R.H.; Gray-Owen, S.D.; Moraes, T.F.; Schryvers, A.B. Utility of Hybrid Transferrin Binding Protein Antigens for Protection Against Pathogenic Neisseria Species. *Front Immunol* **2019**, *10*, 247, doi:10.3389/fimmu.2019.00247.
50. Pettersson, A.; van der Ley, P.; Poolman, J.T.; Tommassen, J. Molecular characterization of the 98-kilodalton iron-regulated outer membrane protein of Neisseria meningitidis. *Infect.Immun.* **1993**, *61*, 4724-4733.
51. Ala'Aldeen, D.A.; Davies, H.A.; Borriello, S.P. Vaccine potential of meningococcal FrpB: studies on surface exposure and functional attributes of common epitopes. *Vaccine* **1994**, *12*, 535-541, doi:10.1016/0264-410x(94)90314-x.
52. Kortekaas, J.; Muller, S.A.; Ringler, P.; Gregorini, M.; Weynants, V.E.; Rutten, L.; Bos, M.P.; Tommassen, J. Immunogenicity and structural characterisation of an in vitro folded meningococcal siderophore receptor (FrpB, FetA). *Microbes Infect* **2006**, *8*, 2145-2153, doi:10.1016/j.micinf.2006.04.011.
53. Jolley, K.; Appleby, L.; Wright, J.C.; Christodoulides, M.; Heckels, J.E. Immunisation with recombinant Opc outer membrane protein from *Neisseria meningitidis*: influence of sequence variation and levels of expression on the bactericidal immune response against meningococci. *Infect Immun* **2001**, *69*, 3809-3916.
54. Sacchi, C.T.; Gorla, M.C.; de Lemos, A.P.; Brandileone, M.C. Considerations on the use of Neisseria meningitidis class 5 proteins as meningococcal BC vaccine components. *Vaccine* **1995**, *13*, 112-118, doi:10.1016/0264-410x(95)80021-5.
55. De Gaspari, E.; Zollinger, W. Expression of class 5 antigens by meningococcal strains obtained from patients in Brazil and evaluation of two new monoclonal antibodies. *Braz J Infect Dis* **2001**, *5*, 143-153, doi:10.1590/s1413-86702001000300007.
56. Carmenate, T.; Mesa, C.; Menéndez, T.; Falcón, V.; Musacchio, A. Recombinant Opc protein from *Neisseria meningitidis* reconstituted into liposomes elicits opsonic antibodies following immunization. *Biotechnol Appl Biochem* **2001**, *34*, 63-69, doi:10.1042/ba20010008.
57. Musacchio, A.; Carmenate, T.; Delgado, M.; Gonzalez, S. Recombinant Opc meningococcal protein, folded in vitro, elicits bactericidal antibodies after immunization. *Vaccine* **1997**, *15*, 751-758.
58. Milagres, L.G.; Cristina, M.; Brandileone, M.C.; Sacchi, C.T.; Vieira, V.S.; Zanella, R.C.; Frasch, C.E. Antibody studies in mice of outer membrane antigens for use in an improved

meningococcal B and C vaccine. *FEMS Immunol Med Microbiol* **1996**, *13*, 9-17, doi:10.1111/j.1574-695X.1996.tb00210.x.
59. Martin, D.; Cadieux, N.; Hamel, J.; Brodeur, B.R. Highly conserved *Neisseria meningitidis* surface protein confers protection against experimental infection. *J Exp Med* **1997**, *185*, 1173-1183.
60. Moe, G.R.; Tan, S.; Granoff, D.M. Differences in surface expression of NspA among Neisseria meningitidis group B strains. *Infect Immun* **1999**, *67*, 5664-5675, doi:10.1128/iai.67.11.5664-5675.1999.
61. Moe, G.R.; Zuno-Mitchell, P.; Lee, S.S.; Lucas, A.H.; Granoff, D.M. Functional activity of anti-Neisserial surface protein A monoclonal antibodies against strains of Neisseria meningitidis serogroup B. *Infect Immun* **2001**, *69*, 3762-3771, doi:10.1128/iai.69.6.3762-3771.2001.
62. Moe, G.R.; Zuno-Mitchell, P.; Hammond, S.N.; Granoff, D.M. Sequential immunization with vesicles prepared from heterologous Neisseria meningitidis strains elicits broadly protective serum antibodies to group B strains. *Infect Immun* **2002**, *70*, 6021-6031, doi:10.1128/iai.70.11.6021-6031.2002.
63. Hou, V.C.; Moe, G.R.; Raad, Z.; Wuorimaa, T.; Granoff, D.M. Conformational epitopes recognized by protective anti-neisserial surface protein A antibodies. *Infect Immun* **2003**, *71*, 6844-6849, doi:10.1128/iai.71.12.6844-6849.2003.
64. Ying, S.; He, J.; Yu, M.; Zhang, Y.; Deng, S.; Zhang, L.; Xie, M.; Hu, S. Recombinant Neisseria surface protein A is a potential vaccine candidate against Neisseria meningitides serogroup B. *Mol Med Rep* **2014**, *10*, 1619-1625, doi:10.3892/mmr.2014.2325.
65. Pon, R.A.; Lussier, M.; Yang, Q.L.; Jennings, H.J. N-Propionylated group B meningococcal polysaccharide mimics a unique bactericidal capsular epitope in group B Neisseria meningitidis. *J Exp Med* **1997**, *185*, 1929-1938, doi:10.1084/jem.185.11.1929.
66. Andersen, S.R.; Bjune, G.; Hoiby, E.A.; Michaelsen, T.E.; Aase, A.; Rye, U.; Jantzen, E. Outer membrane vesicle vaccines made from short-chain lipopolysaccharide mutants of serogroup B Neisseria meningitidis: effect of the carbohydrate chain length on the immune response. *Vaccine* **1997**, *15*, 1225-1234.
67. Gomez, J.A.; Criado, M.T.; Ferreiros, C.M. Bactericidal activity of antibodies elicited against the *Neisseria meningitidis* 37-kDa ferric binding protein (FbpA) with different adjuvants. *FEMS Immunol Med Microbiol* **1998**, *20*, 79-86, doi:S0928-8244(97)00109-0 [pii].
68. Fusco, P.C.; Blake, M.S.; Michon, F. Meningococcal vaccine development: a novel approach. *Expert Opin Investig Drugs* **1998**, *7*, 245-252, doi:10.1517/13543784.7.2.245.
69. Rubinstein, L.J.; García-Ojeda, P.A.; Michon, F.; Jennings, H.J.; Stein, K.E. Murine immune responses to Neisseria meningitidis group C capsular polysaccharide and a thymus-dependent toxoid conjugate vaccine. *Infect Immun* **1998**, *66*, 5450-5456, doi:10.1128/iai.66.11.5450-5456.1998.
70. Saunders, N.B.; Shoemaker, D.R.; Brandt, B.L.; Moran, E.E.; Larsen, T.; Zollinger, W.D. Immunogenicity of intranasally administered meningococcal native outer membrane vesicles in mice. *Infect Immun* **1999**, *67*, 113-119, doi:10.1128/iai.67.1.113-119.1999.
71. Bakke, H.; Lie, K.; Haugen, I.L.; Korsvold, G.E.; Høiby, E.A.; Naess, L.M.; Holst, J.; Aaberge, I.S.; Oftung, F.; Haneberg, B. Meningococcal outer membrane vesicle vaccine given intranasally can induce immunological memory and booster responses without evidence of tolerance. *Infect Immun* **2001**, *69*, 5010-5015, doi:10.1128/iai.69.8.5010-5015.2001.
72. Guthrie, T.; Wong, S.Y.; Liang, B.; Hyland, L.; Hou, S.; Høiby, E.A.; Andersen, S.R. Local and systemic antibody responses in mice immunized intranasally with native and detergent-extracted outer membrane vesicles from Neisseria meningitidis. *Infect Immun* **2004**, *72*, 2528-2537, doi:10.1128/iai.72.5.2528-2537.2004.
73. Rosenqvist, E.; Hoiby, E.A.; Wedege, E.; Bryn, K.; Kolberg, J.; Klem, A.; Ronnild, E.; Bjune, G.; Nokleby, H. Human antibody responses to meningococcal outer membrane antigens after three doses of the Norwegian group B meningococcal vaccine. *Infect Immun* **1995**, *63*, 4642-4652.

74. Hutchins, W.A.; Kieber-Emmons, T.; Carlone, G.M.; Westerink, M.A. Human immune response to a peptide mimic of Neisseria meningitidis serogroup C in hu-PBMC-SCID mice. *Hybridoma* **1999**, *18*, 121-129, doi:10.1089/hyb.1999.18.121.
75. Fukasawa, L.O.; Gorla, M.C.; Schenkman, R.P.; Garcia, L.R.; Carneiro, S.M.; Raw, I.; Tanizaki, M.M. Neisseria meningitidis serogroup C polysaccharide and serogroup B outer membrane vesicle conjugate as a bivalent meningococcus vaccine candidate. *Vaccine* **1999**, *17*, 2951-2958, doi:10.1016/s0264-410x(99)00177-2.
76. Steeghs, L.; Kuipers, B.; Hamstra, H.J.; Kersten, G.; van Alphen, L.; van der Ley, P. Immunogenicity of outer membrane proteins in a lipopolysaccharide-deficient mutant of *Neisseria meningitidis*: Influence of adjuvants on the immune response. *Infection and Immunity* **1999**, *67*, 4988-4993.
77. Moe, G.R.; Tan, S.; Granoff, D.M. Molecular mimetics of polysaccharide epitopes as vaccine candidates for prevention of Neisseria meningitidis serogroup B disease. *FEMS Immunol Med Microbiol* **1999**, *26*, 209-226, doi:10.1111/j.1574-695X.1999.tb01392.x.
78. Grothaus, M.C.; Srivastava, N.; Smithson, S.L.; Kieber-Emmons, T.; Williams, D.B.; Carlone, G.M.; Westerink, M.A. Selection of an immunogenic peptide mimic of the capsular polysaccharide of Neisseria meningitidis serogroup A using a peptide display library. *Vaccine* **2000**, *18*, 1253-1263, doi:10.1016/s0264-410x(99)00390-4.
79. Pizza, M.; Scarlato, V.; Masignani, V.; Giuliani, M.M.; Arico, B.; Comanducci, M.; Jennings, G.T.; Baldi, L.; Bartolini, E.; Capecchi, B., et al. Identification of vaccine candidates against serogroup B meningococcus by whole-genome sequencing. *Science* **2000**, *287*, 1816-1820.
80. Giuliani, M.M.; Adu-Bobie, J.; Comanducci, M.; Arico, B.; Savino, S.; Santini, L.; Brunelli, B.; Bambini, S.; Biolchi, A.; Capecchi, B., et al. A universal vaccine for serogroup B meningococcus. *Proc Natl Acad Sci U S A* **2006**, *103*, 10834-10839, doi:10.1073/pnas.0603940103.
81. Giuliani, M.M.; Biolchi, A.; Serruto, D.; Ferlicca, F.; Vienken, K.; Oster, P.; Rappuoli, R.; Pizza, M.; Donnelly, J. Measuring antigen-specific bactericidal responses to a multicomponent vaccine against serogroup B meningococcus. *Vaccine* **2010**, *28*, 5023-5030, doi:10.1016/j.vaccine.2010.05.014.
82. Westerink, M.A.; Smithson, S.L.; Hutchins, W.A.; Widera, G. Development and characterization of anti-idiotype based peptide and DNA vaccines which mimic the capsular polysaccharide of *Neisseria meningitidis* serogroup C. *International reviews of immunology* **2001**, *20*, 251-261.
83. Granoff, D.M.; Moe, G.R.; Giuliani, M.M.; Adu-Bobie, J.; Santini, L.; Brunelli, B.; Piccinetti, F.; Zuno-Mitchell, P.; Lee, S.S.; Neri, P., et al. A novel mimetic antigen eliciting protective antibody to Neisseria meningitidis. *J Immunol* **2001**, *167*, 6487-6496, doi:10.4049/jimmunol.167.11.6487.
84. Comanducci, M.; Bambini, S.; Brunelli, B.; Adu-Bobie, J.; Arico, B.; Capecchi, B.; Giuliani, M.M.; Masignani, V.; Santini, L.; Savino, S., et al. NadA, a novel vaccine candidate of *Neisseria meningitidis*. *J Exp Med* **2002**, *195*, 1445-1454.
85. Bowe, F.; Lavelle, E.C.; McNeela, E.A.; Hale, C.; Clare, S.; Arico, B.; Giuliani, M.M.; Rae, A.; Huett, A.; Rappuoli, R., et al. Mucosal vaccination against serogroup B meningococci: induction of bactericidal antibodies and cellular immunity following intranasal immunization with NadA of Neisseria meningitidis and mutants of Escherichia coli heat-labile enterotoxin. *Infect Immun* **2004**, *72*, 4052-4060, doi:10.1128/iai.72.7.4052-4060.2004.
86. Comanducci, M.; Bambini, S.; Caugant, D.A.; Mora, M.; Brunelli, B.; Capecchi, B.; Ciucchi, L.; Rappuoli, R.; Pizza, M. NadA diversity and carriage in Neisseria meningitidis. *Infect Immun* **2004**, *72*, 4217-4223, doi:10.1128/iai.72.7.4217-4223.2004.
87. Ciabattini, A.; Giomarelli, B.; Parigi, R.; Chiavolini, D.; Pettini, E.; Arico, B.; Giuliani, M.M.; Santini, L.; Medaglini, D.; Pozzi, G. Intranasal immunization of mice with recombinant *Streptococcus gordonii* expressing NadA of *Neisseria meningitidis* induces systemic bactericidal antibodies and local IgA. *Vaccine* **2008**, *26*, 4244-4250, doi:10.1016/j.vaccine.2008.05.049.

88. Bertoldi, I.; Faleri, A.; Galli, B.; Lo Surdo, P.; Liguori, A.; Norais, N.; Santini, L.; Masignani, V.; Pizza, M.; Giuliani, M.M. Exploiting chimeric human antibodies to characterize a protective epitope of Neisseria adhesin A, one of the Bexsero vaccine components. *FASEB journal : official publication of the Federation of American Societies for Experimental Biology* **2016**, *30*, 93-101, doi:10.1096/fj.15-273813.
89. Wright, J.C.; Williams, J.N.; Christodoulides, M.; Heckels, J.E. Immunisation with recombinant PorB outer membrane protein induces a bactericidal immune response against *Neisseria meningitidis*. *Infect Immun* **2002**, *70*, 4028-4034.
90. Mieszala, M.; Kogan, G.; Jennings, H.J. Conjugation of meningococcal lipooligosaccharides through their lipid A terminus conserves their inner epitopes and results in conjugate vaccines having improved immunological properties. *Carbohydr Res* **2003**, *338*, 167-175.
91. Masignani, V.; Comanducci, M.; Giuliani, M.M.; Bambini, S.; Adu-Bobie, J.; Arico, B.; Brunelli, B.; Pieri, A.; Santini, L.; Savino, S., et al. Vaccination against *Neisseria meningitidis* using three variants of the lipoprotein GNA1870. *J Exp.Med.* **2003**, *197*, 789-799.
92. Welsch, J.A.; Rossi, R.; Comanducci, M.; Granoff, D.M. Protective activity of monoclonal antibodies to genome-derived neisserial antigen 1870, a Neisseria meningitidis candidate vaccine. *J Immunol* **2004**, *172*, 5606-5615, doi:10.4049/jimmunol.172.9.5606.
93. Giuliani, M.M.; Santini, L.; Brunelli, B.; Biolchi, A.; Aricò, B.; Di Marcello, F.; Cartocci, E.; Comanducci, M.; Masignani, V.; Lozzi, L., et al. The region comprising amino acids 100 to 255 of Neisseria meningitidis lipoprotein GNA 1870 elicits bactericidal antibodies. *Infect Immun* **2005**, *73*, 1151-1160, doi:10.1128/iai.73.2.1151-1160.2005.
94. Hou, V.C.; Koeberling, O.; Welsch, J.A.; Granoff, D.M. Protective antibody responses elicited by a meningococcal outer membrane vesicle vaccine with overexpressed genome-derived neisserial antigen 1870. *J Infect Dis* **2005**, *192*, 580-590, doi:JID34306 [pii];10.1086/432102 [doi].
95. Jin, Z.; Chu, C.; Robbins, J.B.; Schneerson, R. Preparation and characterization of group A meningococcal capsular polysaccharide conjugates and evaluation of their immunogenicity in mice. *Infect Immun* **2003**, *71*, 5115-5120, doi:10.1128/iai.71.9.5115-5120.2003.
96. Prinz, D.M.; Smithson, S.L.; Kieber-Emmons, T.; Westerink, M.A. Induction of a protective capsular polysaccharide antibody response to a multiepitope DNA vaccine encoding a peptide mimic of meningococcal serogroup C capsular polysaccharide. *Immunology* **2003**, *110*, 242-249.
97. Welsch, J.A.; Moe, G.R.; Rossi, R.; Adu-Bobie, J.; Rappuoli, R.; Granoff, D.M. Antibody to genome-derived neisserial antigen 2132, a *Neisseria meningitidis* candidate vaccine, confers protection against bacteremia in the absence of complement-mediated bactericidal activity. *J Infect Dis* **2003**, *188*, 1730-1740, doi:10.1086/379375.
98. Prinz, D.M.; Smithson, S.L.; Westerink, M.A. Two different methods result in the selection of peptides that induce a protective antibody response to Neisseria meningitidis serogroup C. *J Immunol Methods* **2004**, *285*, 1-14, doi:10.1016/j.jim.2003.08.005.
99. Fletcher, L.D.; Bernfield, L.; Barniak, V.; Farley, J.E.; Howell, A.; Knauf, M.; Ooi, P.; Smith, R.P.; Weise, P.; Wetherell, M., et al. Vaccine potential of the *Neisseria meningitidis* 2086 lipoprotein. *Infect Immun* **2004**, *72*, 2088-2100.
100. Pillai, S.; Howell, A.; Alexander, K.; Bentley, B.E.; Jiang, H.Q.; Ambrose, K.; Zhu, D.; Zlotnick, G. Outer membrane protein (OMP) based vaccine for Neisseria meningitidis serogroup B. *Vaccine* **2005**, *23*, 2206-2209, doi:10.1016/j.vaccine.2005.01.056.
101. Zhu, D.; Barniak, V.; Zhang, Y.; Green, B.; Zlotnick, G. Intranasal immunization of mice with recombinant lipidated P2086 protein reduces nasal colonization of group B Neisseria meningitidis. *Vaccine* **2006**, *24*, 5420-5425, doi:10.1016/j.vaccine.2006.03.051.
102. Mascioni, A.; Bentley, B.E.; Camarda, R.; Dilts, D.A.; Fink, P.; Gusarova, V.; Hoiseth, S.K.; Jacob, J.; Lin, S.L.; Malakian, K., et al. Structural Basis for the Immunogenic Properties of the Meningococcal Vaccine Candidate LP2086. *J Biol Chem* **2009**, *284*, 8738-8746, doi:10.1074/jbc.M808831200.

103. Lauvrak, V.; Berntzen, G.; Heggelund, U.; Herstad, T.K.; Sandin, R.H.; Dalseg, R.; Rosenqvist, E.; Sandlie, I.; Michaelsen, T.E. Selection and characterization of cyclic peptides that bind to a monoclonal antibody against meningococcal L3,7,9 lipopolysaccharides. *Scand J Immunol* **2004**, *59*, 373-384, doi:10.1111/j.1365-3083.2004.01400.x.
104. Carmenate, T.; Canaán, L.; Alvarez, A.; Delgado, M.; González, S.; Menéndez, T.; Rodés, L.; Guillén, G. Effect of conjugation methodology on the immunogenicity and protective efficacy of meningococcal group C polysaccharide-P64k protein conjugates. *FEMS Immunol Med Microbiol* **2004**, *40*, 193-199, doi:10.1016/s0928-8244(03)00346-8.
105. Norheim, G.; Arne Høiby, E.; Caugant, D.A.; Namork, E.; Tangen, T.; Fritzsønn, E.; Rosenqvist, E. Immunogenicity and bactericidal activity in mice of an outer membrane protein vesicle vaccine against Neisseria meningitidis serogroup A disease. *Vaccine* **2004**, *22*, 2171-2180, doi:10.1016/j.vaccine.2003.11.047.
106. Norheim, G.; Aase, A.; Caugant, D.A.; Høiby, E.A.; Fritzsønn, E.; Tangen, T.; Kristiansen, P.; Heggelund, U.; Rosenqvist, E. Development and characterisation of outer membrane vesicle vaccines against serogroup A Neisseria meningitidis. *Vaccine* **2005**, *23*, 3762-3774, doi:10.1016/j.vaccine.2005.02.021.
107. de Jonge, M.I.; Hamstra, H.J.; Jiskoot, W.; Roholl, P.; Williams, N.A.; Dankert, J.; van Alphen, L.; van der Ley, P. Intranasal immunisation of mice with liposomes containing recombinant meningococcal OpaB and OpaJ proteins. *Vaccine* **2004**, *22*, 4021-4028, doi:10.1016/j.vaccine.2004.03.047.
108. Bos, M.P.; Tefsen, B.; Voet, P.; Weynants, V.; van Putten, J.P.; Tommassen, J. Function of neisserial outer membrane phospholipase A in autolysis and assessment of its vaccine potential. *Infect Immun* **2005**, *73*, 2222-2231, doi:10.1128/iai.73.4.2222-2231.2005.
109. Fisseha, M.; Chen, P.; Brandt, B.; Kijek, T.; Moran, E.; Zollinger, W. Characterization of native outer membrane vesicles from lpxL mutant strains of Neisseria meningitidis for use in parenteral vaccination. *Infect Immun* **2005**, *73*, 4070-4080, doi:10.1128/iai.73.7.4070-4080.2005.
110. Park, I.H.; Youn, J.H.; Choi, I.H.; Nahm, M.H.; Kim, S.J.; Shin, J.S. Anti-idiotypic antibody as a potential candidate vaccine for Neisseria meningitidis serogroup B. *Infect Immun* **2005**, *73*, 6399-6406, doi:10.1128/iai.73.10.6399-6406.2005.
111. Tiwana, H.; Clow, K.J.; Hall, C.; Feavers, I.M.; Charalambous, B.M. The immunogenicity of a conformationally restricted peptide mimetic of meningococcal lipooligosaccharide. *Scand J Immunol* **2005**, *62*, 385-392, doi:10.1111/j.1365-3083.2005.01671.x.
112. Cox, A.D.; Zou, W.; Gidney, M.A.; Lacelle, S.; Plested, J.S.; Makepeace, K.; Wright, J.C.; Coull, P.A.; Moxon, E.R.; Richards, J.C. Candidacy of LPS-based glycoconjugates to prevent invasive meningococcal disease: developmental chemistry and investigation of immunological responses following immunization of mice and rabbits. *Vaccine* **2005**, *23*, 5045-5054, doi:10.1016/j.vaccine.2005.06.011.
113. Pettersson, A.; Kortekaas, J.; Weynants, V.E.; Voet, P.; Poolman, J.T.; Bos, M.P.; Tommassen, J. Vaccine potential of the *Neisseria meningitidis* lactoferrin-binding proteins LbpA and LbpB. *Vaccine* **2006**, *24*, 3545-3557, doi:10.1016/j.vaccine.2006.02.003.
114. Jin, Z.; Bohach, G.A.; Shiloach, J.; Norris, S.E.; Freedberg, D.I.; Deobald, C.; Coxon, B.; Robbins, J.B.; Schneerson, R. Conjugates of group A and W135 capsular polysaccharides of neisseria meningitidis bound to recombinant Staphylococcus aureus enterotoxin C1: preparation, physicochemical characterization, and immunological properties in mice. *Infect Immun* **2005**, *73*, 7887-7893, doi:10.1128/iai.73.12.7887-7893.2005.
115. Li, Y.; Zhang, Q.; Winterbotham, M.; Mowe, E.; Gorringe, A.; Tang, C.M. Immunization with live *Neisseria lactamica* protects mice against meningococcal challenge and can elicit serum bactericidal antibodies. *Infect Immun* **2006**, *74*, 6348-6355, doi:10.1128/iai.01062-06.
116. Koeberling, O.; Welsch, J.A.; Granoff, D.M. Improved immunogenicity of a H44/76 group B outer membrane vesicle vaccine with over-expressed genome-derived Neisserial antigen 1870. *Vaccine* **2007**, *25*, 1912-1920, doi:10.1016/j.vaccine.2006.03.092.

117. Lo Passo, C.; Romeo, A.; Pernice, I.; Donato, P.; Midiri, A.; Mancuso, G.; Arigo, M.; Biondo, C.; Galbo, R.; Papasergi, S., et al. Peptide mimics of the group B meningococcal capsule induce bactericidal and protective antibodies after immunization. *J Immunol* **2007**, *178*, 4417-4423.
118. Menéndez, T.; Santiago-Vispo, N.F.; Cruz-Leal, Y.; Coizeau, E.; Garay, H.; Reyes, O.; Batista, Y.; Cobas, K.; Carmenate, T.; Chinea, G., et al. Identification and characterization of phage-displayed peptide mimetics of Neisseria meningitidis serogroup B capsular polysaccharide. *Int J Med Microbiol* **2011**, *301*, 16-25, doi:10.1016/j.ijmm.2010.04.020.
119. Garay, H.; Menendez, T.; Cruz-Leal, Y.; Coizeau, E.; Noda, J.; Morera, V.; Guillen, G.; Albericio, F.; Reyes, O. Study of various presentation forms for a peptide mimetic of *Neisseria meningitidis* serogroup B capsular polysaccharide. *Bioconjug Chem* **2011**, *22*, 33-41, doi:10.1021/bc100299g.
120. Fusco, P.C.; Farley, E.K.; Huang, C.H.; Moore, S.; Michon, F. Protective meningococcal capsular polysaccharide epitopes and the role of O acetylation. *Clin Vaccine Immunol* **2007**, *14*, 577-584, doi:10.1128/cvi.00009-07.
121. Zughaier, S.; Steeghs, L.; van der Ley, P.; Stephens, D.S. TLR4-dependent adjuvant activity of Neisseria meningitidis lipid A. *Vaccine* **2007**, *25*, 4401-4409, doi:10.1016/j.vaccine.2007.03.029.
122. Yero, D.; Pajon, R.; Perez, Y.; Farinas, M.; Cobas, K.; Diaz, D.; Solis, R.L.; Acosta, A.; Brookes, C.; Taylor, S., et al. Identification by genomic immunization of a pool of DNA vaccine candidates that confer protective immunity in mice against *Neisseria meningitidis* serogroup B. *Vaccine* **2007**, *25*, 5175-5188, doi:10.1016/j.vaccine.2007.04.084.
123. Weynants, V.E.; Feron, C.M.; Goraj, K.K.; Bos, M.P.; Denoel, P.A.; Verlant, V.G.; Tommassen, J.; Peak, I.R.; Judd, R.C.; Jennings, M.P., et al. Additive and synergistic bactericidal activity of antibodies directed against minor outer membrane proteins of *Neisseria meningitidis*. *Infect Immun* **2007**, *75*, 5434-5442, doi:10.1128/iai.00411-07.
124. Gudlavalleti, S.K.; Lee, C.H.; Norris, S.E.; Paul-Satyaseela, M.; Vann, W.F.; Frasch, C.E. Comparison of Neisseria meningitidis serogroup W135 polysaccharide-tetanus toxoid conjugate vaccines made by periodate activation of O-acetylated, non-O-acetylated and chemically de-O-acetylated polysaccharide. *Vaccine* **2007**, *25*, 7972-7980, doi:10.1016/j.vaccine.2007.06.018.
125. Fransen, F.; Boog, C.J.; van Putten, J.P.; van der Ley, P. Agonists of Toll-like receptors 3, 4, 7, and 9 are candidates for use as adjuvants in an outer membrane vaccine against Neisseria meningitidis serogroup B. *Infect Immun* **2007**, *75*, 5939-5946, doi:10.1128/iai.00846-07.
126. Delgado, M.; Yero, D.; Niebla, O.; Gonzalez, S.; Climent, Y.; Perez, Y.; Cobas, K.; Caballero, E.; Garcia, D.; Pajon, R. Lipoprotein NMB0928 from *Neisseria meningitidis* serogroup B as a novel vaccine candidate. *Vaccine* **2007**, *25*, 8420-8431, doi:10.1016/j.vaccine.2007.09.053.
127. Finney, M.; Vaughan, T.; Taylor, S.; Hudson, M.J.; Pratt, C.; Wheeler, J.X.; Vipond, C.; Feavers, I.; Jones, C.; Findlow, J., et al. Characterization of the key antigenic components and pre-clinical immune responses to a meningococcal disease vaccine based on Neisseria lactamica outer membrane vesicles. *Hum Vaccin* **2008**, *4*, 23-30, doi:10.4161/hv.4.1.4806.
128. Beernink, P.T.; Granoff, D.M. Bactericidal antibody responses induced by meningococcal recombinant chimeric factor H-binding protein vaccines. *Infect Immun* **2008**, *76*, 2568-2575, doi:10.1128/iai.00033-08.
129. Beernink, P.T.; Welsch, J.A.; Bar-Lev, M.; Koeberling, O.; Comanducci, M.; Granoff, D.M. Fine antigenic specificity and cooperative bactericidal activity of monoclonal antibodies directed at the meningococcal vaccine candidate factor h-binding protein. *Infect Immun* **2008**, *76*, 4232-4240, doi:10.1128/iai.00367-08.
130. Brunelli, B.; Del Tordello, E.; Palumbo, E.; Biolchi, A.; Bambini, S.; Comanducci, M.; Muzzi, A.; Pizza, M.; Rappuoli, R.; Donnelly, J.J., et al. Influence of sequence variability on bactericidal activity sera induced by Factor H binding protein variant 1.1. *Vaccine* **2011**, *29*, 1072-1081, doi:10.1016/j.vaccine.2010.11.064.
131. Seib, K.L.; Brunelli, B.; Brogioni, B.; Palumbo, E.; Bambini, S.; Muzzi, A.; DiMarcello, F.; Marchi, S.; van der Ende, A.; Aricó, B., et al. Characterization of diverse subvariants of the

meningococcal factor H (fH) binding protein for their ability to bind fH, to mediate serum resistance, and to induce bactericidal antibodies. *Infect Immun* **2011**, *79*, 970-981, doi:10.1128/iai.00891-10.
132. Beernink, P.T.; Shaughnessy, J.; Braga, E.M.; Liu, Q.; Rice, P.A.; Ram, S.; Granoff, D.M. A meningococcal factor H binding protein mutant that eliminates factor H binding enhances protective antibody responses to vaccination. *J Immunol* **2011**, *186*, 3606-3614, doi:10.4049/jimmunol.1003470.
133. Koeberling, O.; Delany, I.; Granoff, D.M. A critical threshold of meningococcal factor H binding protein expression is required for increased breadth of protective antibodies elicited by native outer membrane vesicle vaccines. *Clin Vaccine Immunol* **2011**, *18*, 736-742, doi:10.1128/cvi.00542-10.
134. Giuntini, S.; Reason, D.C.; Granoff, D.M. Complement-mediated bactericidal activity of anti-factor H binding protein monoclonal antibodies against the meningococcus relies upon blocking factor H binding. *Infect Immun* **2011**, *79*, 3751-3759, doi:10.1128/iai.05182-11.
135. Giuntini, S.; Reason, D.C.; Granoff, D.M. Combined roles of human IgG subclass, alternative complement pathway activation, and epitope density in the bactericidal activity of antibodies to meningococcal factor h binding protein. *Infect Immun* **2012**, *80*, 187-194, doi:10.1128/iai.05956-11.
136. Beernink, P.T.; Shaughnessy, J.; Pajon, R.; Braga, E.M.; Ram, S.; Granoff, D.M. The effect of human factor H on immunogenicity of meningococcal native outer membrane vesicle vaccines with over-expressed factor H binding protein. *PLoS Pathog* **2012**, *8*, e1002688, doi:10.1371/journal.ppat.1002688.
137. Rossi, R.; Granoff, D.M.; Beernink, P.T. Meningococcal factor H-binding protein vaccines with decreased binding to human complement factor H have enhanced immunogenicity in human factor H transgenic mice. *Vaccine* **2013**, *31*, 5451-5457, doi:10.1016/j.vaccine.2013.08.099.
138. van der Veen, S.; Johnson, S.; Jongerius, I.; Malik, T.; Genovese, A.; Santini, L.; Staunton, D.; Ufret-Vincenty, R.L.; Pickering, M.C.; Lea, S.M., et al. Nonfunctional variant 3 factor H binding proteins as meningococcal vaccine candidates. *Infect Immun* **2014**, *82*, 1157-1163, doi:10.1128/iai.01183-13.
139. Price, G.A.; Bash, M.C. Development of an FHbp-CTB holotoxin-like chimera and the elicitation of bactericidal antibodies against serogroup B Neisseria meningitidis. *Vaccine* **2018**, *36*, 644-652, doi:10.1016/j.vaccine.2017.12.045.
140. Koeberling, O.; Seubert, A.; Granoff, D.M. Bactericidal antibody responses elicited by a meningococcal outer membrane vesicle vaccine with overexpressed factor H-binding protein and genetically attenuated endotoxin. *J Infect Dis* **2008**, *198*, 262-270, doi:10.1086/589308.
141. Koeberling, O.; Giuntini, S.; Seubert, A.; Granoff, D.M. Meningococcal outer membrane vesicle vaccines derived from mutant strains engineered to express factor H binding proteins from antigenic variant groups 1 and 2. *Clin Vaccine Immunol* **2009**, *16*, 156-162, doi:10.1128/cvi.00403-08.
142. Pajon, R.; Lujan, E.; Granoff, D.M. A meningococcal NOMV-FHbp vaccine for Africa elicits broader serum bactericidal antibody responses against serogroup B and non-B strains than a licensed serogroup B vaccine. *Vaccine* **2016**, *34*, 643-649, doi:10.1016/j.vaccine.2015.12.034.
143. Moe, G.R.; Bhandari, T.S.; Flitter, B.A. Vaccines containing de-N-acetyl sialic acid elicit antibodies protective against *Neisseria meningitidis* groups B and C. *J Immunol* **2009**, *182*, 6610-6617, doi:10.4049/jimmunol.0803677.
144. Moe, G.R.; Flitter, B.A.; Ing, J.Y.; Bhandari, T.S.; Kaur, H. De-N-acetylated sialic acid is immunogenic and elicits antibodies that are protective against Neisseria meningitidis. *Vaccimonitor* **2009**, *18*, 61-65.
145. Sardinas, G.; Yero, D.; Climent, Y.; Caballero, E.; Cobas, K.; Niebla, O. *Neisseria meningitidis* antigen NMB0088: sequence variability, protein topology and vaccine potential. *J Med Microbiol* **2009**, *58*, 196-208, doi:10.1099/jmm.0.004820-0.

146. Weynants, V.; Denoel, P.; Devos, N.; Janssens, D.; Feron, C.; Goraj, K.; Momin, P.; Monnom, D.; Tans, C.; Vandercammen, A., et al. Genetically modified L3,7 and L2 lipooligosaccharides from *Neisseria meningitidis* serogroup B confer a broad cross-bactericidal response. *Infect Immun* **2009**, *77*, 2084-2093, doi:10.1128/iai.01108-08.
147. Arenas, J.; van Dijken, H.; Kuipers, B.; Hamstra, H.J.; Tommassen, J.; van der Ley, P. Coincorporation of LpxL1 and PagL mutant lipopolysaccharides into liposomes with *Neisseria meningitidis* opacity protein: influence on endotoxic and adjuvant activity. *Clin Vaccine Immunol* **2010**, *17*, 487-495, doi:10.1128/cvi.00423-09.
148. Stork, M.; Bos, M.P.; Jongerius, I.; de Kok, N.; Schilders, I.; Weynants, V.E.; Poolman, J.T.; Tommassen, J. An outer membrane receptor of *Neisseria meningitidis* involved in zinc acquisition with vaccine potential. *PLoS Pathog* **2010**, *6*, e1000969, doi:10.1371/journal.ppat.1000969.
149. Hubert, K.; Devos, N.; Mordhorst, I.; Tans, C.; Baudoux, G.; Feron, C.; Goraj, K.; Tommassen, J.; Vogel, U.; Poolman, J.T., et al. ZnuD, a potential candidate for a simple and universal Neisseria meningitidis vaccine. *Infect Immun* **2013**, *81*, 1915-1927, doi:10.1128/iai.01312-12.
150. Zollinger, W.D.; Donets, M.A.; Schmiel, D.H.; Pinto, V.B.; Labrie, J.E., 3rd; Moran, E.E.; Brandt, B.L.; Ionin, B.; Marques, R.; Wu, M., et al. Design and evaluation in mice of a broadly protective meningococcal group B native outer membrane vesicle vaccine. *Vaccine* **2010**, *28*, 5057-5067, doi:10.1016/j.vaccine.2010.05.006.
151. Sung, J.W.; Hsieh, S.Y.; Lin, C.L.; Leng, C.H.; Liu, S.J.; Chou, A.H.; Lai, L.W.; Lin, L.H.; Kwok, Y.; Yang, C.Y., et al. Biochemical characterizations of *Escherichia coli*-expressed protective antigen Ag473 of *Neisseria meningitides* group B. *Vaccine* **2010**, *28*, 8175-8182, doi:10.1016/j.vaccine.2010.09.091.
152. Vu, D.M.; Wong, T.T.; Granoff, D.M. Cooperative serum bactericidal activity between human antibodies to meningococcal factor H binding protein and neisserial heparin binding antigen. *Vaccine* **2011**, *29*, 1968-1973, doi:10.1016/j.vaccine.2010.12.075.
153. Callaghan, M.J.; Lewis, S.; Sadarangani, M.; Bailey, S.E.; Chan, H.; Ferguson, D.J.; Derrick, J.P.; Feavers, I.; Maiden, M.C.; Pollard, A.J. Potential of recombinant opa proteins as vaccine candidates against hyperinvasive meningococci. *Infect Immun* **2011**, *79*, 2810-2818, doi:10.1128/iai.01338-10.
154. Bielecka, M.K.; Devos, N.; Gilbert, M.; Hung, M.C.; Weynants, V.; Heckels, J.E.; Christodoulides, M. Recombinant protein truncation strategy for inducing bactericidal antibodies to the macrophage infectivity potentiator protein of *Neisseria meningitidis* and circumventing potential cross-reactivity with human FK506-binding proteins. *Infect Immun* **2015**, *83*, 730-742, doi:10.1128/iai.01815-14.
155. Hung, M.C.; Salim, O.; Williams, J.N.; Heckels, J.E.; Christodoulides, M. The *Neisseria meningitidis* macrophage infectivity potentiator protein induces cross-strain serum bactericidal activity and is a potential serogroup B vaccine candidate. *Infect Immun* **2011**, *79*, 3784-3791, doi:10.1128/iai.05019-11.
156. Pinto, V.B.; Moran, E.E.; Cruz, F.; Wang, X.M.; Fridman, A.; Zollinger, W.D.; Przysiecki, C.T.; Burden, R. An experimental outer membrane vesicle vaccine from N. meningitidis serogroup B strains that induces serum bactericidal activity to multiple serogroups. *Vaccine* **2011**, *29*, 7752-7758, doi:10.1016/j.vaccine.2011.07.124.
157. Haghi, F.; Peerayeh, S.N.; Siadat, S.D.; Zeighami, H. Recombinant outer membrane secretin PilQ(406-770) as a vaccine candidate for serogroup B *Neisseria meningitidis*. *Vaccine* **2012**, *30*, 1710-1714, doi:10.1016/j.vaccine.2011.12.076.
158. Hung, M.C.; Heckels, J.E.; Christodoulides, M. The adhesin complex protein (ACP) of *Neisseria meningitidis* is a new adhesin with vaccine potential. *mBio* **2013**, *4(2)*, pii: e00041-00013. doi: 00010.01128/mBio.00041-00013., doi:10.1128/mBio.00041-13.
159. Phillips, R.; Williams, J.N.; Tan, W.M.; Bielecka, M.K.; Thompson, H.; Hung, M.C.; Heckels, J.E.; Christodoulides, M. Immunization with recombinant Chaperonin60 (Chp60) outer membrane

protein induces a bactericidal antibody response against *Neisseria meningitidis*. *Vaccine* **2013**, *31*, 2584-2590, doi:S0264-410X(13)00368-X [pii];10.1016/j.vaccine.2013.03.033 [doi].
160. Peak, I.R.; Srikhanta, Y.N.; Weynants, V.E.; Feron, C.; Poolman, J.T.; Jennings, M.P. Evaluation of truncated NhhA protein as a candidate meningococcal vaccine antigen. *PLoS One* **2013**, *8*, e72003, doi:10.1371/journal.pone.0072003.
161. Gao, Q.; Tontini, M.; Brogioni, G.; Nilo, A.; Filippini, S.; Harfouche, C.; Polito, L.; Romano, M.R.; Costantino, P.; Berti, F., et al. Immunoactivity of protein conjugates of carba analogues from Neisseria meningitidis a capsular polysaccharide. *ACS Chem Biol* **2013**, *8*, 2561-2567, doi:10.1021/cb400463u.
162. Micoli, F.; Romano, M.R.; Tontini, M.; Cappelletti, E.; Gavini, M.; Proietti, D.; Rondini, S.; Swennen, E.; Santini, L.; Filippini, S., et al. Development of a glycoconjugate vaccine to prevent meningitis in Africa caused by meningococcal serogroup X. *Proc Natl Acad Sci U S A* **2013**, *110*, 19077-19082, doi:10.1073/pnas.1314476110.
163. Tunheim, G.; Arnemo, M.; Næss, L.M.; Fjeldheim Å, K.; Nome, L.; Bolstad, K.; Aase, A.; Mandiarote, A.; González, H.; González, D., et al. Preclinical immunogenicity and functional activity studies of an A+W meningococcal outer membrane vesicle (OMV) vaccine and comparisons with existing meningococcal conjugate- and polysaccharide vaccines. *Vaccine* **2013**, *31*, 6097-6106, doi:10.1016/j.vaccine.2013.09.044.
164. Romeu, B.; Lastre, M.; García, L.; Cedré, B.; Mandariote, A.; Fariñas, M.; Oliva, R.; Rosenqvist, E.; Pérez, O. Combined meningococcal serogroup A and W135 outer-membrane vesicles activate cell-mediated immunity and long-term memory responses against non-covalent capsular polysaccharide A. *Immunol Res* **2014**, *58*, 75-85, doi:10.1007/s12026-013-8427-6.
165. Tunheim, G.; Naess, L.M.; Acevedo, R.; Fjeldheim Å, K.; Bolstad, K.; García, L.; Cardoso, D.; Aase, A.; Zayas, C.; González, H., et al. Preclinical immunogenicity study of trivalent meningococcal AWX-OMV vaccines for the African meningitis belt. *Vaccine* **2014**, *32*, 6631-6638, doi:10.1016/j.vaccine.2014.09.063.
166. Adamo, R.; Nilo, A.; Harfouche, C.; Brogioni, B.; Pecetta, S.; Brogioni, G.; Balducci, E.; Pinto, V.; Filippini, S.; Mori, E., et al. Investigating the immunodominance of carbohydrate antigens in a bivalent unimolecular glycoconjugate vaccine against serogroup A and C meningococcal disease. *Glycoconj J* **2014**, *31*, 637-647, doi:10.1007/s10719-014-9559-1.
167. Tunheim, G.; Arnemo, M.; Naess, L.M.; Norheim, G.; Garcia, L.; Cardoso, D.; Mandiarote, A.; Gonzalez, D.; Sinnadurai, K.; Fjeldheim Å, K., et al. Immune responses of a meningococcal A + W outer membrane vesicle (OMV) vaccine with and without aluminium hydroxide adjuvant in two different mouse strains. *Apmis* **2016**, *124*, 996-1003, doi:10.1111/apm.12589.
168. Acevedo, R.; Zayas, C.; Norheim, G.; Fernández, S.; Cedré, B.; Aranguren, Y.; Cuello, M.; Rodriguez, Y.; González, H.; Mandiarote, A., et al. Outer membrane vesicles extracted from Neisseria meningitidis serogroup X for prevention of meningococcal disease in Africa. *Pharmacol Res* **2017**, *121*, 194-201, doi:10.1016/j.phrs.2017.04.030.
169. Izeli Portilho, A.; Araujo Correa, V.; Dos Santos Cirqueira, C.; De Gaspari, E. Intranasal and Intramuscular Immunization with Outer Membrane Vesicles from Serogroup C Meningococci Induced Functional Antibodies and Immunologic Memory. *Immunol Invest* **2022**, *51*, 2066-2085, doi:10.1080/08820139.2022.2107931.
170. Koeberling, O.; Ispasanie, E.; Hauser, J.; Rossi, O.; Pluschke, G.; Caugant, D.A.; Saul, A.; MacLennan, C.A. A broadly-protective vaccine against meningococcal disease in sub-Saharan Africa based on generalized modules for membrane antigens (GMMA). *Vaccine* **2014**, *32*, 2688-2695, doi:10.1016/j.vaccine.2014.03.068.
171. Bidmos, F.A.; Chan, H.; Praekelt, U.; Tauseef, I.; Ali, Y.M.; Kaczmarski, E.B.; Feavers, I.; Bayliss, C.D. Investigation into the antigenic properties and contributions to growth in blood of the meningococcal haemoglobin receptors, HpuAB and HmbR. *PLoS One* **2015**, *10*, e0133855, doi:10.1371/journal.pone.0133855.

172. Hung, M.; Humbert, M.; Laver, J.; Phillips, R.; Heckels, J.; Christodoulides, M. A putative amino acid ABC transporter substrate-binding protein, NMB1612, from *Neisseria meningitidis,* induces murine bactericidal antibodies against meningococci expressing heterologous NMB1612 proteins. *Vaccine* **2015**, *33*, 4486-4494, doi:10.1016/j.vaccine.2015.07.032.
173. Norheim, G.; Sanders, H.; Mellesdal, J.W.; Sundfør, I.; Chan, H.; Brehony, C.; Vipond, C.; Dold, C.; Care, R.; Saleem, M., et al. An OMV Vaccine Derived from a Capsular Group B Meningococcus with Constitutive FetA Expression: Preclinical Evaluation of Immunogenicity and Toxicity. *PLoS One* **2015**, *10*, e0134353, doi:10.1371/journal.pone.0134353.
174. Sanders, H.; Norheim, G.; Chan, H.; Dold, C.; Vipond, C.; Derrick, J.P.; Pollard, A.J.; Maiden, M.C.; Feavers, I.M. FetA Antibodies Induced by an Outer Membrane Vesicle Vaccine Derived from a Serogroup B Meningococcal Isolate with Constitutive FetA Expression. *PLoS One* **2015**, *10*, e0140345, doi:10.1371/journal.pone.0140345.
175. Liao, G.; Zhou, Z.; Suryawanshi, S.; Mondal, M.A.; Guo, Z. Fully Synthetic Self-Adjuvanting alpha-2,9-Oligosialic Acid Based Conjugate Vaccines against Group C Meningitis. *ACS central science* **2016**, *2*, 210-218, doi:10.1021/acscentsci.5b00364.
176. Valentine, J.L.; Chen, L.; Perregaux, E.C.; Weyant, K.B.; Rosenthal, J.A.; Heiss, C.; Azadi, P.; Fisher, A.C.; Putnam, D.; Moe, G.R., et al. Immunization with Outer Membrane Vesicles displaying designer glycotopes yields class-switched, glycan-specific antibodies. *Cell chemical biology* **2016**, *23*, 655-665, doi:10.1016/j.chembiol.2016.05.014.
177. Humbert, M.; Hung, M.; Phillips, R.; Akoto, C.; Hill, A.; Tan, W.; Heckels, J.; Christodoulides, M. Vaccine potential and diversity of the putative Cell Binding Factor (CBF, NMB0345/NEIS1825) protein of *Neisseria meningitidis*. *PLoS One* **2016**, *11*, e0160403, doi:10.1371/journal.pone.0160403.
178. Wu, X.; Li, K.; Xie, M.; Yu, M.; Tang, S.; Li, Z.; Hu, S. Construction and protective immunogenicity of DNA vaccine pNMB0315 against Neisseria meningitidis serogroup B. *Mol Med Rep* **2018**, *17*, 3178-3185, doi:10.3892/mmr.2017.8255.
179. Li, Z.; Li, Y.; Wang, Y.; Hou, Y.; Cao, H.; Wu, X.; Hu, S.; Long, D. Intranasal immunization with a rNMB0315 and combination adjuvants induces protective immunity against Neisseria meningitidis serogroup B in mice. *Int Immunopharmacol* **2021**, *93*, 107411, doi:10.1016/j.intimp.2021.107411.
180. Dalal, J.; Rana, R.; Harale, K.; Hanif, S.; Kumar, N.; Singh, D.; Chhikara, M.K. Development and pre-clinical evaluation of a synthetic oligosaccharide-protein conjugate vaccine against Neisseria meningitidis serogroup C. *Vaccine* **2019**, *37*, 5297-5306, doi:10.1016/j.vaccine.2019.07.053.
181. Enotarpi, J.; Tontini, M.; Balocchi, C.; van der Es, D.; Auberger, L.; Balducci, E.; Carboni, F.; Proietti, D.; Casini, D.; Filippov, D.V., et al. A stabilized glycomimetic conjugate vaccine inducing protective antibodies against Neisseria meningitidis serogroup A. *Nat Commun* **2020**, *11*, 4434, doi:10.1038/s41467-020-18279-x.
182. Marsay, L.; Dold, C.; Paterson, G.K.; Yamaguchi, Y.; Derrick, J.P.; Chan, H.; Feavers, I.M.; Maiden, M.C.J.; Wyllie, D.; Hill, A.V., et al. Viral vectors expressing group B meningococcal outer membrane proteins induce strong antibody responses but fail to induce functional bactericidal activity. *J Infect* **2022**, *84*, 658-667, doi:10.1016/j.jinf.2022.02.032.
183. Soderstrom, C.; Braconier, J.H.; Kayhty, H.; Sjoholm, A.G.; Thuresson, B. Immune response to tetravalent meningococcal vaccine:opsonic and bactericidal functions of normal and properdin deficient sera. *Eur.J Clin Microbiol.Infect Dis* **1989**, *8 (3)*, 220-224.
184. Delvig, A.A.; Michaelsen, T.E.; Aase, A.; Hoiby, E.A.; Rosenqvist, E. Vaccine-induced IgG antibodies to the linear epitope on the PorB outer membrane protein promote opsonophagocytosis of Neisseria meningitidis by human neutrophils. *Clin Immunol Immunopathol* **1997**, *84*, 27-35, doi:10.1006/clin.1997.4360.
185. Martinez, J.; Pilishvili, T.; Barnard, S.; Caba, J.; Spear, W.; Romero-Steiner, S.; Carlone, G.M. Opsonophagocytosis of fluorescent polystyrene beads coupled to Neisseria meningitidis

serogroup A, C, Y, or W135 polysaccharide correlates with serum bactericidal activity. *Clin Diagn Lab Immunol* **2002**, *9*, 485-488, doi:10.1128/cdli.9.2.485-488.2002.
186. Cheng, W.C.; Webb, E.; Vedros, N.; Ng, J. Fractionation and characterization of surface antigens from group A Neisseria meningitidis. *J Immunol* **1975**, *114*, 1497-1505.
187. Craven, D.E.; Frasch, C.E. Protection against group B meningococcal disease: evaluation of serotype 2 protein vaccines in a mouse bacteremia model. *Infect.Immun.* **1979**, *26*, 110-117.
188. Quakyi, E.K.; Frasch, C.E.; Buller, N.; Chao-Ming, T. Immunization with meningococcal outer-membrane protein vesicles containing lipooligosaccharide protects mice against lethal experimental Group B *Neisseria meningitidis* infection and septic shock. *J.Infect.Dis.* **1999**, *180*, 747-754.
189. West, D.; Reddin, K.; Matheson, M.; Heath, R.; Funnell, S.; Hudson, M.; Robinson, A.; Gorringe, A. Recombinant *Neisseria meningitidis* transferrin binding protein A protects against experimental meningococcal infection. *Infect Immun* **2001**, *69*, 1561-1567, doi:10.1128/iai.69.3.1561-1567.2001.
190. Reddin, K.M.; Crowley-Luke, A.; Clark, S.O.; Vincent, P.J.; Gorringe, A.R.; Hudson, M.J.; Robinson, A. Bordetella pertussis fimbriae are effective carrier proteins in Neisseria meningitidis serogroup C conjugate vaccines. *FEMS Immunol Med Microbiol* **2001**, *31*, 153-162, doi:10.1111/j.1574-695X.2001.tb00512.x.
191. Newcombe, J.; Eales-Reynolds, L.J.; Wootton, L.; Gorringe, A.R.; Funnell, S.G.; Taylor, S.C.; McFadden, J.J. Infection with an avirulent phoP mutant of *Neisseria meningitidis* confers broad cross-reactive immunity. *Infect Immun* **2004**, *72*, 338-344.
192. Li, Y.; Sun, Y.H.; Ison, C.; Levine, M.M.; Tang, C.M. Vaccination with attenuated *Neisseria meningitidis strains* protects against challenge with live meningococci. *Infect Immun* **2004**, *72*, 345-351.
193. Beninati, C.; Arseni, S.; Mancuso, G.; Magliani, W.; Conti, S.; Midiri, A.; Biondo, C.; Polonelli, L.; Teti, G. Protective immunization against group B meningococci using anti-idiotypic mimics of the capsular polysaccharide. *J Immunol* **2004**, *172*, 2461-2468, doi:10.4049/jimmunol.172.4.2461.
194. Beninati, C.; Midiri, A.; Mancuso, G.; Biondo, C.; Arigo, M.; Gerace, E.; Papasergi, S.; Gambuzza, M.; Boretti, M.; Magliani, W., et al. Antiidiotypic DNA vaccination induces serum bactericidal activity and protection against group B meningococci. *J Exp Med* **2006**, *203*, 111-118, doi:10.1084/jem.20051540.
195. Jessouroun, E.; da Silveira, I.F.; Larangeira, A.P.; Pereira, S.; Fernandes, S.A.; Rabinovitch, L.; Frasch, C.E.; Castro-Faria-Neto, H.C.; Bozza, P.T. Outer membrane vesicles (OMVs) and detoxified lipooligosaccharide (dLOS) obtained from Brazilian prevalent N. meningitidis serogroup B strains protect mice against homologous and heterologous meningococcal infection and septic shock

1. *Vaccine* **2004**, *22*, 2617-2625.
196. Zhu, D.; Zhang, Y.; Barniak, V.; Bernfield, L.; Howell, A.; Zlotnick, G. Evaluation of recombinant lipidated P2086 protein as a vaccine candidate for group B Neisseria meningitidis in a murine nasal challenge model. *Infect Immun* **2005**, *73*, 6838-6845, doi:10.1128/iai.73.10.6838-6845.2005.
197. Zarantonelli, M.L.; Antignac, A.; Lancellotti, M.; Guiyoule, A.; Alonso, J.M.; Taha, M.K. Immunogenicity of meningococcal PBP2 during natural infection and protective activity of anti-PBP2 antibodies against meningococcal bacteraemia in mice. *J Antimicrob Chemother* **2006**, *57*, 924-930, doi:10.1093/jac/dkl066.
198. Li, Y.; Wooldridge, K.G.; Javed, M.A.; Tang, C.M.; Ala'aldeen, D.A. Secreted proteins of Neisseria meningitidis protect mice against infection. *Vaccine* **2009**, *27*, 2320-2325, doi:10.1016/j.vaccine.2009.02.034.
199. Cywes-Bentley, C.; Skurnik, D.; Zaidi, T.; Roux, D.; Deoliveira, R.B.; Garrett, W.S.; Lu, X.; O'Malley, J.; Kinzel, K.; Zaidi, T., et al. Antibody to a conserved antigenic target is protective

against diverse prokaryotic and eukaryotic pathogens. *Proc Natl Acad Sci U S A* **2013**, *110*, E2209-2218, doi:10.1073/pnas.1303573110.
200. Kita, E.; Kashiba, S. Analysis of immune responses in genital tracts of mice immunised with purified ribosomal fractions of Neisseria gonorrhoeae. *British Journal Of Venereal Diseases* **1984**, *60*, 219-225.
201. Cremieux, A.C.; Puissant, A.; Ancelle, R.; Martin, P.M. Bactericidal antibodies against *Neisseria gonorrhoeae* elicited by *Neisseria meningitidis*. *Lancet* **1984**, *2*, 930, doi:S0140-6736(84)90687-1 [pii].
202. Poolman, J.T.; Buchanan, T.M. Monoclonal-antibody activity against native and denatured forms of gonococcal outer-membrane proteins as detected within ultrathin, longitudinal slices of polyacrylamide gels. *J.Immunol.Meth.* **1984**, *75*, 265-274.
203. Virji, M.; Heckels, J.E. Role of anti-pilus antibodies in host defense against gonococcal- infection studied with monoclonal anti-pilus antibodies. *Infect.Immun.* **1985**, *49*, 621-628.
204. Virji, M.; Zak, K.; Fletcher, J.N.; Heckels, J.E. The protective effect of monoclonal antibodies directed against gonococcal outer membrane protein IB. In *Protein-carbohydrate interactions in biological systems.*, Lark, D.L., Ed. Academic Press: London, 1986; pp. 381-382.
205. Virji, M.; Fletcher, J.N.; Zak, K.; Heckels, J.E. The potential protective effect of monoclonal antibodies to gonococcal outer membrane protein IA. *J.Gen.Microbiol.* **1987**, *133*, 2639-2646.
206. Heckels, J.E.; Virji, M.; Zak, K.; Fletcher, J.N. Immunobiology of gonococcal outer-membrane protein-i. *Antonie Van Leeuwenhoek J.Microbiol* **1987**, *53*, 461-464.
207. Butt, N.J.; Virji, M.; Vayreda, F.; Lambden, P.R.; Heckels, J.E. Gonococcal outer membrane protein PIB comparative sequence analysis and localization of epitopes which are recognized by type-specific and cross-reacting monoclonal antibodies. *J.Gen.Microbiol.* **1990**, *136*, 2165-2172.
208. Zhu, W.; Thomas, C.E.; Chen, C.J.; Van Dam, C.N.; Johnston, R.E.; Davis, N.L.; Sparling, P.F. Comparison of immune responses to gonococcal PorB delivered as outer membrane vesicles, recombinant protein, or Venezuelan equine encephalitis virus replicon particles. *Infect Immun* **2005**, *73*, 7558-7568, doi:10.1128/iai.73.11.7558-7568.2005.
209. Wetzler, L.M.; Blake, M.S.; Barry, K.; Gotschlich, E.C. Gonococcal porin vaccine evaluation - comparison of por proteosomes, liposomes, and blebs isolated from rmp deletion mutants. *J Infect Dis* **1992**, *166*, 551-555.
210. Wetzler, L.M.; Blake, M.S.; Gotschlich, E.C. Characterization and specificity of antibodies to protein I of *Neisseria gonorrhoeae* produced by injection with various protein I - adjuvant preparations. *J Exp Med* **1988**, *168*, 1883-1897.
211. Wetzler, L.M.; Blake, M.S.; Gotschlich, E.C. Characterization of antibodies raised to protein i (pi) of neisseria gonorrhoeae by injection with pi-liposome constructs. *In Gonococci and Meningococci.pp457-463.Edited by J.T.Poolman et al.,Kluwer Academic Publishers,,Dordrecht,Netherlands* **1988**.
212. Wetzler, L.M.; Blake, M.S.; Gotschlich, E.C. Protein 1 (Por) of *Neisseria gonorrhoeae* as an immunogen: liposomes, proteosomes, and the lack of blocking antibodies. *Trans Am Assoc Phys* **1989**, 78-90.
213. Elkins, C.; Barkley, K.B.; Carbonetti, N.H.; Coimbre, A.J.; Sparling, P.F. Immunobiology of purified recombinant outer membrane porin protein I of *Neisseria gonorrhoeae*. *Mol Microbiol* **1994**, *14*, 1059-1075, doi:10.1111/j.1365-2958.1994.tb01339.x.
214. Heckels, J.E.; Virji, M.; Tinsley, C.R. Vaccination against gonorrheoa: the potential protective effect of immunization with a synthetic peptide containing a conserved epitope of gonococcal outer membrane protein IB. *Vaccine* **1990**, *8*, 225-230.
215. Elkins, C.; Carbonetti, N.H.; Varela, V.A.; Stirewalt, D.; Klapper, D.G.; Sparling, P.F. Antibodies to N-terminal peptides of gonococcal porin are bactericidal when gonococcal lipopolysaccharide is not sialylated. *Mol Microbiol* **1992**, *6*, 2617-2628.

216. Virji, M.; Zak, K.; Heckels, J.E. Outer-membrane protein-iii of *Neisseria-gonorrhoeae* - variations in biological properties of antibodies directed against different epitopes. *J.Gen.Microbiol.* **1987**, *133*, 3393-3401.
217. Brossay, L.; Paradis, G.; Pepin, A.; Mourad, W.; Cote, L.; Hebert, J. Idiotype and anti-anti-idiotype antibodies to Neisseria gonorrhoeae lipooligosaccharides with bactericidal activity but no cross-reactivity with red blood cell antigens. *J.Immunol.* **1993**, *151*, 234-243.
218. Gulati, S.; McQuillen, D.P.; Sharon, J.; Rice, P.A. Experimental immunization with a monoclonal anti-idiotope antibody that mimics the *Neisseria gonorrhoeae* lipooligosaccharide epitope 2C7. *J Infect Dis* **1996**, *174*, 1238-1248, doi:10.1093/infdis/174.6.1238.
219. Gulati, S.; Beurskens, F.J.; de Kreuk, B.J.; Roza, M.; Zheng, B.; DeOliveira, R.B.; Shaughnessy, J.; Nowak, N.A.; Taylor, R.P.; Botto, M., et al. Complement alone drives efficacy of a chimeric antigonococcal monoclonal antibody. *PLoS Biol* **2019**, *17*, e3000323, doi:10.1371/journal.pbio.3000323.
220. Ngampasutadol, J.; Rice, P.A.; Walsh, M.T.; Gulati, S. Characterization of a peptide vaccine candidate mimicking an oligosaccharide epitope of *Neisseria gonorrhoeae* and resultant immune responses and function. *Vaccine* **2006**, *24*, 157-170, doi:10.1016/j.vaccine.2005.07.065.
221. Gulati, S.; Zheng, B.; Reed, G.W.; Su, X.; Cox, A.D.; St, M.F.; Stupak, J.; Lewis, L.A.; Ram, S.; Rice, P.A. Immunization against a saccharide epitope accelerates clearance of experimental gonococcal infection. *PLoS Pathog.* **2013**, *9*, e1003559, doi:10.1371/journal.ppat.1003559 [doi];PPATHOGENS-D-13-00716 [pii].
222. Gulati, S.; Pennington, M.W.; Czerwinski, A.; Carter, D.; Zheng, B.; Nowak, N.A.; DeOliveira, R.B.; Shaughnessy, J.; Reed, G.W.; Ram, S., et al. Preclinical Efficacy of a Lipooligosaccharide Peptide Mimic Candidate Gonococcal Vaccine. *mBio* **2019**, *10*, pii: e02552-02519, doi:10.1128/mBio.02552-19.
223. Chakraborti, S.; Gulati, S.; Zheng, B.; Beurskens, F.J.; Schuurman, J.; Rice, P.A.; Ram, S. Bypassing Phase Variation of Lipooligosaccharide (LOS): Using Heptose 1 Glycan Mutants To Establish Widespread Efficacy of Gonococcal Anti-LOS Monoclonal Antibody 2C7. *Infect Immun* **2020**, *88*, doi:10.1128/iai.00862-19.
224. Parzych, E.M.; Gulati, S.; Zheng, B.; Bah, M.A.; Elliott, S.T.C.; Chu, J.D.; Nowak, N.; Reed, G.W.; Beurskens, F.J.; Schuurman, J., et al. Synthetic DNA Delivery of an Optimized and Engineered Monoclonal Antibody Provides Rapid and Prolonged Protection against Experimental Gonococcal Infection. *mBio* **2021**, *12*, doi:10.1128/mBio.00242-21.
225. Price, G.A.; Russell, M.W.; Cornelissen, C.N. Intranasal administration of recombinant *Neisseria gonorrhoeae* transferrin binding proteins A and B conjugated to the cholera toxin B subunit induces systemic and vaginal antibodies in mice. *Infect Immun* **2005**, *73*, 3945-3953, doi:10.1128/IAI.73.7.3945-3953.2005.
226. Price, G.A.; Masri, H.P.; Hollander, A.M.; Russell, M.W.; Cornelissen, C.N. Gonococcal transferrin binding protein chimeras induce bactericidal and growth inhibitory antibodies in mice. *Vaccine* **2007**, *25*, 7247-7260, doi:10.1016/j.vaccine.2007.07.038.
227. Cole, J.G.; Jerse, A.E. Functional characterization of antibodies against *Neisseria gonorrhoeae* opacity protein loops. *PLoS One* **2009**, *4*, e8108, doi:10.1371/journal.pone.0008108.
228. Li, G.; Jiao, H.; Jiang, G.; Wang, J.; Zhu, L.; Xie, R.; Yan, H.; Chen, H.; Ji, M. *Neisseria gonorrhoeae* NspA induces specific bactericidal and opsonic antibodies in mice. *Clin Vacc Immunol* **2011**, *18*, 1817-1822, doi:10.1128/CVI.05245-11.
229. Rippa, V.; Santini, L.; Lo Surdo, P.; Cantini, F.; Veggi, D.; Gentile, M.A.; Grassi, E.; Iannello, G.; Brunelli, B.; Ferlicca, F., et al. Molecular Engineering of Ghfp, the Gonococcal Orthologue of Neisseria meningitidis Factor H Binding Protein. *Clin Vaccine Immunol* **2015**, *22*, 769-777, doi:10.1128/cvi.00794-14.
230. Humbert, M.V.; Christodoulides, M. Immunization with recombinant truncated *Neisseria meningitidis*-Macrophage Infectivity Potentiator (rT-Nm-MIP) protein induces murine

antibodies that are cross-reactive and bactericidal for *Neisseria gonorrhoeae*. *Vaccine* **2018**, *10.1016/j.vaccine.2018.05.069*.
231. Jiao, H.; Yang, H.; Zhao, D.; Chen, J.; Zhang, Q.; Liang, J.; Yin, Y.; Kong, G.; Li, G. Design and immune characterization of a novel Neisseria gonorrhoeae DNA vaccine using bacterial ghosts as vector and adjuvant. *Vaccine* **2018**, *36*, 4532-4539, doi:10.1016/j.vaccine.2018.06.006.
232. Wang, S.; Xue, J.; Lu, P.; Ni, C.; Cheng, H.; Han, R.; van der Veen, S. Gonococcal MtrE and its surface-expressed Loop 2 are immunogenic and elicit bactericidal antibodies. *J Infect* **2018**, *77*, 191-204, doi:10.1016/j.jinf.2018.06.001.
233. Almonacid-Mendoza, H.L.; Humbert, M.V.; Dijokaite, A.; Cleary, D.W.; Soo, Y.; Hung, M.C.; Orr, C.M.; Machelett, M.M.; Tews, I.; Christodoulides, M. Structure of the Recombinant *Neisseria gonorrhoeae* Adhesin Complex Protein (rNg-ACP) and Generation of Murine Antibodies with Bactericidal Activity against Gonococci. *mSphere* **2018**, *3*, doi:10.1128/mSphere.00331-18.
234. Jen, F.E.; Semchenko, E.A.; Day, C.J.; Seib, K.L.; Jennings, M.P. The Neisseria gonorrhoeae Methionine Sulfoxide Reductase (MsrA/B) Is a Surface Exposed, Immunogenic, Vaccine Candidate. *Front Immunol* **2019**, *10*, 137, doi:10.3389/fimmu.2019.00137.
235. Beernink, P.T.; Vianzon, V.; Lewis, L.A.; Moe, G.R.; Granoff, D.M. A Meningococcal Outer Membrane Vesicle Vaccine with Overexpressed Mutant FHbp Elicits Higher Protective Antibody Responses in Infant Rhesus Macaques than a Licensed Serogroup B Vaccine. *mBio* **2019**, *10*, doi:10.1128/mBio.01231-19.
236. Zhu, T.; McClure, R.; Harrison, O.B.; Genco, C.; Massari, P. Integrated Bioinformatic Analyses and Immune Characterization of New *Neisseria gonorrhoeae* Vaccine Antigens Expressed during Natural Mucosal Infection. *Vaccines* **2019**, *7*, doi:10.3390/vaccines7040153.
237. Clow, F.; O'Hanlon, C.J.; Christodoulides, M.; Radcliff, F.J. Feasibility of Using a Luminescence-Based Method to Determine Serum Bactericidal Activity against Neisseria gonorrhoeae. *Vaccines* **2019**, *7*, doi:10.3390/vaccines7040191.
238. Francis, I.P.; Lui, X.; Wetzler, L.M. Isolation of Naturally Released Gonococcal Outer Membrane Vesicles as Vaccine Antigens. In *Neisseria gonorrhoeae: methods and protocols*, 2019/05/24 ed.; Christodoulides, M., Ed. Springer/Humana Press: New York, 2019; 10.1007/978-1-4939-9496-0_9.
239. Semchenko, E.A.; Day, C.J.; Seib, K.L. The *Neisseria gonorrhoeae* Vaccine Candidate NHBA Elicits Antibodies That Are Bactericidal, Opsonophagocytic and That Reduce Gonococcal Adherence to Epithelial Cells. *Vaccines* **2020**, *8*, doi:10.3390/vaccines8020219.
240. Semchenko, E.A.; Tan, A.; Borrow, R.; Seib, K.L. The Serogroup B Meningococcal Vaccine Bexsero Elicits Antibodies to Neisseria gonorrhoeae. *Clin Infect Dis* **2019**, *69*, 1101-1111, doi:10.1093/cid/ciy1061.
241. Leduc, I.; Connolly, K.L.; Begum, A.; Underwood, K.; Darnell, S.; Shafer, W.M.; Balthazar, J.T.; Macintyre, A.N.; Sempowski, G.D.; Duncan, J.A., et al. The serogroup B meningococcal outer membrane vesicle-based vaccine 4CMenB induces cross-species protection against *Neisseria gonorrhoeae*. *PLoS Pathog* **2020**, *16*, e1008602, doi:10.1371/journal.ppat.1008602.
242. Piekarowicz, A.; Klyz, A.; Adamczyk-Poplawska, M.; Stein, D.C. Association of host proteins with the broad host range filamentous phage NgoPhi6 of *Neisseria gonorrhoeae*. *PLoS One* **2020**, *15*, e0240579, doi:10.1371/journal.pone.0240579.
243. Semchenko, E.A.; Day, C.J.; Seib, K.L. MetQ of *Neisseria gonorrhoeae* Is a Surface-Expressed Antigen That Elicits Bactericidal and Functional Blocking Antibodies. *Infect Immun* **2017**, *85*, e00898-00816, doi:10.1128/IAI.00898-16.
244. Li, G.; Xie, R.; Zhu, X.; Mao, Y.; Liu, S.; Jiao, H.; Yan, H.; Xiong, K.; Ji, M. Antibodies with higher bactericidal activity induced by a Neisseria gonorrhoeae Rmp deletion mutant strain. *PLoS One* **2014**, *9*, e90525, doi:10.1371/journal.pone.0090525.

245. Cardinale, J.A.; Clark, V.L. Expression of AniA, the major anaerobically induced outer membrane protein of Neisseria gonorrhoeae, provides protection against killing by normal human sera. *Infect Immun* **2000**, *68*, 4368-4369, doi:10.1128/iai.68.7.4368-4369.2000.
246. Zhu, W.; Thomas, C.E.; Sparling, P.F. DNA immunization of mice with a plasmid encoding *Neisseria gonorrhea* PorB protein by intramuscular injection and epidermal particle bombardment. *Vaccine* **2004**, *22*, 660-669.
247. Arko, R.J.; Bullard, J.C.; Duncan, W.P. Effects of laboratory maintenance on the nature of surface reactive antigens of Neisseria gonorrhoeae. *Br J Vener Dis* **1976**, *52*, 316-325, doi:10.1136/sti.52.5.316.
248. Buchanan, T.M.; Pearce, W.A.; Schoolnik, G.K.; Arko, R.J. Session iv. genitourinary tract disease in humans: etiologic agents and antigens. protection against infection with neisseria gonorrhoeae by immunization with outer membrane complex and purified pili. *J.Infect.Dis.* **1977**, *136*, S132-S137.
249. Frasch, C.E.; Robbins, J.D. Protection against group B meningococcal disease III. Immunogenicity of serotype 2 vaccines and specificity of protection in a guinea pig model. *J.Exp.Med.* **1978**, *147*, 629-644.
250. Cooper, M.D.; Tewari, R.P.; Bowser, D.V. Immunogenicity of ribosomal preparations from Neisseria gonorrhoeae. *Infect Immun* **1980**, *28*, 92-100, doi:10.1128/iai.28.1.92-100.1980.
251. Peppler, M.S.; Frasch, C.E. Protection against group B Neisseria meningitidis disease: effect of serogroup B polysaccharide and polymyxin B on immunogenicity of serotype protein preparations. *Infect.Immun.* **1982**, *37*, 264-270.
252. Porro, M.; Costantino, P.; Giovannoni, F.; Pellegrini, V.; Tagliaferri, L.; Vannozzi, F.; Viti, S. A molecular model of artificial glycoprotein with predetermined multiple immunodeterminants for gram-positive and gram-negative encapsulated bacteria. *Molecular immunology* **1986**, *23*, 385-391, doi:10.1016/0161-5890(86)90136-7.
253. Senior, K.E.; Demarco de Hormaeche, R.; Jessop, H.L. Immunization of guinea pigs with epitope C-rich lipopolysaccharide from Neisseria gonorrhoea; in vivo selection of gonococcal variants. *Microb Pathog* **1989**, *6*, 251-264, doi:10.1016/0882-4010(89)90099-5.
254. Fusco, P.C.; Michon, F.; Laude-Sharp, M.; Minetti, C.A.S.A.; Huang, C.H.; Heron, I.; Blake, M.S. Preclinical studies on a recombinant group B meningococcal porin as a carrier for a novel Haemophilus influenzae type b conjugate vaccine. *Vaccine* **1998**, *16*, 1842-1849.
255. Veale, D.R.; Smith, H.; Witt, K.A.; Marshall, R.B. Differential ability of colonial types of Neisseria gonorrhoeae to produce infection cutaneous perforated plastic chambers in guinea-pigs and rabbits. *J Med Microbiol* **1975**, *8*, 325-335, doi:10.1099/00222615-8-2-325.
256. Turner, W.H.; Novotny, P. The inability of Neisseria gonorrhoeae pili antibodies to confer immunity in subcutaneous guinea-pig chambers. *J Gen Microbiol* **1976**, *92*, 224-228, doi:10.1099/00221287-92-1-224.
257. Penn, C.W.; Sen, D.; Veale, D.R.; Parsons, N.J.; Smith, H. Morphological, biological and antigenic properties of Neisseria gonorrhoeae adapted to growth in guinea-pig subcutaneous chambers. *J Gen Microbiol* **1976**, *97*, 35-43, doi:10.1099/00221287-97-1-35.
258. Wong, K.H.; Arko, R.J.; Logan, L.C.; Bullard, J.C. Immunological and serological diversity of Neisseria gonorrhoeae: gonococcal serotypes and their relationship with immunotypes. *Infect Immun* **1976**, *14*, 1297-1301, doi:10.1128/iai.14.6.1297-1301.1976.
259. Jacobs, N.F., Jr.; Kraus, S.J.; Thornsberry, C.; Bullard, J. Isolation and characterization of a rough colony type of Neisseria gonorrhoeae. *J Clin Microbiol* **1977**, *5*, 365-369, doi:10.1128/jcm.5.3.365-369.1977.
260. Penn, C.W.; Parsons, N.J.; Sen, D.; Veale, D.R.; Smith, H. Immunization of guinea pigs with Neisseria gonorrhoeae: strain specificity and mechanisms of immunity. *J Gen Microbiol* **1977**, *100*, 159-166, doi:10.1099/00221287-100-1-159.

261. Arko, R.J.; Wong, K.H. Comparative physical and immunological aspects of the chimpanzee and guinea-pig subcutaneous chamber models of Neisseria gonorrhoeae infection. *Br J Vener Dis* **1977**, *53*, 101-105, doi:10.1136/sti.53.2.101.
262. Rittenberg, S.C.; Penn, C.W.; Parsons, N.J.; Veale, D.R.; Smith, H. Phenotypic changes in the resistance of Neisseria gonorrhoeae to killing by normal human serum. *J Gen Microbiol* **1977**, *103*, 69-75, doi:10.1099/00221287-103-1-69.
263. Novotny, P.; Broughton, E.S.; Cownley, K.; Hughes, M.; Turner, W.H. Strain related infectivity of Neisseria gonorrhoeae for the guinea-pig subcutaneous chamber and the variability of the immune resistance in different breeds of guinea-pig. *Br J Vener Dis* **1978**, *54*, 88-96, doi:10.1136/sti.54.2.88.
264. Parsons, N.J.; Penn, C.W.; Veale, D.R.; Smith, H. More than one antigen contributes to the immunogenicity of Neisseria gonorrhoeae in the guinea pig chamber model. *J Gen Microbiol* **1979**, *113*, 97-104, doi:10.1099/00221287-113-1-97.
265. Goldner, M.; Penn, C.W.; Sanyal, S.C.; Veale, D.R.; Smith, H. Phenotypically determined resistance of Neisseria gonorrhoeae to normal human serum: environmental factors in subcutaneous chambers in guinea pigs. *J Gen Microbiol* **1979**, *114*, 169-177, doi:10.1099/00221287-114-1-169.
266. Parsons, N.J.; Penn, C.W.; Veale, D.R.; Smith, H. The complexity of immunogenicity of Neisseria gonorrhoeae in the guinea pig subcutaneous chamber model. *J Gen Microbiol* **1980**, *118*, 523-527, doi:10.1099/00221287-118-2-523.
267. Mcbride, H.M.; Lambden, P.R.; Heckels, J.E.; Watt, P.J. The role of outer-membrane proteins in the survival of neisseria- gonorrhoeae-p9 within guinea-pig subcutaneous chambers. *J.Gen.Microbiol.* **1981**, *126*, 63-67.
268. Wannemuehler, M.J.; Miller, R.D.; Cooper, M.D. Characterization of the immune response in subcutaneous chambers of guinea pigs immunized with a ribosomal preparation from Neisseria gonorrhoeae. *Infect Immun* **1982**, *37*, 469-473, doi:10.1128/iai.37.2.469-473.1982.
269. Lambden, P.R.; Heckels, J.E.; Watt, P.J. Effect of anti-pilus antibodies on survival of gonococci within guinea-pig subcutaneous chambers. *Infect.Immun.* **1982**, *38*, 27-30.
270. Frenette, M.; Beaudet, R.; Bisaillon, J.G.; Sylvestre, M.; Portelance, V. Chemical and biological characterization of a gonococcal growth inhibitor produced by Staphylococcus haemolyticus isolated from urogenital flora. *Infect Immun* **1984**, *46*, 340-345, doi:10.1128/iai.46.2.340-345.1984.
271. Demarco de Hormaeche, R.; Thornley, M.J.; Glauert, A.M. Demonstration by light and electron microscopy of capsules on gonococci recently grown in vivo. *J Gen Microbiol* **1978**, *106*, 81-91, doi:10.1099/00221287-106-1-81.
272. Demarco de Hormaeche, R.; Jessop, H.; Senior, K. Gonococcal variants selected by growth in vivo or in vitro have antigenically different LPS. *Microb Pathog* **1988**, *4*, 289-297, doi:10.1016/0882-4010(88)90089-7.
273. Demarco de Hormaeche, R.; Macpherson, A.; Bowe, F.; Hormaeche, C.E. Alterations of the LPS determine virulence of Neisseria gonorrhoeae in guinea-pig subcutaneous chambers. *Microb Pathog* **1991**, *11*, 159-170, doi:10.1016/0882-4010(91)90046-d.
274. Chamberlain, L.M.; Strugnell, R.; Dougan, G.; Hormaeche, C.E.; Demarco de Hormaeche. Neisseria gonorrhoeae strain MS11 harbouring a mutation in gene aroA isattenuated and immunogenic. *Microb.Path.* **1993**, *15*, 51-63.
275. Jennings, H.J.; Lugowski, C.; Ashton, F.E. Conjugation of meningococcal lipopolysaccharide r-type oligosaccharides to tetanus toxoid as a route to a potential vaccine against group B Neisseria meningitidis. *Infect.Immun.* **1984**, *43*, 407-441.
276. Christodoulides, M.; Heckels, J.E. Immunization with a multiple antigen peptide containing defined B- and T-cell epitopes: production of bactericidal antibodies against group B *Neisseria meningitidis*. *Microbiology (Reading, England)* **1994**, *140*, 2951-2960, doi:10.1099/13500872-140-11-2951.

277. Mistretta, N.; Guy, B.; Berard, Y.; Dalencon, F.; Fratantonio, O.; Gregoire, C.; Lechevallier, A.; Lheritier, P.; Revet, L.; Moreau, M., et al. Improvement of immunogenicity of meningococcal lipooligosaccharide by coformulation with lipidated transferrin-binding protein B in liposomes: implications for vaccine development. *Clin Vaccine Immunol* **2012**, *19*, 711-722, doi:10.1128/cvi.05683-11.
278. Bash, M.C.; Lynn, F.; Concepcion, N.F.; Tappero, J.W.; Carlone, G.M.; Frasch, C.E. Genetic and immunologic characterization of a novel serotype 4, 15 strain of Neisseria meningitidis. *FEMS Immunol Med Microbiol* **2000**, *29*, 169-176, doi:10.1111/j.1574-695X.2000.tb01519.x.
279. St Michael, F.; Cairns, C.M.; Filion, A.L.; Biolchi, A.; Brunelli, B.; Giuliani, M.; Richards, J.C.; Cox, A.D. Investigating the candidacy of LPS-based glycoconjugates to prevent invasive meningococcal disease: conjugates based on core oligosaccharides. *Glycoconj J* **2014**, *31*, 25-39, doi:10.1007/s10719-013-9500-z.
280. Jiang, H.Q.; Hoiseth, S.K.; Harris, S.L.; McNeil, L.K.; Zhu, D.; Tan, C.; Scott, A.A.; Alexander, K.; Mason, K.; Miller, L., et al. Broad vaccine coverage predicted for a bivalent recombinant factor H binding protein based vaccine to prevent serogroup B meningococcal disease. *Vaccine* **2010**, *28*, 6086-6093, doi:10.1016/j.vaccine.2010.06.083.
281. Harris, S.L.; Zhu, D.; Murphy, E.; McNeil, L.K.; Wang, X.; Mayer, L.W.; Harrison, L.H.; Jansen, K.U.; Anderson, A.S. Preclinical evidence for the potential of a bivalent fHBP vaccine to prevent Neisseria meningitidis Serogroup C Disease. *Hum Vaccin* **2011**, *7 Suppl*, 68-74, doi:10.4161/hv.7.0.14564.
282. Granoff, D.M.; Ram, S.; Beernink, P.T. Does binding of complement factor H to the meningococcal vaccine antigen, factor H binding protein, decrease protective serum antibody responses? *Clin Vaccine Immunol* **2013**, *20*, 1099-1107, doi:10.1128/cvi.00260-13.
283. Moran, E.E.; Burden, R.; Labrie, J.E., 3rd; Wen, Z.; Wang, X.M.; Zollinger, W.D.; Zhang, L.; Pinto, V.B. Analysis of the bactericidal response to an experimental Neisseria meningitidis vesicle vaccine. *Clin Vaccine Immunol* **2012**, *19*, 659-665, doi:10.1128/cvi.00070-12.
284. Matthias, K.A.; Reveille, A.; Connolly, K.L.; Jerse, A.E.; Gao, Y.S.; Bash, M.C. Deletion of major porins from meningococcal outer membrane vesicle vaccines enhances reactivity against heterologous serogroup B Neisseria meningitidis strains. *Vaccine* **2020**, *38*, 2396-2405, doi:10.1016/j.vaccine.2020.01.038.
285. Gill, M.J.; McQuillen, D.P.; van Putten, J.P.; Wetzler, L.M.; Bramley, J.; Crooke, H.; Parsons, N.J.; Cole, J.A.; Smith, H. Functional characterization of a sialyltransferase-deficient mutant of Neisseria gonorrhoeae. *Infect Immun* **1996**, *64*, 3374-3378, doi:10.1128/iai.64.8.3374-3378.1996.
286. Gulati, S.; Ngampasutadol, J.; Yamasaki, R.; McQuillen, D.P.; Rice, P.A. Strategies for mimicking Neisserial saccharide epitopes as vaccines. *International reviews of immunology* **2001**, *20*, 229-250, doi:10.3109/08830180109043036.
287. Piekarowicz, A.; Kłyż, A.; Majchrzak, M.; Stein, D.C. Oral Immunization of Rabbits with S. enterica Typhimurium Expressing Neisseria gonorrhoeae Filamentous Phage Φ6 Induces Bactericidal Antibodies Against N. gonorrhoeae. *Sci Rep* **2016**, *6*, 22549, doi:10.1038/srep22549.
288. Kłyż, A.; Piekarowicz, A. Phage proteins are expressed on the surface of Neisseria gonorrhoeae and are potential vaccine candidates. *PLoS One* **2018**, *13*, e0202437, doi:10.1371/journal.pone.0202437.
289. Saukkonen, K.; Abdillahi, H.; Poolman, J.T.; Leinonen, M. Protective efficacy of monoclonal antibodies to class 1 and class 3 outer membrane proteins of Neisseria meningitidis B:15:P1.16 in infant rat infection model - new prospects for vaccine development. *Microb.Path.* **1987**, *3*, 261-267.
290. Toropainen, M.; Saarinen, L.; van der Ley, P.; Kuipers, B.; Käyhty, H. Murine monoclonal antibodies to PorA of Neisseria meningitidis show reduced protective activity in vivo against B:15:P1.7,16 subtype variants in an infant rat infection model. *Microb Pathog* **2001**, *30*, 139-148, doi:10.1006/mpat.2000.0419.

291. Saukkonen, K.; Leinonen, M.; Kayhty, H.; Abdillahi, H.; Poolman, J.T. Monoclonal antibodies to the rough lipopolysaccharide of Neisseria meningitidis protect infant rats from meningococcal infection. *J.Infect.Dis.* **1988**, *158*, 209-212.
292. Saukkonen, K.; Leinonen, M.; Abdillahi, H.; Poolman, J.T. Comparative evaluation of potential components for group B meningococcal vaccine by passive protection in the infant rat and *in vitro* bactericidal assay. *Vaccine* **1989**, *7*, 325-328.
293. Plested, J.S.; Harris, S.L.; Wright, J.C.; Coull, P.A.; Makepeace, K.; Gidney, M.A.; Brisson, J.R.; Richards, J.C.; Granoff, D.M.; Moxon, E.R. Highly conserved *Neisseria meningitidis* inner-core lipopolysaccharide epitope confers protection against experimental meningococcal bacteremia. *J Infect Dis* **2003**, *187*, 1223-1234, doi:10.1086/368360.
294. Saukkonen, K.; Haltia, M.; Frosch, M.; Bitter-Süerman, D.; Leinonen, M. Antibodies to the capsular polysaccharide of Neisseria meningitidis group B or E. coli K1 bind to the brains of infant rats in vitro but not in vivo. *Microb Pathog* **1986**, *1*, 101-105, doi:10.1016/0882-4010(86)90036-7.
295. Schmidt, S.; Zhu, D.; Barniak, V.; Mason, K.; Zhang, Y.; Arumugham, R.; Metcalf, T. Passive immunization with Neisseria meningitidis PorA specific immune sera reduces nasopharyngeal colonization of group B meningococcus in an infant rat nasal challenge model. *Vaccine* **2001**, *19*, 4851-4858, doi:10.1016/s0264-410x(01)00229-8.
296. Yero, C.D.; Pajón, F.R.; Caballero, M.E.; Cobas, A.K.; López, H.Y.; Fariñas, M.M.; Gonzáles, B.S.; Acosta, D.A. Immunization of mice with Neisseria meningitidis serogroup B genomic expression libraries elicits functional antibodies and reduces the level of bacteremia in an infant rat infection model. *Vaccine* **2005**, *23*, 932-939, doi:10.1016/j.vaccine.2004.07.032.
297. Sardinas, G.; Climent, Y.; Rodriguez, Y.; Gonzalez, S.; Garcia, D.; Cobas, K.; Caballero, E.; Perez, Y.; Brookes, C.; Taylor, S., et al. Assessment of vaccine potential of the Neisseria-specific protein NMB0938. *Vaccine* **2009**, *27*, 6910-6917.
298. Bhattacharjee, A.K.; Opal, S.M.; Taylor, R.; Naso, R.; Semenuk, M.; Zollinger, W.D.; Moran, E.E.; Young, L.; Hammack, C.; Sadoff, J.C., et al. A noncovalent complex vaccine prepared with detoxified Escherichia coli J5 (Rc chemotype) lipopolysaccharide and Neisseria meningitidis Group B outer membrane protein produces protective antibodies against gram-negative bacteremia. *J Infect Dis* **1996**, *173*, 1157-1163, doi:10.1093/infdis/173.5.1157.
299. Infante, J.F.; Marrero, O.; Sifontes, S.; Sierra, G.; Campa, C.; Caro, E.; Gutiérrez, M.; Malberti, A.; Capó, V.; Fariñas, M., et al. Evaluation of the efficacy of human antimeningococcal immunoglobulin G in infant rats experimentally infected with Neisseria meningitidis group B. *Arch Med Res* **1994**, *25*, 455-461.
300. Mountzouros, K.T.; Belanger, K.A.; Howell, A.P.; Bixler, G.S., Jr.; Madore, D.V. A glycoconjugate vaccine for Neisseria meningitidis induces antibodies in human infants that afford protection against meningococcal bacteremia in a neonate rat challenge model. *Infect Immun* **2002**, *70*, 6576-6582, doi:10.1128/iai.70.12.6576-6582.2002.
301. Toropainen, M.; Saarinen, L.; Wedege, E.; Bolstad, K.; Mäkelä, P.H.; Käyhty, H. Passive protection in the infant rat protection assay by sera taken before and after vaccination of teenagers with serogroup B meningococcal outer membrane vesicle vaccines. *Vaccine* **2005**, *23*, 4821-4833, doi:10.1016/j.vaccine.2005.05.005.
302. Jäkel, A.; Plested, J.S.; Hoe, J.C.; Makepeace, K.; Gidney, M.A.; Lacelle, S.; St Michael, F.; Cox, A.D.; Richards, J.C.; Moxon, E.R. Naturally-occurring human serum antibodies to inner core lipopolysaccharide epitopes of Neisseria meningitidis protect against invasive meningococcal disease caused by isolates displaying homologous inner core structures. *Vaccine* **2008**, *26*, 6655-6663, doi:10.1016/j.vaccine.2008.09.041.
303. Heydarian, M.; Rühl, E.; Rawal, R.; Kozjak-Pavlovic, V. Tissue Models for Neisseria gonorrhoeae Research-From 2D to 3D. *Front Cell Infect Microbiol* **2022**, *12*, 840122, doi:10.3389/fcimb.2022.840122.

304. Taylor-Robinson, D.; Whytock, S.; Green, C.; Carney Jr, F. Effect of Neisseria gonorrhoeae on human and rabbit oviducts. *British Journal of Venereal Diseases* **1974**, *50*, 279.
305. Ward, M.E.; Watt, P.J.; Robertson, J.N. The human fallopian tube: a laboratory model for gonococcal infection. *J.Infect.Dis.* **1974**, *129*, 650-659.
306. Johnson, A.P.; Taylor-Robinson, D.; McGee, Z. Species specificity of attachment and damage to oviduct mucosa by Neisseria gonorrhoeae. *Infection and immunity* **1977**, *18*, 833-839.
307. Melly, M.A.; Gregg, C.R.; McGee, Z.A. Studies of toxicity of *Neisseria gonorrhoeae* for human fallopian tube mucosa. *J Infect Dis* **1981**, *143*, 423-431.
308. McGee, Z.A.; Johnson, A.P.; Taylor-Robinson, D. Pathogenic mechanisms of *Neisseria gonorrhoeae*: observations on damage to human fallopian tube mucosa in organ culture by gonococci of colony type 1 or type 4. *J.Infect.Dis.* **1981**, *143*, 413-422.
309. Maisey, K.; Nardocci, G.; Imarai, M.; Cardenas, H.; Rios, M.; Croxatto, H.B.; Heckels, J.E.; Christodoulides, M.; Velasquez, L.A. Expression of pro-inflammatory cytokines and receptors by human fallopian tubes in organ culture following challenge with *Neisseria gonorrhoeae*. *Infection and Immunity* **2003**, *71*, 527-532.
310. Lenz, J.D.; Dillard, J.P. Pathogenesis of Neisseria gonorrhoeae and the Host Defense in Ascending Infections of Human Fallopian Tube. *Front Immunol* **2018**, *9*, 2710, doi:10.3389/fimmu.2018.02710.
311. Kessler, M.; Hoffmann, K.; Fritsche, K.; Brinkmann, V.; Mollenkopf, H.-J.; Thieck, O.; Teixeira da Costa, A.R.; Braicu, E.I.; Sehouli, J.; Mangler, M. Chronic Chlamydia infection in human organoids increases stemness and promotes age-dependent CpG methylation. *Nature Communications* **2019**, *10*, 1194.
312. Tjia, K.; Van Putten, J.; Pels, E.; Zanen, H. The interaction between Neisseria gonorrhoeae and the human cornea in organ culture: an electron microscopic study. *Graefe's archive for clinical and experimental ophthalmology* **1988**, *226*, 341-345.
313. Foster, J.W.; Wahlin, K.; Adams, S.M.; Birk, D.E.; Zack, D.J.; Chakravarti, S. Cornea organoids from human induced pluripotent stem cells. *Scientific reports* **2017**, *7*, 41286.
314. Rajaiya, J.; Zhou, X.; Barequet, I.; Gilmore, M.S.; Chodosh, J. Novel model of innate immunity in corneal infection. *In Vitro Cellular & Developmental Biology-Animal* **2015**, *51*, 827-834.
315. Priyadarsini, S.; Nicholas, S.E.; Karamichos, D. 3D stacked construct: a novel substitute for corneal tissue engineering. *Sphingosine-1-Phosphate: Methods and Protocols* **2018**, 173-180.
316. Zhang, C.; Du, L.; Sun, P.; Shen, L.; Zhu, J.; Pang, K.; Wu, X. Construction of tissue-engineered full-thickness cornea substitute using limbal epithelial cell-like and corneal endothelial cell-like cells derived from human embryonic stem cells. *Biomaterials* **2017**, *124*, 180-194.
317. Hutcheon, A.E.; Zieske, J.D.; Guo, X. 3D in vitro model for human corneal endothelial cell maturation. *Experimental eye research* **2019**, *184*, 183-191.
318. Sharif, R.; Priyadarsini, S.; Rowsey, T.G.; Ma, J.-X.; Karamichos, D. Corneal tissue engineering: An in vitro model of the stromal-nerve interactions of the human cornea. *Journal of visualized experiments: JoVE* **2018**.
319. Karamichos, D.; Zareian, R.; Guo, X.; Hutcheon, A.E.; Ruberti, J.W.; Zieske, J.D. Novel in vitro model for keratoconus disease. *Journal of functional biomaterials* **2012**, *3*, 760-775.
320. Park, J.H.; Moon, S.-H.; Kang, D.H.; Um, H.J.; Kang, S.-S.; Kim, J.Y.; Tchah, H. Diquafosol sodium inhibits apoptosis and inflammation of corneal epithelial cells via activation of Erk1/2 and RSK: in vitro and in vivo dry eye model. *Investigative Ophthalmology & Visual Science* **2018**, *59*, 5108-5115.
321. Timmerman, M.M.; Shao, J.Q.; Apicella, M.A. Ultrastructural analysis of the pathogenesis of Neisseria gonorrhoeae endometrial infection. *Cellular Microbiology* **2005**, *7*, 627-636.
322. Laniewski, P.; Gomez, A.; Hire, G.; So, M.; Herbst-Kralovetz, M.M. Human Three-Dimensional Endometrial Epithelial Cell Model To Study Host Interactions with Vaginal Bacteria and Neisseria gonorrhoeae. *Infect Immun* **2017**, *85*, doi:10.1128/IAI.01049-16.

323. Wang, L.-C.; Yu, Q.; Edwards, V.; Lin, B.; Qiu, J.; Turner, J.R.; Stein, D.C.; Song, W. Neisseria gonorrhoeae infects the human endocervix by activating non-muscle myosin II-mediated epithelial exfoliation. *PLoS Pathogens* **2017**, *13*, e1006269.
324. Yu, Q.; Wang, L.-C.; Di Benigno, S.; Gray-Owen, S.D.; Stein, D.C.; Song, W. Neisseria gonorrhoeae infects the heterogeneous epithelia of the human cervix using distinct mechanisms. *PLoS pathogens* **2019**, *15*, e1008136.
325. Lõhmussaar, K.; Oka, R.; Valle-Inclan, J.E.; Smits, M.H.; Wardak, H.; Korving, J.; Begthel, H.; Proost, N.; van de Ven, M.; Kranenburg, O.W. Patient-derived organoids model cervical tissue dynamics and viral oncogenesis in cervical cancer. *Cell Stem Cell* **2021**, *28*, 1380-1396. e1386.
326. Turco, M.Y.; Gardner, L.; Hughes, J.; Cindrova-Davies, T.; Gomez, M.J.; Farrell, L.; Hollinshead, M.; Marsh, S.G.; Brosens, J.J.; Critchley, H.O. Long-term, hormone-responsive organoid cultures of human endometrium in a chemically defined medium. *Nature cell biology* **2017**, *19*, 568-577.
327. Abbas, Y.; Brunel, L.G.; Hollinshead, M.S.; Fernando, R.C.; Gardner, L.; Duncan, I.; Moffett, A.; Best, S.; Turco, M.Y.; Burton, G.J. Generation of a three-dimensional collagen scaffold-based model of the human endometrium. *Interface Focus* **2020**, *10*, 20190079.
328. Stevens, J.S.; Gray, M.C.; Morisseau, C.; Criss, A.K. Endocervical and neutrophil lipoxygenases coordinate neutrophil transepithelial migration to Neisseria gonorrhoeae. *The Journal of Infectious Diseases* **2018**, *218*, 1663-1674.
329. Chakir, J.; Pagé, N.; Hamid, Q.; Laviolette, M.; Boulet, L.P.; Rouabhia, M. Bronchial mucosa produced by tissue engineering: a new tool to study cellular interactions in asthma. *Journal of allergy and clinical immunology* **2001**, *107*, 36-40.
330. Paquette, J.-S.; Moulin, V.; Tremblay, P.; Bernier, V.; Boutet, M.; Laviolette, M.; Auger, F.A.; Boulet, L.-P.; Goulet, F. Tissue-engineered human asthmatic bronchial equivalents. *Eur Cell Mater* **2004**, *7*, 11.
331. Choe, M.M.; Sporn, P.H.; Swartz, M.A. An in vitro airway wall model of remodeling. *American journal of physiology-Lung cellular and molecular physiology* **2003**, *285*, L427-L433.
332. Choe, M.M.; Tomei, A.A.; Swartz, M.A. Physiological 3D tissue model of the airway wall and mucosa. *Nature protocols* **2006**, *1*, 357-362.
333. Pageau, S.C.; Sazonova, O.V.; Wong, J.Y.; Soto, A.M.; Sonnenschein, C. The effect of stromal components on the modulation of the phenotype of human bronchial epithelial cells in 3D culture. *Biomaterials* **2011**, *32*, 7169-7180.
334. Marrazzo, P.; Maccari, S.; Taddei, A.; Bevan, L.; Telford, J.; Soriani, M.; Pezzicoli, A. 3D reconstruction of the human airway mucosa in vitro as an experimental model to study NTHi infections. *PLoS One* **2016**, *11*, e0153985.
335. Hoang, A.T.N.; Chen, P.; Juarez, J.; Sachamitr, P.; Billing, B.; Bosnjak, L.; Dahlén, B.; Coles, M.; Svensson, M. Dendritic cell functional properties in a three-dimensional tissue model of human lung mucosa. *American Journal of Physiology-Lung Cellular and Molecular Physiology* **2012**, *302*, L226-L237.
336. Harrington, H.; Cato, P.; Salazar, F.; Wilkinson, M.; Knox, A.; Haycock, J.W.; Rose, F.; Aylott, J.W.; Ghaemmaghami, A.M. Immunocompetent 3D model of human upper airway for disease modeling and in vitro drug evaluation. *Molecular pharmaceutics* **2014**, *11*, 2082-2091.
337. Steinke, M.; Gross, R.; Walles, H.; Gangnus, R.; Schütze, K.; Walles, T. An engineered 3D human airway mucosa model based on an SIS scaffold. *Biomaterials* **2014**, *35*, 7355-7362.
338. Kuehn, D.; Majeed, S.; Guedj, E.; Dulize, R.; Baumer, K.; Iskandar, A.; Boue, S.; Martin, F.; Kostadinova, R.; Mathis, C. Impact assessment of repeated exposure of organotypic 3D bronchial and nasal tissue culture models to whole cigarette smoke. *Journal of visualized experiments: JoVE* **2015**.
339. Brown, J.A.; Pensabene, V.; Markov, D.A.; Allwardt, V.; Neely, M.D.; Shi, M.; Britt, C.M.; Hoilett, O.S.; Yang, Q.; Brewer, B.M. Recreating blood-brain barrier physiology and structure on chip: A novel neurovascular microfluidic bioreactor. *Biomicrofluidics* **2015**, *9*, 054124.

340. Cho, H.; Seo, J.H.; Wong, K.H.; Terasaki, Y.; Park, J.; Bong, K.; Arai, K.; Lo, E.H.; Irimia, D. Three-dimensional blood-brain barrier model for in vitro studies of neurovascular pathology. *Scientific reports* **2015**, *5*, 1-9.
341. Griep, L.M.; Wolbers, F.; de Wagenaar, B.; ter Braak, P.M.; Weksler, B.B.; Romero, I.A.; Couraud, P.O.; Vermes, I.; van der Meer, A.D.; van den Berg, A. BBB on chip: microfluidic platform to mechanically and biochemically modulate blood-brain barrier function. *Biomedical microdevices* **2013**, *15*, 145-150.
342. Herland, A. van derMeer, AD; FitzGerald, EA; Park, T.-E.; Sleeboom, JJ; Ingber, DE Distinct contributions of astrocytes and pericytes to neuroinflammation identified in a 3D human blood-brain barrier on a chip. *PLoS One* **2016**, *11*, e0150360.
343. Huang, B.; Peng, J.; Huang, X.; Liang, F.; Wang, L.; Shi, J.; Yamada, A.; Chen, Y. Generation of interconnected neural clusters in multiscale scaffolds from human-induced pluripotent stem cells. *ACS Applied Materials & Interfaces* **2021**, *13*, 55939-55952.
344. Wang, Y.I.; Abaci, H.E.; Shuler, M.L. Microfluidic blood–brain barrier model provides in vivo-like barrier properties for drug permeability screening. *Biotechnology and bioengineering* **2017**, *114*, 184-194.
345. Campisi, M.; Shin, Y.; Osaki, T.; Hajal, C.; Chiono, V.; Kamm, R.D. 3D self-organized microvascular model of the human blood-brain barrier with endothelial cells, pericytes and astrocytes. *Biomaterials* **2018**, *180*, 117-129.
346. Endres, L.M.; Schubert-Unkmeir, A.; Kim, B.J. Neisseria meningitidis Infection of Induced Pluripotent Stem-Cell Derived Brain Endothelial Cells. *Journal of visualized experiments: JoVE* **2020**.
347. Lauranzano, E.; Campo, E.; Rasile, M.; Molteni, R.; Pizzocri, M.; Passoni, L.; Bello, L.; Pozzi, D.; Pardi, R.; Matteoli, M. A microfluidic human model of blood–brain barrier employing primary human astrocytes. *Advanced Biosystems* **2019**, *3*, 1800335.
348. Endres, L.M.; Jungblut, M.; Divyapicigil, M.; Sauer, M.; Stigloher, C.; Christodoulides, M.; Kim, B.J.; Schubert-Unkmeir, A. Development of a multicellular in vitro model of the meningeal blood-CSF barrier to study Neisseria meningitidis infection. *Fluids Barriers CNS* **2022**, *19*, 81, doi:10.1186/s12987-022-00379-z.
349. Plested, J.S.; Welsch, J.A.; Granoff, D.M. Ex vivo model of meningococcal bacteremia using human blood for measuring vaccine-induced serum passive protective activity. *Clinical and Vaccine Immunology* **2009**, *16*, 785-791.
350. Oliver, R.; Staples, K.J.; Heckels, J.; Rossetti, C.; Molteni, M.; Christodoulides, M. Coadministration of the cyanobacterial lipopolysaccharide antagonist CyP with antibiotic inhibits cytokine production by an in vitro meningitis model infected with Neisseria meningitidis. *Journal of Antimicrobial Chemotherapy* **2012**, *67*, 1145-1154.
351. Strobel, L.; Johswich, K.O. Anticoagulants impact on innate immune responses and bacterial survival in whole blood models of Neisseria meningitidis infection. *Scientific reports* **2018**, *8*, 10225.
352. Belair, D.G.; Whisler, J.A.; Valdez, J.; Velazquez, J.; Molenda, J.A.; Vickerman, V.; Lewis, R.; Daigh, C.; Hansen, T.D.; Mann, D.A. Human vascular tissue models formed from human induced pluripotent stem cell derived endothelial cells. *Stem cell reviews and reports* **2015**, *11*, 511-525.
353. Sivarapatna, A.; Ghaedi, M.; Xiao, Y.; Han, E.; Aryal, B.; Zhou, J.; Fernandez-Hernando, C.; Qyang, Y.; Hirschi, K.K.; Niklason, L.E. Engineered microvasculature in PDMS networks using endothelial cells derived from human induced pluripotent stem cells. *Cell transplantation* **2017**, *26*, 1365-1379.
354. Miller, J.S.; Stevens, K.R.; Yang, M.T.; Baker, B.M.; Nguyen, D.-H.T.; Cohen, D.M.; Toro, E.; Chen, A.A.; Galie, P.A.; Yu, X. Rapid casting of patterned vascular networks for perfusable engineered three-dimensional tissues. *Nature materials* **2012**, *11*, 768-774.
355. Kolesky, D.B.; Truby, R.L.; Gladman, A.S.; Busbee, T.A.; Homan, K.A.; Lewis, J.A. 3D bioprinting of vascularized, heterogeneous cell-laden tissue constructs. *Advanced materials* **2014**, *26*, 3124-3130.
